# Molecular diversity of benthic ctenophores (Coeloplanidae)

**DOI:** 10.1038/s41598-017-06505-4

**Published:** 2017-07-25

**Authors:** Ada Alamaru, Bert W. Hoeksema, Sancia E. T. van der Meij, Dorothée Huchon

**Affiliations:** 1 Department of Zoology, George S. Wise Faculty of Life Sciences, Tel-Aviv University, Tel-Aviv, 69978 Israel; 2 Naturalis Biodiversity Center, P.O.Box 9517, 2300 RA Leiden, The Netherlands; 3 Oxford University Museum of Natural History, University of Oxford, Parks Road, Oxford, OX1 3PW United Kingdom; 4 Steinhardt Museum of Natural History, Israel National Center for Biodiversity Studies, Tel-Aviv University, Tel-Aviv, 6997801 Israel

## Abstract

Coeloplanidae, the largest family of benthic ctenophores, comprises 33 species, all described based on traditional morphological characteristics, such as coloration, length, and number of aboral papillae, which are highly variable and can be affected by fixation methods and environmental conditions. Thus, there is a need for reliable genetic markers to complement the morphological identifications at the species level. Here, we analyzed 95 specimens from 11 morphologically distinct species of benthic ctenophores from the Red Sea and Sulu Sea, and tested selected regions of four genetic markers (ITS1, 18S rRNA, 28S rRNA and COI) for their ability to differentiate between species. We show that the barcoding region of the mitochondrial gene, cytochrome oxidase subunit I (COI), is highly variable among species of Coeloplanidae, and effectively discriminates between species in this family. The average Kimura-2-parameter (K2P) distance between species-level clades was 10%, while intraspecific variation was ~30 times lower (0.36%). COI-based phylogeny supported the delineation of four recently described new species from the Red Sea. The other nuclear markers tested were found to be too conserved in order to separate between species. We conclude that COI is a potential molecular barcode for the family Coeloplanidae and suggest to test it in pelagic ctenophores.

## Introduction

Ctenophores represent a distinct phylum of invertebrates found in all marine environments. Most species within the phylum are planktonic gelatinous organisms, except the order Platyctenida, which is comprised of species that are benthic as adults and resemble flat worms (excluding the genus *Ctenoplana* Korotneff, 1886, which is also planktonic at the adult stage), and is composed of five different families (Coeloplanidae, Ctenoplanidae, Tjalfiellidae, Lyroctenidae and Savangiidae), with Coeloplanidae being the most species-rich^1^. To date, there are 33 known species within Coeloplanidae, belonging to two genera, *Coeloplana* and *Vallicula*, all described based on classical taxonomic criteria, which mainly rely on morphological features^2^, including pigmentation pattern, number and arrangement of aboral papillae, maximal length along tentacular axis, location and shape of tentacular sheath, the presence of oral lappets, and an oral groove^2–6^. Some of these morphological characteristics have been shown to be controversial or unreliable, as they tend to change depending on the individual’s state (e.g., relaxed versus contracted), the fixation method, or the environmental conditions^2^. As morphological characters used to designate species are altered post fixation, there is a need to develop molecular markers to aid in species descriptions and specimen identification for this group. The combination of such molecular markers and photographic records of live specimens will result in a more efficient and precise method for benthic ctenophore species delineation.

To date, only 16 annotated sequences belonging to seven different species of benthic ctenophores from the order Platyctenida are available in the Nucleotide database of the National Center for Biotechnology Information (NCBI/GeneBank). These mainly consist of the 18S rDNA gene, while other genes, including the barcoding marker cytochrome oxidase subunit I (COI), have been sequenced for only two species (Table S1). Unfortunately, the 18S rDNA is a highly conserved gene in ctenophores^7^ and is therefore not suitable for distinguishing between species or even genera in this phylum. COI is currently the most prevalent genetic barcode used for species identification in Metazoans^8^, with almost five million sequences stored in the Barcode of Life Database (BOLD, http://www.boldsystems.org/). Considering the extremely fast evolution rate of the mitochondrial genomes of the pelagic ctenophores’ *Mnemiopsis* ^9^ and *Pleurobrachia* ^10^, COI emerges as a promising tool for ctenophore barcoding. However, only one study has focused on COI variation in Ctenophora^11^, but the barcoding region of the COI gene was not examined. Thus, with only two COI sequences available for the Coeloplanidae, and only eight species barcodes for the entire Ctenophora phylum, the utility of this gene as a species level identification barcode remains to be evaluated.

Here, we investigated, for the first time, the molecular diversity of benthic ctenophores (family Coeloplanidae) using four molecular markers with different evolutionary rates, namely 18S rDNA, the C1-D2 domain of the 28S rDNA, the Internal Transcribed Spacer 1 (ITS1) and the barcoding regions of the mitochondrial gene COI. We tested the validity of these genes as molecular markers using various species from the Gulf of Aqaba (Red Sea) that were recently described^2^, as well as two additional unidentified species collected off northern Borneo (Sulu Sea). Despite its relatively low genetic divergence rate in ctenophores, the nuclear marker 18S rDNA was first analyzed allowing us to verify that our specimens are indeed ctenophores from the Coeloplanidae family. We chose to amplify the C1-D2 domain of the nuclear 28S rDNA because this marker has been found to be phylogenetically informative in other animal groups (e.g., Porifera)^12^. The hyper-variable ITS1 had already been sequenced for a few specimens^7, 13^ and is commonly used as an alternative barcoding marker in several groups^14^, when higher resolutions of genetic relationships are examined (i.e., species delineation, population genetics). The last marker tested was the mitochondrial COI gene, which is known as the universal barcode, commonly used in molecular systematic studies.

## Results

### Phylogenetic analyses of the nuclear 18S rDNA marker

**Figure 1:**
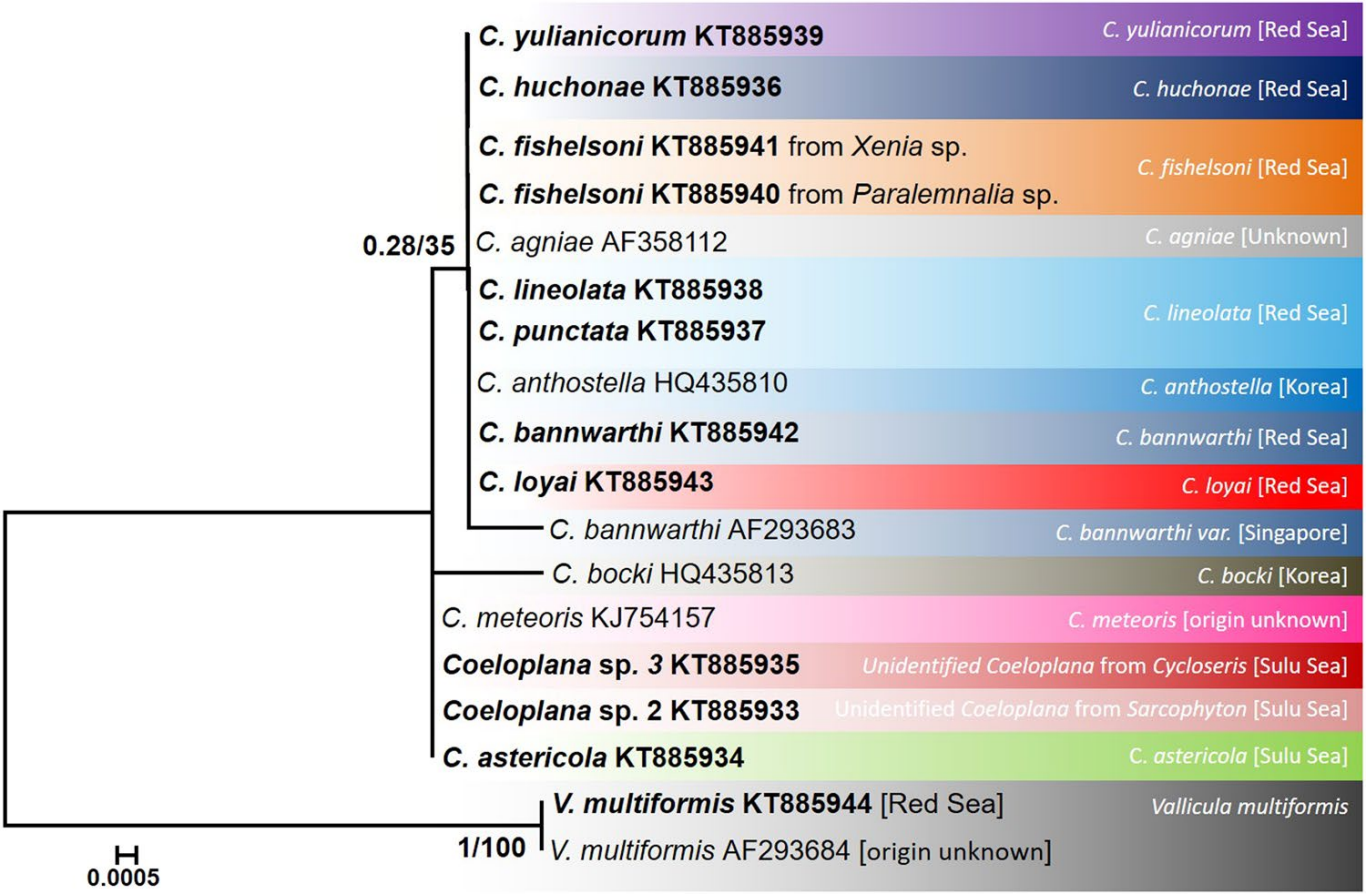
18S rDNA maximum likelihood tree. The phylogenetic reconstruction was based on 1,746 bp from the 18S rDNA gene. ML bootstrap support/Bayesian posterior probabilities are indicated near the corresponding nodes. Sequences generated in the framework of this study are highlighted in bold. Coeloplanidae species considered valid based on the COI analysis are indicated by different colors.

The final alignment of the 18S rDNA sequences contained 1,746 positions, of which 1,712 were constant, 32 were variable and 27 were parsimony-informative. The average *p*-distance between *Coeloplana* species was 0.03 ± 0.007% SE, ranging between 0.0–0.21%. This marker could not differentiate between species from the genus *Coeloplana* (Fig. 1, Table 1). The average *p*-distance between genera (i.e., *Coeloplana* vs. *Vallicula*) was 1.5 ± 0.03% SE.

**Table 1:** 18S rDNA interspecific sequence divergences (*p*-distances) of ctenophore species from the family Coeloplanidae.

		1	2	3	4	5	6	7	8	9	10	11	12	13
1	*C*. *agniae* AF358112													
2	*C*. *anthostella* HQ435810	0												
3	*C*. *astericola* KT885934	0	0											
4	*C*. *bannwarthi* KT885942, AF293683	0.04	0.04	0.04										
5	*C*. *bocki* HQ435813	0.17	0.17	0.17	0.21									
6	*C*. *fishelsoni* KT885940, KT885941	0	0	0	0.04	0.17								
7	*C*. *huchonae* KT885936	0	0	0	0.04	0.17	0							
8	*C*. lineolata* KT885937, KT855938	0	0	0	0.04	0.17	0	0						
9	*C*. *loyai* KT885943	0	0	0	0.04	0.17	0	0	0					
10	*C*. *meteoris* KJ754157	0	0	0	0.04	0.17	0	0	0	0				
11	*C*. *yulianicorum* KT885393	0	0	0	0.04	0.17	0	0	0	0	0			
12	*Coeloplana* sp. 3 from *Cycloseris* KT885935	0	0	0	0.04	0.17	0	0	0	0	0	0		
13	*Coeloplana* sp. 2 from *Sarcophyton* KT885933	0	0	0	0.04	0.17	0	0	0	0	0	0	0	
14	*V*. *multiformis* KT885944, AF293684	1.49	1.49	1.49	1.49	1.49	1.49	1.49	1.49	1.49	1.49	1.49	1.49	1.49

Each species is represented by a single sequence, except *C*. *bannwarthi*, *C*. *fishelsoni*, *C*. *lineolata* and *V*. *multiformis*, which are represented by two individuals each. *C. *lineolata* and *C.* *punctata* were considered to belong to the same clade.

### Phylogenetic analyses of the nuclear 28S rDNA marker

**Figure 2:**
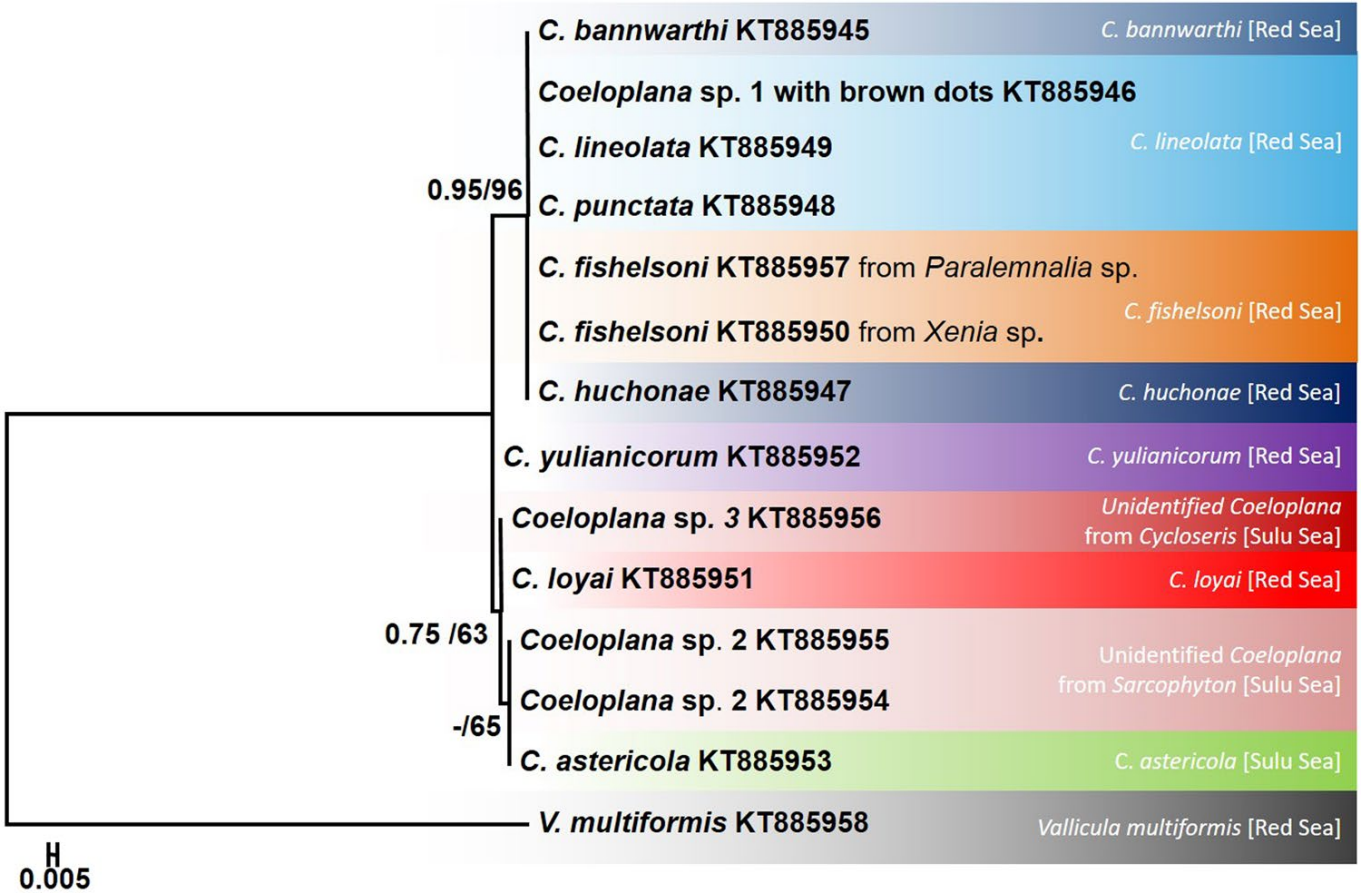
28S rDNA maximum likelihood tree. The phylogenetic reconstruction was based on 721 bp from the C1-D2 domain of the 28S rDNA gene. ML bootstrap support/Bayesian posterior probabilities are indicated near the corresponding nodes. All sequences were generated in the framework of this study. Coeloplanidae species considered valid based on the COI analysis are indicated by different colors.

The final alignment of the C1-D2 domain of the 28S rDNA sequences contained 721 positions, of which 647 were constant, 70 were variable and six were parsimony- informative. The average *p*-distance between *Coeloplana* species was 0.44 ± 0.056% SE, ranging between 0–0.84%. The average *p*-distance between genera (i.e., *Coeloplana* vs. *Vallicula*) was 9.5 ± 1% SE. Although the 28S rDNA marker was more variable than the 18S rDNA, it could not differentiate between several *Coeloplana* species (Fig. 2, Table 2).

**Table 2:** 28S rDNA (C1-D2 domain) interspecific sequence divergences (*p*-distances) of ctenophore species from the family Coeloplanidae.

		1	2	3	4	5	6	7	8	9
1	*C*. lineolata* KT885946, KT885948, KT885949									
2	*C*. *huchonae* KT885947	0								
3	*C*. *fishelsoni* KT885957, KT885950	0	0							
4	*C*. *loyai* KT558951	0.7	0.7	0.7						
5	*C*. *yulianicorum* KT885952	0.56	0.56	0.56	0.14					
6	*C*. *bannwarthi* KT885945	0	0	0	0.7	0.56				
7	*C*. *astericola* KT885953	0.84	0.84	0.84	0.14	0.28	0.84			
8	*Coeloplana* sp. 2 from *Sarcophyton* KT885954, KT885955	0.84	0.84	0.84	0.14	0.28	0.84	0		
9	*Coeloplana* sp. 3 from *Cycloseris* KT885956	0.7	0.7	0.7	0	0.14	0.7	0.14	0.14	
10	*V*. *multiformis* KT885958	9.65	9.65	9.65	9.37	9.37	9.65	9.37	9.37	9.37

Each species is represented by a single sequence, except *C*. *lineolata*, *C*. *fishelsoni* and *Coeloplana* sp. 2, which are represented by three, two and two individuals, respectively. *C. *lineolata* and C. *punctata* were considered to belong to the same clade.

### Phylogenetic analyses of the nuclear ITS1 marker

**Figure 3:**
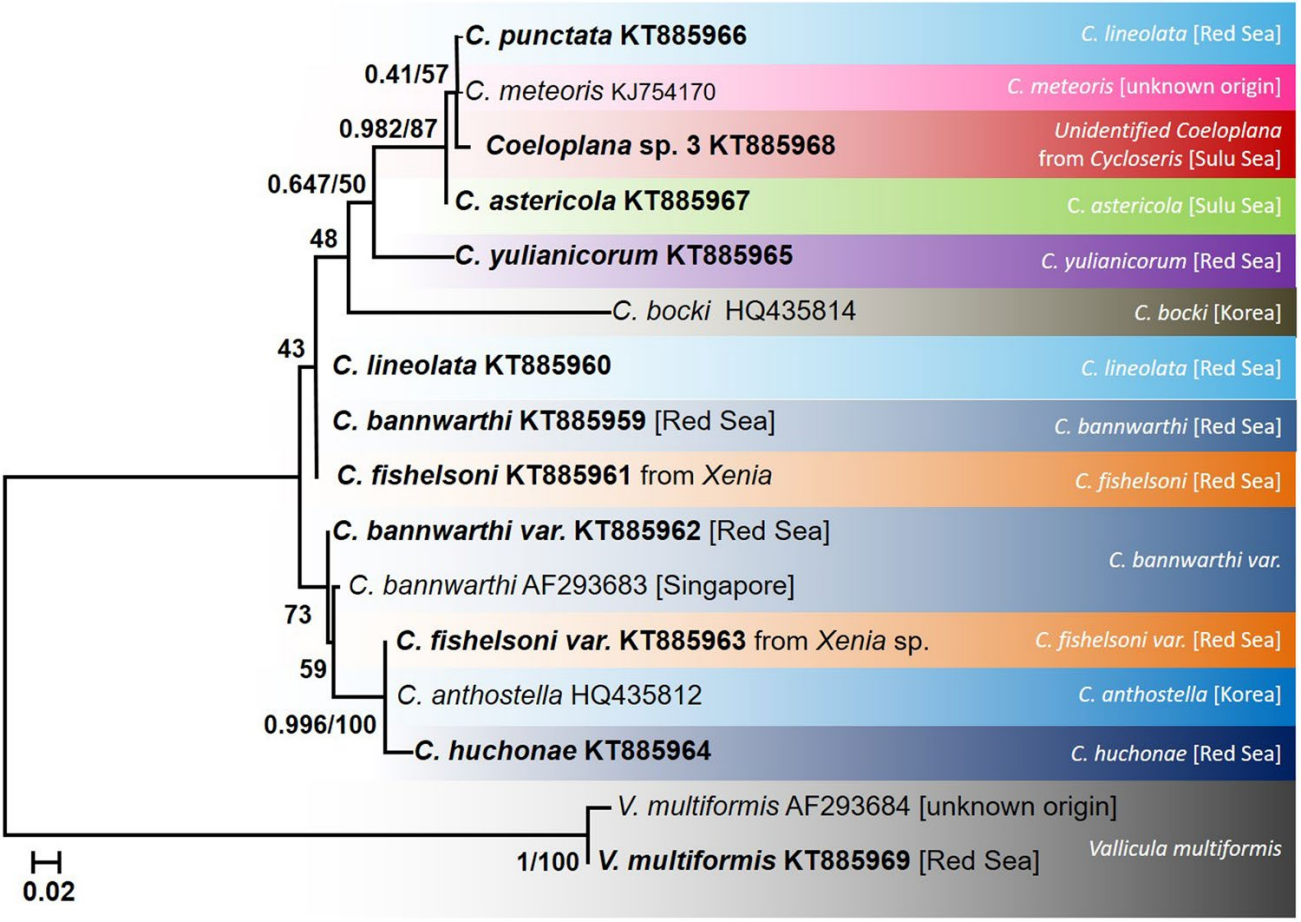
ITS1 maximum likelihood tree. The phylogenetic reconstruction was based on 397 bp from the ITS1 marker. ML bootstrap support/Bayesian posterior probabilities are indicated near the corresponding nodes. Sequences generated in the framework of this study are highlighted in bold. Coeloplanidae species considered valid based on the COI analysis, are indicated by different colors.

The final alignment of the ITS1 sequences contained 397 positions, of which 243 were constant, 130 were variable and 94 were parsimony-informative. The average *p*-distance between *Coeloplana* species was 4.7 ± 1.05% SE, ranging between 0–10%. The average *p*-distance between genera (i.e., *Coeloplana* vs. *Vallicula*) was 28.7 ± 3% SE. Although ITS1 is considered to be a hyper-variable marker, it could not differentiate between several valid *Coeloplana* species. For example, *C*. *lineolata*, *C*. *fishelsoni* and *C*. *bannwarthi* have identical ITS 1 sequence (Fig. 3). This marker, however, differentiated well between the two coeloplanid genera (Fig. 3, Table 3).

**Table 3:** ITS1 interspecific sequence divergence (*p*-distances) for ctenophores from the family Coeloplanidae.

		1	2	3	4	5	6	7	8	9	10	11	12
1	*C*. *bannwarthi* KT885959												
2	*C*. lineolata* KT885960, KT885966	3.77											
3	*C*. *fishelsoni* KT885961	2.26	1.76										
4	*C*. *bannwarthi* var. KT885962	2.26	4.77	3.52									
5	*C*. *fishelsoni* var. KT885963	3.27	6.03	5.03	1.51								
6	*C*. *huchonae* KT885964	3.77	6.53	5.53	2.01	0.5							
7	*C*. *anthostella* HQ435812	3.27	6.03	5.03	1.51	0	0.5						
8	*C*. *yulianicorum* KT885965	3.27	3.52	2.01	4.52	4.02	4.52	4.02					
9	*C*. *astericola* KT885967	4.77	1.76	3.02	5.53	6.53	7.04	6.53	4.52				
10	*C*. *meteoris* KJ754170	5.28	2.26	3.52	6.03	7.04	7.54	7.04	5.03	0.5			
11	*Coeloplana* sp. 3 from *Cycloseris* KT885968	4.77	2.26	3.52	5.03	6.03	6.53	6.03	4.02	0.5	1.01		
12	*C*. *bocki* HQ435814	8.04	7.79	6.03	9.05	9.55	10.1	9.55	6.53	9.05	9.55	9.045	
13	*V*. *multiformis* KT885969, AF293684	27.9	27.9	27.6	28.1	29.6	30.2	29.6	28.6	28.6	29.1	29.15	29.65

Each species is represented by a single sequence, except *V*. *multiformis* and *C*. *lineolata*, which are represented by two individuals each. *Coeloplana* sp. 3 was collected from *Cycloseris* corals from the Sulu Sea. *C. *lineolata* and *C*. *punctata* were considered to belong to the same clade.

### Phylogenetic analyses of the mitochondrial COI marker

**Figure 4:**
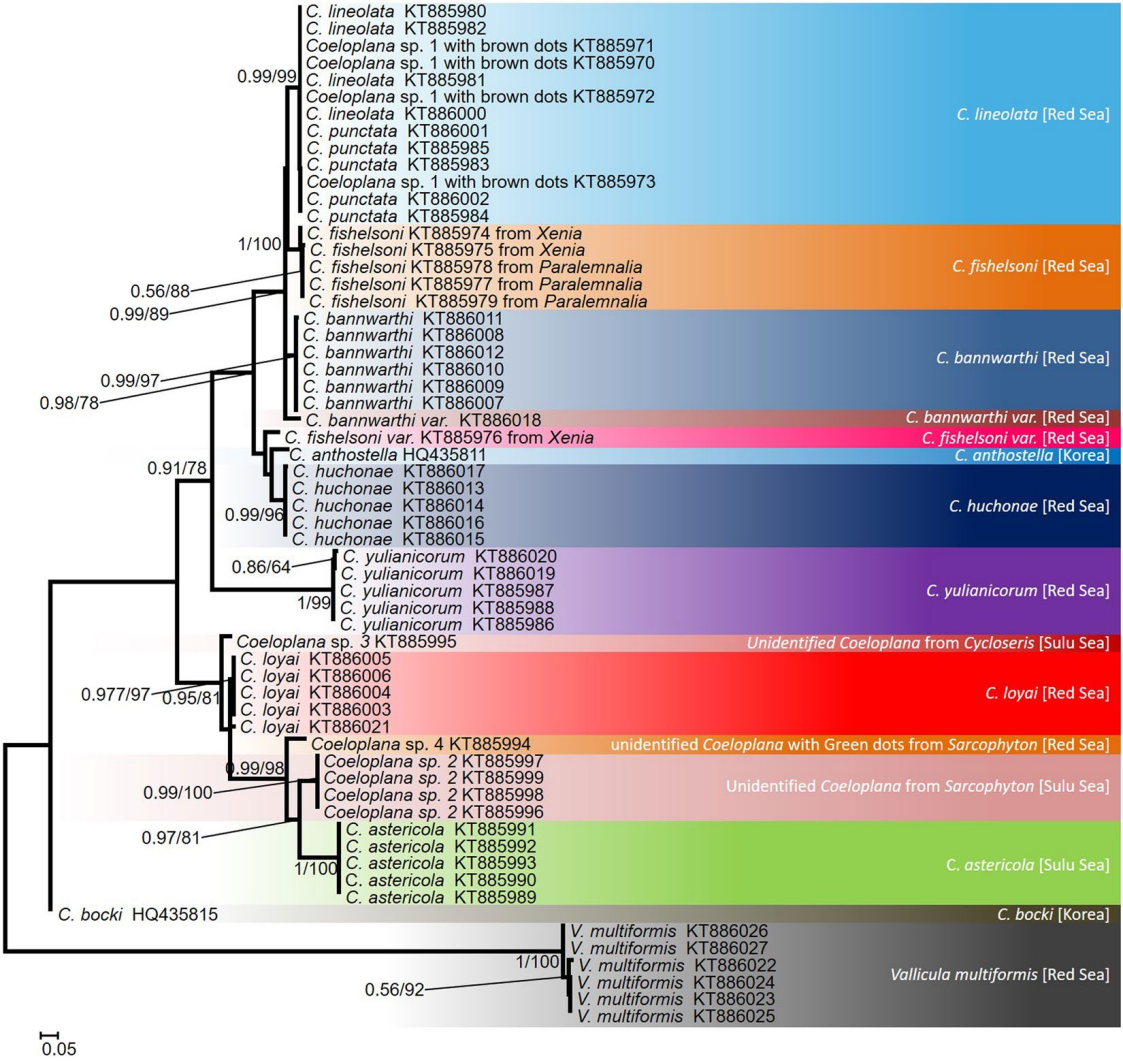
COI maximum likelihood tree. The phylogenetic reconstruction was based on 657 bp from the COI gene. ML bootstrap support/Bayesian posterior probabilities are indicated near the corresponding nodes. All sequences were generated in the framework of this study, except for *C*. *anthostella* ^37^ and *C*. *bocki* ^38^.

The final alignment of the barcoding region of the COI gene contained 657 positions, of which 375 were constant, 282 were variable, and 259 were parsimony-informative. All COI sequences were heavily AT-biased, with an average of A+T content of 72.25 ± 2.25%. Both maximum likelihood (ML) and Bayesian analyses yielded similar tree topologies with 15 well-supported clades (Fig. 4). The distinction between *Coeloplana punctata* Fricke, 1970 and *C*. *lineolata* Fricke, 1970, both associated with soft corals of the genus *Sarcophyton*, was not supported by our molecular data.

**Figure 5:**
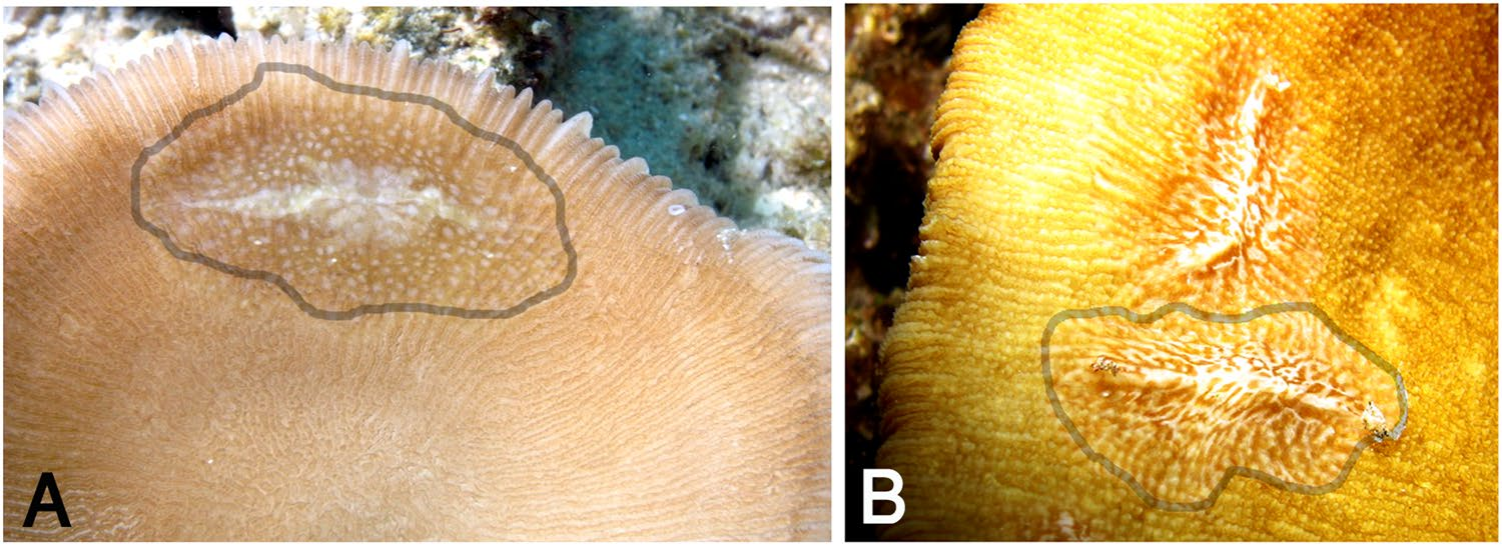
Benthic ctenophores living on the underside (aboral) of mushroom corals. (**A**) An unidentified *Coeloplana* species collected from *Cycloseris costulata* in Tun Mustapha Park, northernmost tip of Borneo, Sulu Sea; (**B**) The recently described new species *Coeloplana loyai* living on *Herpolitha limax* in the Gulf of Aqaba, Red Sea. The gray line outlines the ctenophores. (**B** Photo credit: Eran Brokovich).

The average Kimura-2-parameter (K2P) distance between *Coeloplana* species was 10 ± 0.36% SE. The minimal interspecific distance was 2.3% (between *C*. *loyai* from mushroom corals in the Red Sea and *Coeloplana* sp. 3 collected from a mushroom coral in the Sulu Sea, Fig. 5), while the maximal intraspecific distance was 0.36%. This mitochondrial marker successfully differentiated between all *Coeloplana* species analyzed, except for *C*. *punctata* and *C*. *lineolata* (see Discussion; Fig. 4, Table 4). The average K2P distance between the genera, *Coeloplana* and *Vallicula*, was 32.1% ± 2.2% SE.

**Table 4:** COI intra- and interspecific sequence divergence (K2P distances/*p*-distances) for ctenophore species from the family Coeloplanidae.

		1	2	3	4	5	6	7	8	9	10	11	12	13	14	15
1	*C*. *lineolata* (n = 13)	0.082	12.6	3.6	3.6	9.4	3.6	6.8	6.8	9.6	11.4	12.1	10.4	7.1	12	26.8
2	*C*. *astericola* (n = 5)	13.9	0	12	12.1	8.7	12.3	12.3	11.3	8.2	5	14.8	6.3	12.7	12.2	26.1
3	*C*. *bannwarthi* var. (sample 2000–11) (n = 1)	3.7	13.2	NA	3.1	9.1	3.9	5.9	6	9.1	105	11.6	10.1	6.1	11.1	26.2
4	*C*. *bannwarthi* (n = 6)	3.7	13.2	3.2	0.051	9.3	4	6.3	6.3	9.2	10.5	12.1	10.1	7	11.8	26.2
5	*Coeloplana* sp. 3 from C*ycloseris* (n = 1)	10.1	9.3	9.8	10	NA	10.7	9.3	9	2.3	6.7	12.3	6.7	10.1	9.6	24.4
6	*C*. *fishelsoni* (n = 5)	3.7	13.5	4	4.1	11.6	0.183	7.4	7.4	10.9	11.2	13.4	10.8	7.7	12.6	26.8
7	*C*. *fishelsoni* var. (sample 2011–3) (n = 1)	7.2	13.6	6.2	6.6	10	7.9	NA	3.7	9.8	10.7	12.3	11	4.1	11.9	26.4
8	*C*. *huchonae* (n = 5)	7.2	12.3	6.3	6.6	9.6	7.9	3.8	0.061	8.9	9.3	11.7	9.9	3.4	11	24.9
9	*C*. *loyai* (n = 5)	10.3	8.8	9.7	9.9	2.3	11.9	10.5	9.5	0.369	6.6	12.6	6.3	9.9	9.8	23.7
10	*Coeloplana* sp. 2 from S*arcophyton* (n = 4)	12.4	5.3	11.4	11.4	7.1	12.2	11.6	10	6.9	0	13	4.7	11	11.1	24.4
11	*C*. *yulianicorum* (n = 5)	13.3	16.8	12.7	13.4	13.6	15	13.6	12.9	14	14.4	0.214	13.6	11.5	13.2	28.6
12	*Coeloplana* sp. 4 with green dots from *Sarcophyton* (n = 1)	11.3	6.6	10.8	10.9	7.1	11.7	12	10.7	6.6	4.9	15.2	NA	11	11.3	25.3
13	*C*. *anthostella* (n = 1)	7.6	14	6.4	7.5	10.9	8.2	4.3	3.5	10.7	11.9	12.7	12	NA	11.6	25.9
14	*C*. *bocki* (n = 1)	13.1	13.5	12.1	12.9	10.3	13.8	13.1	12	10.6	12.1	14.7	12.3	12.8	NA	24.8
15	*V*. *multiformis* (n = 6)	33.3	32.1	32.4	32.3	29.6	33.3	32.7	30.4	28.5	29.6	36.1	30.9	31.9	30.1	0.6

Below diagonal: interspecific K2P divergence; Above diagonal: interspecific *p*-distances; Diagonal: Intraspecific genetic divergence marked in bold font (K2P and *p*-distance gave similar results). The number of specimens considered for each species is indicated near the sample name.

## Discussion

Though debated due to several limitations^15–17^, DNA barcoding is a useful tool for species identification and the discovery of new species^8, 18, 19^, especially when integrated with morphological taxonomy^17, 20–22^. We show that COI is a variable marker within Coeloplanidae and can thus be used to identify species in this family. Moreover, our results support the designation of four new Red Sea *Coeloplana* species that were recently described by Alamaru *et al.* ^2^.

### Suitability of the various genetic markers to distinguish between Coeloplanidae species

The 18S rDNA sequences and the C1-D2 domain of the 28S rDNA were not variable enough and failed to differentiate among species within the family Coeloplanidae, while successfully separating the two Coeloplanidae genera (*Coeloplana* and *Vallicula*), in agreement with the 18S rDNA phylogeny presented by Podar *et al*.^7^ for the phylum Ctenophora. However, the insufficient number of ctenophore 28S rDNA sequences currently available in public databases did not allow to test the utility of this marker for other groups from this phylum. The ITS1 marker, usually considered to be hyper-variable^7, 14, 23^, was not variable enough to differentiate between some *Coeloplana* species, in agreement with the recent work of Simion *et al*.^24^, who found that the ITS1 region of ctenophores is relatively conserved and can be easily aligned, even between distantly related ctenophore taxa. In addition, because ITS1 in some species includes more than one microsatellite region, it is challenging to sequence using the Sanger sequencing method. Indeed, these polymeric repeats induced *in vitro* slippage errors during amplification and sequencing reactions, thus hampering the determination of the sequences. Furthermore, in a few cases, intra-individual variation appears to occur, affecting the reliability of this marker for identification of species, as paralogous copies may be compared rather than orthologous ones^25^. We thus conclude that the ITS1 marker is not suitable for large taxonomic surveys.

In contrast, we show that the mitochondrial COI sequences have a higher divergence rate than the hyper-variable ITS1 marker, in agreement with the extremely fast evolution rate of the mitochondrial genomes of the pelagic ctenophores *Mnemiopsis leidyi* A. Agassiz, 1865^9^ and *Pleurobrachia bachei* A. Agassiz, 1860^10^. Our results show that benthic ctenophores of the family Coeloplanidae also present a high mitochondrial evolution rate, resulting in an average K2P distance of 10% between species. This high interspecific variation, along with ~30-times lower mean intraspecific variation (0.36%), emphasizes COI as an effective DNA barcode in ctenophores. Moreover, a barcoding gap^15^ was observed for all species analyzed in this study, except for the *C*. *punctata* and *C*. *lineolata* clade. The suitability of the COI gene should be further verified by analyzing additional COI sequences from various ctenophore species and populations and by considering independent nuclear markers. The only exception is the 2.3% distance between *C*. *loyai* collected from the mushroom coral *Ctenactis echinata* in the Gulf of Aqaba and an unidentified ctenophore (*Coeloplana* sp. 3) collected from another mushroom coral, *Cycloseris costulata*, off northern Borneo in the Sulu Sea^26^ (Fig. 5). Due to the poor state of preservation of the Sulu Sea sample, we could not identify it based on its morphology, and therefore cannot conclude at this stage whether the latter sample collected from *C*. *costulata* represents a different species or a member of a different population of *C*. *loyai*. The species analyzed in the framework of this study show very low intraspecific genetic variability for COI mitochondrial marker. Asexual reproduction, which is known to occur among benthic ctenophores^27^, may thus play an important role in their life history^28, 29^.

### Molecular support for the identification of four recently described *Coeloplana* species

Alamaru *et al*. recently described four new species of benthic ctenophores from the Gulf of Aqaba, Red Sea^2^: *C*. *loyai* collected from the mushroom corals *Ctenactics echinata* and *Herpolitha limax*; *C*. *yulianicorum* collected from the soft coral *Sarcophyton glaucum*; *C*. *huchonae* collected from the soft coral *Dendronephthya hemprichi*; and *C*. *fishelsoni* collected from the soft corals *Xenia umbellata* and *Paralemnalia* spp. Our current molecular analyses corroborate the designation of these ctenophores as valid species belonging to the family Coeloplanidae, as all four species show K2P distances >3% for the barcoding marker COI.

### Cryptic diversity in benthic ctenophores

One of the samples originally identified as *C*. *fishelsoni* based on its morphology^2^ (sample 2011-3 collected from *Xenia*) showed more than 7% sequence divergence compared to the other five *C*. *fishelsoni* samples. This sample (accession number KT885976) clustered closer to *C*. *anthostella* and *C*. *huchonae* in the COI-based phylogeny, rather than with other *C*. *fishelsoni* sequences. This pattern was also observed in the ITS1 tree (accession number KT885963), though this should be considered with caution since the presence of intra individual variation may affect the phylogenetic results. The same was observed for a sample originally identified as *C*. *bannwarthi* (sample number 2000-11 collected from the sea urchin *D*. *setosum*). Based on COI sequences (accession number KT886018), this specimen presented more than 3% sequence divergence compared to other *C*. *bannwarthi* specimens, and thus belongs to a different clade, a pattern also observed in the ITS1 tree (accession number KT885962). As the two diverging samples of *C*. *fishelsoni* and *C*. *bannwarthi* displayed the morphology of the described valid species, we attribute the molecular differences to a possible cryptic species diversity. These samples were thus considered as separate clades in all genetic analyses and were labeled as *C*. *fishelsoni* var. and *C*. *bannwarthi* var. in the phylogenetic trees. We also found that an unidentified specimen with green dots (*Coeloplana* sp. 4) formed a distinct clade in the COI tree, suggesting that it is another unidentified *Coeloplana* species. Although this specimen exhibited some similarities to *C*. *punctata* (i.e. identical host and pattern of multiple dots across the entire body), it differed in the color of the dots (green versus brown). These results are currently supported by a single mitochondrial marker (COI), and, even in the absence of a stop codon, we cannot exclude the amplification of a nuclear mitochondrial DNA segment (numt). Additional genetic markers, as well as careful morphological evaluation, will therefore be necessary to substantiate these cryptic species.

### Synonymy of the species *Coeloplana lineolata* and *Coeloplana punctata*

Our analysis of COI sequences supported the majority of previously designated species based on morphological features (i.e., classical taxonomy). However, COI does not differentiate between *C*. *lineolata* and *C*. *punctata,* which are currently accepted as valid species originally described by Fricke^30^ from Madagascar. Sequences of these two species cluster into a well-supported clade (Fig. 4) with a very small K2P distance of 0.15%, well within the range of COI intraspecific divergence observed for coeloplanids (Table 4). In contrast, ITS1 sequences of *C*. *lineolata* and *C*. *punctata* were extremely variable. However, as the support for the ITS1 phylogeny was generally low, these results are not reliable.

**Figure 6:**
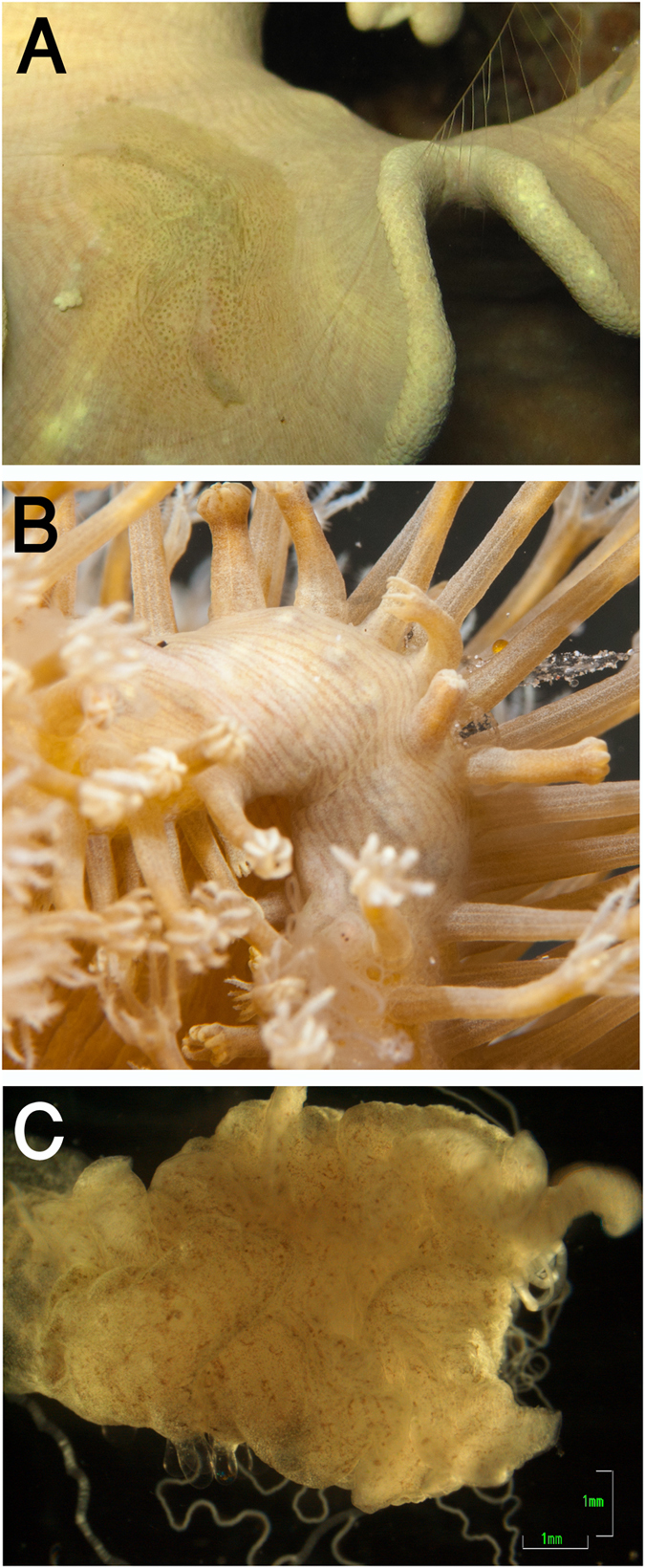
Benthic ctenophore species from the *lineolata*/*punctata* clade (**A**) *Coeloplana punctata* on its host, the soft coral *Sarcophyton*, in the Gulf of Aqaba, Red Sea; (**B**) *Coeloplana lineolata* on its host, the soft coral *Sarcophyton*; (**C**) A specimen originally assigned as “brown dots” or *Coeloplana* sp. 1, as it was challenging to identify based on morphology, which comprises of both brown dots and a somewhat parallel lines pattern. Photos credit: Eran Brokovich.

It some cases, it was challenging to morphologically differentiate *C*. *lineolata* from *C*. *punctata*. Indeed, the parallel lines in contracted individuals appeared as dots in relaxed individuals (Fig. 6). When species identification was ambiguous, the specimens were categorized as “brown dots” or *Coeloplana* sp. 1, which in later COI molecular analysis clustered with the *C*. *lineolata* and *C*. *punctata* clade (Figs 4 and 6). Our combined phylogenetic analysis and morphological observations suggest that there is no support for the designation of two different species. Additional molecular data using different markers and samples from the type locality will be needed to validate this conclusion.

### Sulu Sea species

**Figure 7:**
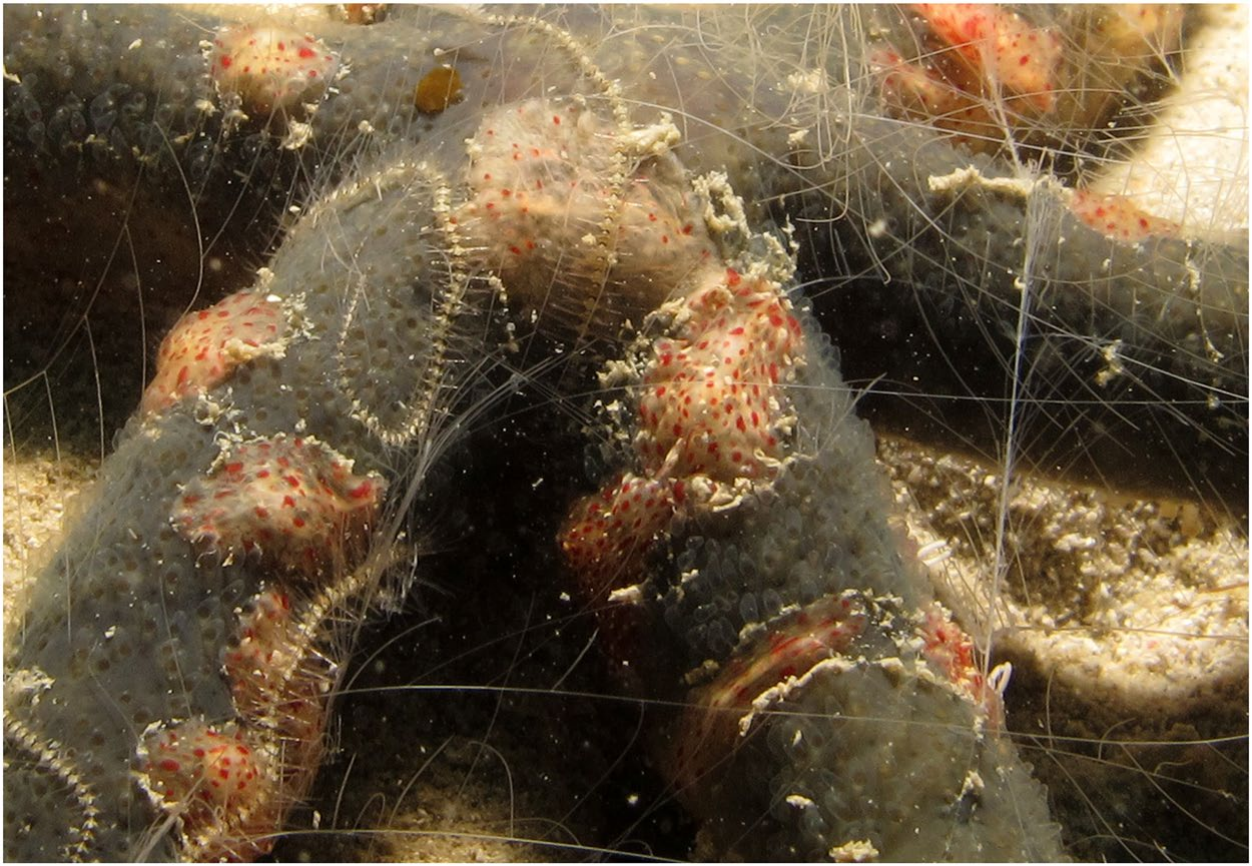
*Coeloplana astericola* collected from its host, the sea star *Echinaster* sp., in Tun Mustapha Park, northernmost tip of Borneo, Sulu Sea.

The samples collected from the sea star *Echinaster* sp. in the Sulu Sea clustered into one clade with 100% bootstrap support. Based on photos of the sampled specimens (Fig. 7), we suggest this clade to represent *C*. *astericola* Mortensen 1927. The average K2P distance between *Coeloplana* specimens sampled from *Echinaster* sea stars and *Coeloplana* specimens sampled from *Sarcophyton* corals was 5.3%, suggesting that these two clades belong to different species. *Coeloplana* sampled from *Sarcophyton* corals off Borneo (*Coeloplana* sp. 2) may either belong to one of the two un-sequenced species known to live on *Sarcophyton* (*C*. *wuennenbergi* Fricke, 1970 or *C*. *mellosa* Gershwin, Zeidler and Davie, 2010) or constitute a completely new species. Unfortunately, the ethanol fixation of the samples for molecular analysis caused major morphological deformities, thus precluding a morphological description, as well as assignment to either a valid or a new species. Further sampling and inspection of live material would resolve their taxonomic status.

### No obvious cospeciation of Coeloplanidae species and their hosts

We found no evidence of cospeciation between *Coeloplana* species and their hosts. The three species that live on *Sarcophyton glaucum* in the Red Sea (*Coeloplana lineolata*, *C*. *yulianicorum* and the unidentified *Coeloplana* sp. 4 with green dots) are not closely related to each other, nor to the *Coeloplana* sp. 2 collected from *Sarcophyton* sp. in the Sulu Sea. Similarly, the two species living on echinoderms (i.e., *C*. *bannwarthi* living on the sea urchin *Diadema setosum* and *C*. *astericola* living on sea stars of the genus *Echinaster*) are not closely related. Moreover, the two *Coeloplana* species sampled off Borneo (*C*. *astericola* found on echinoderms and the unidentified *Coeloplana* sp. 2 from *Sarcophyton*) clustered according to geography, regardless of their host.

## Conclusions

Based on our results, we conclude that COI is a suitable barcode for benthic ctenophores from the family Coeloplanidae. We suggest testing the utility of this mitochondrial marker on other families and orders in the phylum Ctenophora. COI may prove to be especially useful in the delineation of pelagic ctenophore species known to be very fragile and challenging to preserve. It is possible, however, that the traditional Folmer primers^31^ might not be suitable because ctenophores seem to have extremely high rates of mitochondrial evolution. Our results support the designation of four new *Coeloplana* species recently reported^2^. Based on the COI phylogenetic reconstruction and on previous morphological descriptions, we suggest that *C*. *punctata* and *C*. *lineolata* might belong to the same species. We conclude that the *Coeloplana* sampled from *Sarcophyton* corals off Borneo could constitute a new, undescribed species. Comprehensive morphological examination of this species requires further sampling using adjusted fixation protocols. Our molecular results suggest the presence of cryptic benthic ctenophore species in the Red Sea. Finally, we found no indication of cospeciation between *Coeloplana* species and their hosts. Our molecular study indicates that *Coeloplana* is a highly diverse genus, which can be effectively differentiated into species using the COI marker. As this group is cryptic and poorly studied, we assume that many species remain to be described.

## Methods

### Collection and observation

Benthic ctenophore species were collected in 2012 and 2013 from various invertebrates and algae by scuba diving along the Israeli shore of the Gulf of Aqaba (29°30′ N, 34°56′ E) (permit 2010/37891 issued by the Israel Nature and Park Authority) and from Tun Mustapha Park, Sabah, Malaysia (permits granted by the Economic Planning Unit, Prime Minister’s Department Malaysia and Sabah Biodiversity Centre for the Tun Mustapha Park Expedition to Zarinah Waheed^32^. In the Red Sea, sampling was done mainly at night, as most benthic ctenophore species were easier to locate due to their extended tentacles and better contrast with the background water, whereas off Borneo, ctenophores were sampled during daytime dives. Some specimens were collected together with their hosts, and dislodged from them later in the lab using a pipette with a gentle stream of sea water. In other cases, the ctenophores were dislodged from their hosts *in situ* using a small pipette.

Each ctenophore encountered was photographed *in situ*. For each specimen collected, the date, site, depth, and host were registered. Due to the expected difficulties, which are inherent to the morphological examination of fixed material, each collected live specimen was inspected in the lab and photographed using a high-resolution camera mounted on a stereoscope. Field circumstances did not allow for this procedure to be followed off Borneo. The species identification was conducted based on all existing Coeloplanidae literature, as previously reviewed^2^. Following identification and documentation, whole specimens were preserved in 95% EtOH for molecular analysis.

### DNA sequencing

Genomic DNA was extracted from individual ctenophores preserved in 95% EtOH using the Qiagen Blood & Tissue kit (Venlo, Netherlands) according to the manufacturer instructions. Genomic DNA was used for PCR amplification of four genetic markers (for details and primer sequences see Table S2).

All PCR reactions were performed on a TProfessional Basic (Biometra, Göttingen, Germany) in 25 µl total reaction volume containing 2 µl of DNA template (~50 ng), 2.5 µl of 10X ExTaq^TM^ buffer, 2 µl of dNTPs supplied with ExTaq kit (2.5 mM each), 0.2 µl of TaKaRa ExTaq^TM^ polymerase (5 units/µl), 5 µl of Betaine (5 M), 0.25 µl of DMSO, 2.5 µl of each primer (5 pM) and 10.5 µl of sterile H_2_O. PCR reactions were performed under the following conditions: (1) for COI: 95 °C for 3 minutes followed by 35 cycles of 1 min at 95 °C, 1 min at 40 °C, 1.30 min at 72 °C, and a final extension of 20 min at 72 °C; (2) for 18S rDNA: 95 °C for 3 minutes followed by 30 cycles of 1 min at 94 °C, 1 min at 50 °C, 2 min at 72 °C, and a final extension of 20 min at 72 °C; (3) for 28S rDNA: 94 °C for 3 minutes followed by 39 cycles of 40 sec at 94 °C, 40 sec at 55 °C, 1.30 min at 72 °C, and a final extension of 10 min at 72 °C; (4) for ITS1: 94 °C for 1 minute followed by 35 cycles of 30 sec at 94 °C, 30 sec at 48 °C, 2 min at 72 °C, and a final extension of 10 min at 72 °C. Amplicons were purified either with the QIAquick® PCR purification kit (Qiagen, Venlo, Netherlands) according to the manufacturer instructions, or by using Polyethylene glycol (PEG) in saline (NaCl). Purified PCR products were directly sequenced using Big Dye Terminator v1.1 (Applied Biosystems) on an ABI 310/410 sequencer. The sequences generated in the framework of this study have been submitted to NCBI under the accession numbers KT885933- KT886027.

### Sequence alignment

New sequences generated in this study were aligned with available sequences of platyctenids from public databases (Table S1). For the rRNA and ITS datasets, alignment was performed under the L-INS-I algorithm of MAFFT v7.017^33^ as implemented in Geneious 6.1.8 (www.geneious.com). For the COI dataset, a translation alignment was performed with the available CDSs using the same algorithm and program.

### Phylogenetic analysis

Phylogenetic analyses were performed for each gene dataset separately using both the ML and the Bayesian criteria. ML analyses were performed with RAxML v8.0.26^34^ under the GTRGAMMA model. Specifically, the tree searches were conducted with 100 runs. Branch supports were computer based on 1,000 slow bootstrap replicates. In addition, the ML analysis of the COI gene was performed using a codon partition.

Bayesian analyses were performed with Mr Bayes version 3.2.6^35^ under the GTRGAMMAI model. For each dataset, two runs with four chains each were conducted, with default temperatures and default prior distributions. The chains were run for 10,000,000 generations and sampled every 100 generations. Model parameters were allowed to be optimized independently for each codon position partition. Convergence was achieved before 2,500,000 generations for all markers (i.e., standard deviation of split frequencies was verified to have reached 0.009). The first 2,500,000 generations were thus discarded for all markers (burnin), and the Bayesian consensus was computed based on 150,000 trees.

Inter- and intraspecific genetic variabilities were computed using MEGA6^36^. For the rRNAs and the ITS datasets, pairwise *p*-distances were computed between each pair of sequences (each species was represented by a single sequence) (Tables 1–3). For the COI gene, both average pairwise K2P distances and average *p*-distances were calculated (Table 4). Variance estimates were computed using 1,000 bootstrap replicates.

### Data Availability

All data generated or analyzed during this study are included in this published article and its Supplementary Information files.

## Electronic supplementary material

Supplementary Information
