# Supplementary material for: Molecular diversity of benthic ctenophores (Coeloplanidae)
Source: Sci Rep. 2017 Jul 25;7:6365. doi: 10.1038/s41598-017-06505-4 (PMC5526862; doi:10.1038/s41598-017-06505-4)
Supplement: Supplementary file 1 — Supplementary Information [file 41598_2017_6505_MOESM1_ESM.doc]

**Molecular diversity of benthic ctenophores (Coeloplanidae)**

Ada Alamaru^1^, Bert W. Hoeksema^2^, Sancia E.T. van der Meij^2,3^, Dorothée Huchon^1,4^

^1^Department of Zoology, George S. Wise Faculty of Life Sciences, Tel-Aviv University, Tel-Aviv 69978, Israel.

^2^Naturalis Biodiversity Center, P.O.Box 9517, 2300 RA, Leiden, The Netherlands

^3^Oxford University Museum of Natural History, University of Oxford, Parks Road, Oxford OX1 3PW, United Kingdom

^4^Steinhardt Museum of Natural History, Israel National Center for Biodiversity Studies, Tel-Aviv University, Tel-Aviv 6997801, Israel.

Correspondence and requests for materials should be addressed to: A.A. (e-mail: [alamarua@gmail.com](mailto:alamarua@gmail.com))

**Table S1 – Available sequences of Coeloplanidae in the nucleotide database of NCBI (accessed on October 8^th^ 2016).**

|  | **Species** | **Marker type** | **Marker name** | **Length (bp)** | **Accession** | **Reference** |
| --- | --- | --- | --- | --- | --- | --- |
| *1* | *Coeloplana cf. meteoris* | Nuclear | 18S partial sequence | 1,814 | KJ754157 | Simion et al. 2015^1^ |
| *2* | *Coeloplana cf. meteoris* | Nuclear | ITS1, partial sequence + 5.8S + ITS2 + 28S partial sequence | 713 | KJ754170 | Simion et al. 2015^1^ |
| *3* | *Coeloplana anthostella* | Nuclear | 18S partial sequence | 1,768 | HQ435810 | Song et al. 2011[^2^](#_ENREF_2) |
| *4* | *Coeloplana anthostella* | Mitochondrial | COI, partial CDS | 664 | HQ435811 | Song et al. 2011[^2^](#_ENREF_2) |
| *5* | *Coeloplana anthostella* | Nuclear | ITS1, partial sequence | 271 | HQ435812 | Song et al. 2011[^2^](#_ENREF_2) |
| *6* | *Coeloplana bocki* | Nuclear | 18S, partial sequence | 1,768 | HQ435813 | Song et al. 2011[^2^](#_ENREF_2) |
| *7* | *Coeloplana bocki* | Nuclear | ITS1, partial sequence | 271 | HQ435814 | Song et al. 2011[^2^](#_ENREF_2) |
| *8* | *Coeloplana bocki* | Mitochondrial | COI, partial CDS | 664 | HQ435815 | Song et al. 2011[^2^](#_ENREF_2) |
| *9* | *Coeloplana willeyi* | mRNA | Pax-A | 1,843 | AB239691 | Hoshiyama et al. 2007^2^ |
| *10* | *Coeloplana willeyi* | mRNA | Pax-B | 1,941 | AB239692 | Hoshiyama et al. 2007^2^ |
| *11* | *Coeloplana willeyi* | mRNA | Six-A | 1,307 | AB239699 | Hoshiyama et al. 2007^2^ |
| *12* | *Coeloplana willeyi* | mRNA | Six-B1, partial cds | 1,609 | AB239700 | Hoshiyama et al. 2007^2^ |
| *13* | *Coeloplana willeyi* | mRNA | Six-C | 1,391 | AB239701 | Hoshiyama et al. 2007^2^ |
| *14* | *Coeloplana agniae* | Nuclear | 18S, partial sequence | 1,821 | AF358112 | Collins AG 2002^3^ |
| *15* | *Coeloplana bannwarthi* | Nuclear | 18S + ITS1 + 5.8S + ITS2 + 28S partial sequence | 2,529 | AF293683 | Podar et al. 2001^4^ |
| *16* | *Vallicula multiformis* | Nuclear | 18S + ITS1 + 5.8S + ITS2 + 28S partial sequence | 2,556 | AF293684 | Podar et al. 2001^4^ |

**Table S2** - Genetic markers used in this study: nuclear 18S rDNA (partial sequence), 28S rDNA (partial sequence, C1-D2 domains), the Internal Transcribed Spacers subunit 1 (ITS1) partial sequences and the mitochondrial gene Cytochrome Oxidase subunit 1 (COI) partial sequence.

| Marker | Primer | Sequence (5’🡪3’) | Length | Primer source |
| --- | --- | --- | --- | --- |
| 18S rDNA | 18S | AACCTGGTTGATCCTGCCA | 1800 bp | Borchiellini et al. 2001^5^ |
|  | 18SS | TGAAGG TTCACCTACAGAA |  |  |
| 28S rDNA | ^*^C1_modified_ | ACCCGC**Y**GAAYTTAAGCAT | 780 bp | Szitenberg et al. 2013^6^ |
|  | D2 | TCCGTGTTTCAAGACGGG |  | Chombard et al. 1998^7^ |
| ITS1 | 025 | TAACAAGGTTTCCGTAGGTG | 285 bp | White et al. 1990**^8^** |
|  | 026 | AGCTRCGTGCGTTCTTCATCGA |  |  |
| COI | LCOI490 | GGTCAACAAATCATAAAGATATTGG | 665 bp | Folmer et al. 1994**^9^** |
|  | HCO2198 | TAAACTTCAGGGTGACCAAAAAATCA |  |  |

*Primer C1_modified_ is the original sequence reported by Chombard et al. (1998) but has Y instead of T (highlighted in yellow).

**Table S3** – All samples analyzed in this study versus their accession numbers in NCBI

| **Genus** | **Species** | **Isolate** | **Collected by** | **Collection date** | **Country** | **Lat_Lon** | **Host** | **NCBI accession numbers** | | | |
| --- | --- | --- | --- | --- | --- | --- | --- | --- | --- | --- | --- |
|  |  |  |  |  |  |  |  | 18S | 28S | ITS1 | COI |
| *Coeloplana* | sp.2 | TMP-S4 | S.E.T. van der Meij and B.W. Hoeksema | 18-Sep-12 | Malaysia | 5.96 N 116.03 E | *Sarcophyton* sp. | KT885933 | KT885954 |  | KT885999 |
| *Coeloplana* | *astericola* | TMP-A1 | S.E.T. van der Meij | 18-Sep-12 | Malaysia | 5.96 N 116.03 E | *Echinaster* sp. | KT885934 | KT885953 | KT8855967 | KT885989 |
| *Coeloplana* | sp.3 | TMP-C37 | B.W. Hoeksema | 19-Sep-12 | Malaysia | 5.96 N 116.03 E | *Cycloseris costulata* | KT885935 | KT885956 | KT8855968 | KT885995 |
| *Coeloplana* | sp.2 | TMP-S1 | S.E.T. van der Meij and B.W. Hoeksema | 18-Sep-12 | Malaysia | 5.96 N 116.03 E | *Sarcophyton* sp. |  | KT885955 |  | KT885996 |
| *Coeloplana* | *huchonae* | RS2001-2 | A. Alamaru and E. Brokovich | 6-Sep-12 | Israel | 29.504 N 34.919 E | *Dendronephthya hemprichi* | KT885936 |  |  |  |
| *Coeloplana* | *huchonae* | RS2001-1 | A. Alamaru and E. Brokovich | 6-Sep-12 | Israel | 29.504 N 34.919 E | *Dendronephthya hemprichi* |  | KT885947 |  |  |
| *Coeloplana* | *punctata* | RS2003-2 | A. Alamaru and E. Brokovich | 5-Sep-12 | Israel | 29.504 N 34.919 E | *Sarcophyton glaucum* | KT885937 | KT885948 |  | KT885983 |
| *Coeloplana* | *lineolata* | RS2004-2 | A. Alamaru and E. Brokovich | 27-May-12 | Israel | 29.504 N 34.919 E | *Sarcophyton glaucum* | KT885938 | KT885949 |  | KT885982 |
| *Coeloplana* | *yulianicorum* | RS2002-2 | A. Alamaru and E. Brokovich | 3-Sep-12 | Israel | 29.504 N 34.919 E | *Sarcophyton glaucum* | KT885939 | KT885952 |  |  |
| *Coeloplana* | *fishelsoni* | RS2012-2 | A. Alamaru and E. Brokovich | 6-Sep-12 | Israel | 29.504 N 34.919 E | *Paralemnalia* sp. | KT885940 | KT885957 |  | KT885978 |
| *Coeloplana* | *fishelsoni* | RS2011-2 | A. Alamaru and E. Brokovich | 6-Sep-12 | Israel | 29.504 N 34.919 E | *Xenia umbellata* | KT885941 | KT885950 |  | KT885975 |
| *Coeloplana* | *bannwarthi* | RS2000-2DH | A. Alamaru and E. Brokovich | 27-May-12 | Israel | 29.504 N 34.919 E | *Diadema setosum* | KT885942 |  |  |  |
| *Coeloplana* | *bannwarthi* | RS2000-3 | A. Alamaru and E. Brokovich | 27-May-12 | Israel | 29.504 N 34.919 E | *Diadema setosum* |  | KT885945 |  |  |
| *Coeloplana* | *loyai* | RS2013-2 | A. Alamaru and E. Brokovich | 5-Sep-12 | Israel | 29.504 N 34.919 E | *Herpolitha limax* | KT885943 | KT885951 |  | KT886004 |
| *Vallicula* | *multiformis* | RS2005-2 | A. Alamaru and E. Brokovich | 27-May-12 | Israel | 29.504 N 34.919 E | *Sargassum* sp. | KT885944 |  | KT8855969 | KT886022 |
| *Vallicula* | *multiformis* | RS2005-1 | A. Alamaru and E. Brokovich | 27-May-12 | Israel | 29.504 N 34.919 E | *Sargassum* sp. |  | KT885958 |  |  |
| *Coeloplana* | sp. 1 brown dots | RS2010-1 | A. Alamaru and E. Brokovich | 5-Sep-12 | Israel | 29.504 N 34.919 E | *Sarcophyton glaucum* |  | KT885946 |  | KT885970 |
| *Coeloplana* | *bannwarthi* | RS1 | A. Alamaru and E. Brokovich | 27-May-12 | Israel | 29.504 N 34.919 E | *Diadema setosum* |  |  | KT8855959 |  |
| *Coeloplana* | *lineolata* |  | A. Alamaru and E. Brokovich | 27-May-12 | Israel | 29.504 N 34.919 E | *Sarcophyton glaucum* |  |  | KT8855960 |  |
| *Coeloplana* | *fishelsoni* | RS2011-1 | A. Alamaru and E. Brokovich | 6-Sep-12 | Israel | 29.504 N 34.919 E | *Xenia umbellata* |  |  | KT8855961 | KT885974 |
| *Coeloplana* | *bannwarthi* var. | RS2000-11 | A. Alamaru and E. Brokovich | 27-May-12 | Israel | 29.504 N 34.919 E | *Diadema setosum* |  |  | KT8855962 | KT886018 |
| *Coeloplana* | *fishelsoni* var. | RS2011-3 | A. Alamaru and E. Brokovich | 6-Sep-12 | Israel | 29.504 N 34.919 E | *Xenia umbellata* |  |  | KT8855963 | KT885976 |
| *Coeloplana* | *huchonae* |  | A. Alamaru and E. Brokovich | 6-Sep-12 | Israel | 29.504 N 34.919 E | *Dendronephthya hemprichi* |  |  | KT8855964 |  |
| *Coeloplana* | *yulianicorum* |  | A. Alamaru and E. Brokovich | 3-Sep-12 | Israel | 29.504 N 34.919 E | *Sarcophyton glaucum* |  |  | KT8855965 |  |
| *Coeloplana* | *punctata* |  | A. Alamaru and E. Brokovich | 5-Sep-12 | Israel | 29.504 N 34.919 E | *Sarcophyton glaucum* |  |  | KT8855966 |  |
| *Coeloplana* | sp. 1 brown dots | RS2010-2 | A. Alamaru and E. Brokovich | 5-Sep-12 | Israel | 29.504 N 34.919 E | *Sarcophyton glaucum* |  |  |  | KT885971 |
| *Coeloplana* | sp. 1 brown dots | RS2010-3 | A. Alamaru and E. Brokovich | 5-Sep-12 | Israel | 29.504 N 34.919 E | *Sarcophyton glaucum* |  |  |  | KT885972 |
| *Coeloplana* |  | RSxxx | A. Alamaru and E. Brokovich | 5-Sep-12 | Israel | 29.504 N 34.919 E | *Sarcophyton glaucum* |  |  |  | KT885973 |
| *Coeloplana* | *fishelsoni* | RS2012-1 | A. Alamaru and E. Brokovich | 6-Sep-12 | Israel | 29.504 N 34.919 E | *Paralemnalia* sp. |  |  |  | KT885977 |
| *Coeloplana* | *fishelsoni* | RS2012-3 | A. Alamaru and E. Brokovich | 6-Sep-12 | Israel | 29.504 N 34.919 E | *Paralemnalia* sp. |  |  |  | KT885979 |
| *Coeloplana* | *lineolata* | RS2004-4 | A. Alamaru and E. Brokovich | 27-May-12 | Israel | 29.504 N 34.919 E | *Sarcophyton glaucum* |  |  |  | KT885980 |
| *Coeloplana* | *lineolata* | RS2004-35 | A. Alamaru and E. Brokovich | 27-May-12 | Israel | 29.504 N 34.919 E | *Sarcophyton glaucum* |  |  |  | KT885981 |
| *Coeloplana* | *punctata* | RS2003-35 | A. Alamaru and E. Brokovich | 5-Sep-12 | Israel | 29.504 N 34.919 E | *Sarcophyton glaucum* |  |  |  | KT885984 |
| *Coeloplana* | *punctata* | RS2003-45 | A. Alamaru and E. Brokovich | 5-Sep-12 | Israel | 29.504 N 34.919 E | *Sarcophyton glaucum* |  |  |  | KT885985 |
| *Coeloplana* | *yulianicorum* | RS2002-6 | A. Alamaru and E. Brokovich | 3-Sep-12 | Israel | 29.504 N 34.919 E | *Sarcophyton glaucum* |  |  |  | KT885986 |
| *Coeloplana* | *yulianicorum* | RS2002-65 | A. Alamaru and E. Brokovich | 3-Sep-12 | Israel | 29.504 N 34.919 E | *Sarcophyton glaucum* |  |  |  | KT885987 |
| *Coeloplana* | *yulianicorum* | RS2002-xxx | A. Alamaru and E. Brokovich | 3-Sep-12 | Israel | 29.504 N 34.919 E | *Sarcophyton glaucum* |  |  |  | KT885988 |
| *Coeloplana* | *astericola* | TMP-A2 | S.E.T. van der Meij | 18-Sep-12 | Malaysia | 5.96 N 116.03 E | *Echinaster* sp. |  |  |  | KT885990 |
| *Coeloplana* | *astericola* | TMP-A3 | S.E.T. van der Meij | 18-Sep-12 | Malaysia | 5.96 N 116.03 E | *Echinaster* sp. |  |  |  | KT885991 |
| *Coeloplana* | *astericola* | TMP-A4 | S.E.T. van der Meij | 18-Sep-12 | Malaysia | 5.96 N 116.03 E | *Echinaster* sp. |  |  |  | KT885992 |
| *Coeloplana* | *astericola* | TMP-A5 | S.E.T. van der Meij | 18-Sep-12 | Malaysia | 5.96 N 116.03 E | *Echinaster* sp. |  |  |  | KT885993 |
| *Coeloplana* | green dots | RS2003-xxx | A. Alamaru and E. Brokovich | 5-Sep-12 | Israel | 29.504 N 34.919 E | *Sarcophyton glaucum* |  |  |  | KT885994 |
| *Coeloplana* | sp.2 | TMPS-2 | S.E.T. van der Meij and B.W. Hoeksema | 18-Sep-12 | Malaysia | 5.96 N 116.03 E | *Sarcophyton* sp. |  |  |  | KT885997 |
| *Coeloplana* | sp.2 | TMPS-3 | S.E.T. van der Meij and B.W. Hoeksema | 18-Sep-12 | Malaysia | 5.96 N 116.03 E | *Sarcophyton* sp. |  |  |  | KT885998 |
| *Coeloplana* | *lineolata* | RS2004-3 | A. Alamaru and E. Brokovich | 27-May-12 | Israel | 29.504 N 34.919 E | *Sarcophyton glaucum* |  |  |  | KT886000 |
| *Coeloplana* | *punctata* | RS2003-1 | A. Alamaru and E. Brokovich | 5-Sep-12 | Israel | 29.504 N 34.919 E | *Sarcophyton glaucum* |  |  |  | KT886001 |
| *Coeloplana* | *punctata* | RS2003-4 | A. Alamaru and E. Brokovich | 5-Sep-12 | Israel | 29.504 N 34.919 E | *Sarcophyton glaucum* |  |  |  | KT886002 |
| *Coeloplana* | *loyai* | RS2013-1 | A. Alamaru and E. Brokovich | 5-Sep-12 | Israel | 29.504 N 34.919 E | *Herpolitha limax* |  |  |  | KT886003 |
| *Coeloplana* | *loyai* | RS2013-9 | A. Alamaru and E. Brokovich | 5-Sep-12 | Israel | 29.504 N 34.919 E | *Herpolitha limax* |  |  |  | KT886005 |
| *Coeloplana* | *loyai* | RS2013-10 | A. Alamaru and E. Brokovich | 5-Sep-12 | Israel | 29.504 N 34.919 E | *Herpolitha limax* |  |  |  | KT886006 |
| *Coeloplana* | *bannwarthi* | RS2000-6 | A. Alamaru and E. Brokovich | 27-May-12 | Israel | 29.504 N 34.919 E | *Diadema setosum* |  |  |  | KT886007 |
| *Coeloplana* | *bannwarthi* | RS2000-12 | A. Alamaru and E. Brokovich | 27-May-12 | Israel | 29.504 N 34.919 E | *Diadema setosum* |  |  |  | KT886008 |
| *Coeloplana* | *bannwarthi* | RS2000-14 | A. Alamaru and E. Brokovich | 27-May-12 | Israel | 29.504 N 34.919 E | *Diadema setosum* |  |  |  | KT886009 |
| *Coeloplana* | *bannwarthi* |  | A. Alamaru and E. Brokovich | 27-May-12 | Israel | 29.504 N 34.919 E | *Diadema setosum* |  |  |  | KT886010 |
| *Coeloplana* | *bannwarthi* | RS2000-17 | A. Alamaru and E. Brokovich | 27-May-12 | Israel | 29.504 N 34.919 E | *Diadema setosum* |  |  |  | KT886011 |
| *Coeloplana* | *bannwarthi* | RS2000-18 | A. Alamaru and E. Brokovich | 27-May-12 | Israel | 29.504 N 34.919 E | *Diadema setosum* |  |  |  | KT886012 |
| *Coeloplana* | *huchonae* | RS2001-3 | A. Alamaru and E. Brokovich | 6-Sep-12 | Israel | 29.504 N 34.919 E | *Dendronephthya hemprichi* |  |  |  | KT886013 |
| *Coeloplana* | *huchonae* | RS2001-4 | A. Alamaru and E. Brokovich | 6-Sep-12 | Israel | 29.504 N 34.919 E | *Dendronephthya hemprichi* |  |  |  | KT886014 |
| *Coeloplana* | *huchonae* | RS2001-7 | A. Alamaru and E. Brokovich | 6-Sep-12 | Israel | 29.504 N 34.919 E | *Dendronephthya hemprichi* |  |  |  | KT886015 |
| *Coeloplana* | *huchonae* | RS2001-5 | A. Alamaru and E. Brokovich | 6-Sep-12 | Israel | 29.504 N 34.919 E | *Dendronephthya hemprichi* |  |  |  | KT886016 |
| *Coeloplana* | *huchonae* | RS2001-xxx | A. Alamaru and E. Brokovich | 6-Sep-12 | Israel | 29.504 N 34.919 E | *Dendronephthya hemprichi* |  |  |  | KT886017 |
| *Coeloplana* | *yulianicorum* | RS2002-4 | A. Alamaru and E. Brokovich | 3-Sep-12 | Israel | 29.504 N 34.919 E | *Sarcophyton glaucum* |  |  |  | KT886019 |
| *Coeloplana* | *yulianicorum* | RS2002-7 | A. Alamaru and E. Brokovich | 3-Sep-12 | Israel | 29.504 N 34.919 E | *Sarcophyton glaucum* |  |  |  | KT886020 |
| *Coeloplana* | *loyai* | RS2013-3 | A. Alamaru and E. Brokovich | 5-Sep-12 | Israel | 29.504 N 34.919 E | *Herpolitha limax* |  |  |  | KT886021 |
| *Vallicula* | *multiformis* | RS2005-5 | A. Alamaru and E. Brokovich | 27-May-12 | Israel | 29.504 N 34.919 E | *Sargassum* sp. |  |  |  | KT886023 |
| *Vallicula* | *multiformis* | RS2005-12 | A. Alamaru and E. Brokovich | 27-May-12 | Israel | 29.504 N 34.919 E | *Sargassum* sp. |  |  |  | KT886024 |
| *Vallicula* | *multiformis* | RS2007-1 green morph | A. Alamaru and E. Brokovich | 27-May-12 | Israel | 29.504 N 34.919 E | *Sargassum* sp. |  |  |  | KT886025 |
| *Vallicula* | *multiformis* | RS2008-2 | A. Alamaru and E. Brokovich | 27-May-12 | Israel | 29.504 N 34.919 E | *Pearsonothuria graeffei* |  |  |  | KT886026 |
| *Vallicula* | *multiformis* | RSxxx | A. Alamaru and E. Brokovich | 27-May-12 | Israel | 29.504 N 34.919 E | *Sargassum* sp. |  |  |  | KT886027 |

**SUPPLEMENTARY REFERENCES**

1 Simion, P., Bekkouche, N., Jager, M., Quéinnec, E. & Manuel, M. Exploring the potential of small RNA subunit and ITS sequences for resolving phylogenetic relationships within the phylum Ctenophora. *Zoology* **118**, 102-114, doi:http://dx.doi.org/10.1016/j.zool.2014.06.004 (2015).

2 Hoshiyama, D., Iwabe, N. & Miyata, T. Evolution of the gene families forming the Pax/Six regulatory network: Isolation of genes from primitive animals and molecular phylogenetic analyses. *FEBS Letters* **581**, 1639-1643, doi:http://doi.org/10.1016/j.febslet.2007.03.027 (2007).

3 Collins, A. G. Phylogeny of Medusozoa and the evolution of cnidarian life cycles. *J. Evol. Biol.* **15**, 418-432, doi:10.1046/j.1420-9101.2002.00403.x (2002).

4 Podar, M., Haddock, H. D., Sogin, M. L. & Harbison, G. R. A molecular phylogenetic framework for the phylum Ctenophora using 18S rRNA genes. *Mol. Phylogenet. Evol.* **21**, 218-230 doi: <https://doi.org/10.1006/mpev.2001.1036> (2001).

5 Borchiellini, C. *et al.* Sponge phylogeny and the origin of Metazoa. *J. Evol.Biol.* **14**, 171-179 doi:10.1046/j.1420-9101.2001.00244.x (2001).

6 Szitenberg, A. *et al.* Phylogeny of Tetillidae (Porifera, Demospongiae, Spirophorida) based on three molecular markers. *Mol. Phylogenet. Evol.* **67**, 509-519 doi:10.1016/j.ympev.2013.02.018 (2013).

7 Chombard, C., Boury-Esnault, N. & Tillier, S. Reassessment of homology of morphological characters in Tetractinellid sponges based on molecular data. *System. Biol.* **47**, 351-366 doi: <https://doi.org/10.1080/106351598260761> (1998).

8 White, T. J., Bruns, T., Lee, S. & Taylor, J. W. in *PCR protocols: a guide to methods and applications* (eds M.A. Innis, D.J. Gelfand, J.J. Sninsky, & T.J. White) 315-322 (Academic Press Inc., 1990).

9 Folmer, O., Black, M., Hoeh, W., Lutz, R. & Vrijenhoek, R. DNA primers for amplification of mitochondrial cytochrome c oxidase subunit 1 from diverse metazoan invertebrates. *Mol. Mar. Biol. Biotech.* **3**, 294-297 (1994).

**MOLECULAR SEQUENCES**

**18S nucleotide alignment. DNA sequence alignment, in Nexus format, used to reconstruct the phylogenetic tree presented in Figure 1**

#NEXUS

Begin data;

Dimensions ntax=18 nchar=1746;

Format datatype=DNA gap=- missing=? matchchar=.;

Matrix

KJ754157_Coeloplana_meteoris GCTTGTCTCAAAGATTAAGCCATGCATGTCTAAGTATAAACTTTTATACTGTGAAACTGCGAATGGCTCATTAAATCAGTTATCGTCTATTTGATTGTGCCCCTTACTACATGGATAACCGTAGTAATTCTAGAGCTAATACATGCGAAAAGTCCCGACTTACGGAAGGGATGTATTTATTAGATTAAAAACCAACGCGTTTCTCCAGAGACGCTCCAAGGTGATTCATAATAACTGTTCGAATCGCACGGCCTCCGCGCCAGCGATGTTTCATTCGAGTTTCTGCCCTATCAACTTTCGATGGTAAGGTATTGGCTTACCATGGTTACAACGGGTAACGGAGAATTAGGGTTCGATTCCGGAGAGGGAGCCCGAGAAACGGCTACCACATCCAAGGAAGGCAGCAGGCGCGCAAATTACCCAATCCCGACTCGGGGAGGTAGTGACAATAAATAACGTTGCAGGCGCCAACGGCTTCTGCAGTCGGAATGAGTACAATATAACACCCTTAACGAGGAACAATTGGAGGGCAAGTCTGGTGCCAGCAGCCGCGGTAATTCCAGCTCCAATAGCGTATATTAAAGTTGTTGCAGTTAAAAAGCTCGTAGTTGGACTTCGGAACTGGCCGATTGGTCCGCCCTT--CGGGTTGAGTACTGATCGGTCTGTTCTTCTTCGCGAAGACTGCGTGTGCCCTTAACTGGGTGTGCGTGGGATTCACGACGTTTACTTTGAAAAAATTAGAGTGTTCAAAGCAGGCAATCGCTTGAATATCTCAGCATGGAATAATAGAATAGGACTTTGGTCTTATTTTGTTGGTTTCCGAGACCGAAGTAATGATTAATAGGGACAGTTGGGGGCATTCGTATTTCATTGTCAGAGGTGAAATTCTTGGATTTATGAAAGACGAACTTCTGCGAAAGCATTTGCCAAGGATGTTTTCATTAATCAAGAACGAAAGTTGGAGGCTCGAAGACGATCAGATACCGTCCTAGTTCCAACCATAAACGATGCCGTCTGCGGATCGGCGGATGTTCACTTAAAGCACCGTCGGCACGCTATGAGAAATCAAAGACTTCGGGTTCCGGGGGGAGTATGTTCGCAAGAATGAAACTTAAAGGAATTGACGGAAGGGCACCACCAGGAGTGGAACCTGCGGTTTAATTTGACTCAACACGGGAAAACTCACCAGGTCCAGACATAGGAAGGATTGACAGATTGATAGCTCTTTCTTGATTCTATGGGTGGTGGTGCATGGCCGTTCTTAGTTGGTGGAGTGATTTGTCTGGTTAATTCCGTTAACGAACGAGACCTTAACCTGCTAAATAGTGACACAGTTCTTATGAAATGTGGTTCACTTCTTAGAGGGACTATCGGATTGAAGCCGATGGAAGTTTGAGGCAATAACAGGTCTGTGATGCCCTTAGATGTTCTGGGCCACACGCGCGTTACACTGATGAAGCCAGCGAGTATATCGCCTACACCGGAAGGTGCGGGTAATCTTGTGAAACTTCATCGTGCTGGGGATAGACCATTGCAATTATTGGTCTTGAACGAGGAATTCCTAGTAAGCACGAGTCATCAACTCGTGCTGATTACGTCCCTGCCCTTTGTACACACCGCCCGTCGCTACTACCGATTGAATGGTTTAGTGAGATCTCGGGATTGGCGACGCCATGCCGCAAGGCGCGGCGCCGCCGAGAACTTGCTCAAACTTGATCATTTAGAGGAAGTAAAAGTCGTAACA

KT885933_Coeloplana_sp_2_Malaysia GCTTGTCTCAAAGATTAAGCCATGCATGTCTAAGTATAAACTTTTATACTGTGAAACTGCGAATGGCTCATTAAATCAGTTATCGTCTATTTGATTGTGCCCCTTACTACATGGATAACCGTAGTAATTCTAGAGCTAATACATGCGAAAAGTCCCGACTTACGGAAGGGATGTATTTATTAGATTAAAAACCAACGCGTTTCTCCAGAGACGCTCCAAGGTGATTCATAATAACTGTTCGAATCGCACGGCCTCCGCGCCAGCGATGTTTCATTCGAGTTTCTGCCCTATCAACTTTCGATGGTAAGGTATTGGCTTACCATGGTTACAACGGGTAACGGAGAATTAGGGTTCGATTCCGGAGAGGGAGCCCGAGAAACGGCTACCACATCCAAGGAAGGCAGCAGGCGCGCAAATTACCCAATCCCGACTCGGGGAGGTAGTGACAATAAATAACGTTGCAGGCGCCAACGGCTTCTGCAGTCGGAATGAGTACAATATAACACCCTTAACGAGGAACAATTGGAGGGCAAGTCTGGTGCCAGCAGCCGCGGTAATTCCAGCTCCAATAGCGTATATTAAAGTTGTTGCAGTTAAAAAGCTCGTAGTTGGACTTCGGAACTGGCCGATTGGTCCGCCCTT--CGGGTTGAGTACTGATCGGTCTGTTCTTCTTCGCGAAGACTGCGTGTGCCCTTAACTGGGTGTGCGTGGGATTCACGACGTTTACTTTGAAAAAATTAGAGTGTTCAAAGCAGGCAATCGCTTGAATATCTCAGCATGGAATAATAGAATAGGACTTTGGTCTTATTTTGTTGGTTTCCGAGACCGAAGTAATGATTAATAGGGACAGTTGGGGGCATTCGTATTTCATTGTCAGAGGTGAAATTCTTGGATTTATGAAAGACGAACTTCTGCGAAAGCATTTGCCAAGGATGTTTTCATTAATCAAGAACGAAAGTTGGAGGCTCGAAGACGATCAGATACCGTCCTAGTTCCAACCATAAACGATGCCGTCTGCGGATCGGCGGATGTTCACTTAAAGCACCGTCGGCACGCTATGAGAAATCAAAGACTTCGGGTTCCGGGGGGAGTATGTTCGCAAGAATGAAACTTAAAGGAATTGACGGAAGGGCACCACCAGGAGTGGAACCTGCGGTTTAATTTGACTCAACACGGGAAAACTCACCAGGTCCAGACATAGGAAGGATTGACAGATTGATAGCTCTTTCTTGATTCTATGGGTGGTGGTGCATGGCCGTTCTTAGTTGGTGGAGTGATTTGTCTGGTTAATTCCGTTAACGAACGAGACCTTAACCTGCTAAATAGTGACACAGTTCTTATGAAATGTGGTTCACTTCTTAGAGGGACTATCGGATTGAAGCCGATGGAAGTTTGAGGCAATAACAGGTCTGTGATGCCCTTAGATGTTCTGGGCCACACGCGCGTTACACTGATGAAGCCAGCGAGTATATCGCCTACACCGGAAGGTGCGGGTAATCTTGTGAAACTTCATCGTGCTGGGGATAGACCATTGCAATTATTGGTCTTGAACGAGGAATTCCTAGTAAGCACGAGTCATCAACTCGTGCTGATTACGTCCCTGCCCTTTGTACACACCGCCCGTCGCTACTACCGATTGAATGGTTTAGTGAGATCTCGGGATTGGCGACGCCATGCCGCAAGGCGCGGCGCCGCCGAGAACTTGCTCAAACTTGATCATTTAGAGGAAGTAAAAGTCGTAACA

KT885934_Coeloplana_astricola_Malaysia GCTTGTCTCAAAGATTAAGCCATGCATGTCTAAGTATAAACTTTTATACTGTGAAACTGCGAATGGCTCATTAAATCAGTTATCGTCTATTTGATTGTGCCCCTTACTACATGGATAACCGTAGTAATTCTAGAGCTAATACATGCGAAAAGTCCCGACTTACGGAAGGGATGTATTTATTAGATTAAAAACCAACGCGTTTCTCCAGAGACGCTCCAAGGTGATTCATAATAACTGTTCGAATCGCACGGCCTCCGCGCCAGCGATGTTTCATTCGAGTTTCTGCCCTATCAACTTTCGATGGTAAGGTATTGGCTTACCATGGTTACAACGGGTAACGGAGAATTAGGGTTCGATTCCGGAGAGGGAGCCCGAGAAACGGCTACCACATCCAAGGAAGGCAGCAGGCGCGCAAATTACCCAATCCCGACTCGGGGAGGTAGTGACAATAAATAACGTTGCAGGCGCCAACGGCTTCTGCAGTCGGAATGAGTACAATATAACACCCTTAACGAGGAACAATTGGAGGGCAAGTCTGGTGCCAGCAGCCGCGGTAATTCCAGCTCCAATAGCGTATATTAAAGTTGTTGCAGTTAAAAAGCTCGTAGTTGGACTTCGGAACTGGCCGATTGGTCCGCCCTT--CGGGTTGAGTACTGATCGGTCTGTTCTTCTTCGCGAAGACTGCGTGTGCCCTTAACTGGGTGTGCGTGGGATTCACGACGTTTACTTTGAAAAAATTAGAGTGTTCAAAGCAGGCAATCGCTTGAATATCTCAGCATGGAATAATAGAATAGGACTTTGGTCTTATTTTGTTGGTTTCCGAGACCGAAGTAATGATTAATAGGGACAGTTGGGGGCATTCGTATTTCATTGTCAGAGGTGAAATTCTTGGATTTATGAAAGACGAACTTCTGCGAAAGCATTTGCCAAGGATGTTTTCATTAATCAAGAACGAAAGTTGGAGGCTCGAAGACGATCAGATACCGTCCTAGTTCCAACCATAAACGATGCCGTCTGCGGATCGGCGGATGTTCACTTAAAGCACCGTCGGCACGCTATGAGAAATCAAAGACTTCGGGTTCCGGGGGGAGTATGTTCGCAAGAATGAAACTTAAAGGAATTGACGGAAGGGCACCACCAGGAGTGGAACCTGCGGTTTAATTTGACTCAACACGGGAAAACTCACCAGGTCCAGACATAGGAAGGATTGACAGATTGATAGCTCTTTCTTGATTCTATGGGTGGTGGTGCATGGCCGTTCTTAGTTGGTGGAGTGATTTGTCTGGTTAATTCCGTTAACGAACGAGACCTTAACCTGCTAAATAGTGACACAGTTCTTATGAAATGTGGTTCACTTCTTAGAGGGACTATCGGATTGAAGCCGATGGAAGTTTGAGGCAATAACAGGTCTGTGATGCCCTTAGATGTTCTGGGCCACACGCGCGTTACACTGATGAAGCCAGCGAGTATATCGCCTACACCGGAAGGTGCGGGTAATCTTGTGAAACTTCATCGTGCTGGGGATAGACCATTGCAATTATTGGTCTTGAACGAGGAATTCCTAGTAAGCACGAGTCATCAACTCGTGCTGATTACGTCCCTGCCCTTTGTACACACCGCCCGTCGCTACTACCGATTGAATGGTTTAGTGAGATCTCGGGATTGGCGACGCCATGCCGCAAGGCGCGGCGCCGCCGAGAACTTGCTCAAACTTGATCATTTAGAGGAAGTAAAAGTCGTAACA

KT885935_Coeloplana_sp_3_Malaysia GCTTGTCTCAAAGATTAAGCCATGCATGTCTAAGTATAAACTTTTATACTGTGAAACTGCGAATGGCTCATTAAATCAGTTATCGTCTATTTGATTGTGCCCCTTACTACATGGATAACCGTAGTAATTCTAGAGCTAATACATGCGAAAAGTCCCGACTTACGGAAGGGATGTATTTATTAGATTAAAAACCAACGCGTTTCTCCAGAGACGCTCCAAGGTGATTCATAATAACTGTTCGAATCGCACGGCCTCCGCGCCAGCGATGTTTCATTCGAGTTTCTGCCCTATCAACTTTCGATGGTAAGGTATTGGCTTACCATGGTTACAACGGGTAACGGAGAATTAGGGTTCGATTCCGGAGAGGGAGCCCGAGAAACGGCTACCACATCCAAGGAAGGCAGCAGGCGCGCAAATTACCCAATCCCGACTCGGGGAGGTAGTGACAATAAATAACGTTGCAGGCGCCAACGGCTTCTGCAGTCGGAATGAGTACAATATAACACCCTTAACGAGGAACAATTGGAGGGCAAGTCTGGTGCCAGCAGCCGCGGTAATTCCAGCTCCAATAGCGTATATTAAAGTTGTTGCAGTTAAAAAGCTCGTAGTTGGACTTCGGAACTGGCCGATTGGTCCGCCCTT--CGGGTTGAGTACTGATCGGTCTGTTCTTCTTCGCGAAGACTGCGTGTGCCCTTAACTGGGTGTGCGTGGGATTCACGACGTTTACTTTGAAAAAATTAGAGTGTTCAAAGCAGGCAATCGCTTGAATATCTCAGCATGGAATAATAGAATAGGACTTTGGTCTTATTTTGTTGGTTTCCGAGACCGAAGTAATGATTAATAGGGACAGTTGGGGGCATTCGTATTTCATTGTCAGAGGTGAAATTCTTGGATTTATGAAAGACGAACTTCTGCGAAAGCATTTGCCAAGGATGTTTTCATTAATCAAGAACGAAAGTTGGAGGCTCGAAGACGATCAGATACCGTCCTAGTTCCAACCATAAACGATGCCGTCTGCGGATCGGCGGATGTTCACTTAAAGCACCGTCGGCACGCTATGAGAAATCAAAGACTTCGGGTTCCGGGGGGAGTATGTTCGCAAGAATGAAACTTAAAGGAATTGACGGAAGGGCACCACCAGGAGTGGAACCTGCGGTTTAATTTGACTCAACACGGGAAAACTCACCAGGTCCAGACATAGGAAGGATTGACAGATTGATAGCTCTTTCTTGATTCTATGGGTGGTGGTGCATGGCCGTTCTTAGTTGGTGGAGTGATTTGTCTGGTTAATTCCGTTAACGAACGAGACCTTAACCTGCTAAATAGTGACACAGTTCTTATGAAATGTGGTTCACTTCTTAGAGGGACTATCGGATTGAAGCCGATGGAAGTTTGAGGCAATAACAGGTCTGTGATGCCCTTAGATGTTCTGGGCCACACGCGCGTTACACTGATGAAGCCAGCGAGTATATCGCCTACACCGGAAGGTGCGGGTAATCTTGTGAAACTTCATCGTGCTGGGGATAGACCATTGCAATTATTGGTCTTGAACGAGGAATTCCTAGTAAGCACGAGTCATCAACTCGTGCTGATTACGTCCCTGCCCTTTGTACACACCGCCCGTCGCTACTACCGATTGAATGGTTTAGTGAGATCTCGGGATTGGCGACGCCATGCCGCAAGGCGCGGCGCCGCCGAGAACTTGCTCAAACTTGATCATTTAGAGGAAGTAAAAGTCGTAACA

KT885936_Coeloplana_huchonae_Red_Sea GCTTGTCTCAAAGATTAAGCCATGCATGTCTAAGTATAAACTTTTATACTGTGAAACTGCGAATGGCTCATTAAATCAGTTATCGTCTATTTGATTGTGCCCCTTACTACATGGATAACCGTAGTAATTCTAGAGCTAATACATGCGAAAAGTCCCGACTTACGGAAGGGATGTATTTATTAGATTAAAAACCAACGCGTTTCTCCAGAGACGCTCCAAGGTGATTCATAATAACTGTTCGAATCGCACGGCCTCCGCGCCAGCGATGTTTCATTCGAGTTTCTGCCCTATCAACTTTCGATGGTAAGGTATTGGCTTACCATGGTTACAACGGGTAACGGAGAATTAGGGTTCGATTCCGGAGAGGGAGCCCGAGAAACGGCTACCACATCCAAGGAAGGCAGCAGGCGCGCAAATTACCCAATCCCGACTCGGGGAGGTAGTGACAATAAATAACGTTGCAGGCGCCAACGGCTTCTGCAGTCGGAATGAGTACAATATAACACCCTTAACGAGGAACAATTGGAGGGCAAGTCTGGTGCCAGCAGCCGCGGTAATTCCAGCTCCAATAGCGTATATTAAAGTTGTTGCAGTTAAAAAGCTCGTAGTTGGACTTCGGAACTGGCCGATTGGTCCGCCCTC--CGGGTTGAGTACTGATCGGTCTGTTCTTCTTCGCGAAGACTGCGTGTGCCCTTAACTGGGTGTGCGTGGGATTCACGACGTTTACTTTGAAAAAATTAGAGTGTTCAAAGCAGGCAATCGCTTGAATATCTCAGCATGGAATAATAGAATAGGACTTTGGTCTTATTTTGTTGGTTTCCGAGACCGAAGTAATGATTAATAGGGACAGTTGGGGGCATTCGTATTTCATTGTCAGAGGTGAAATTCTTGGATTTATGAAAGACGAACTTCTGCGAAAGCATTTGCCAAGGATGTTTTCATTAATCAAGAACGAAAGTTGGAGGCTCGAAGACGATCAGATACCGTCCTAGTTCCAACCATAAACGATGCCGTCTGCGGATCGGCGGATGTTCACTTAAAGCACCGTCGGCACGCTATGAGAAATCAAAGACTTCGGGTTCCGGGGGGAGTATGTTCGCAAGAATGAAACTTAAAGGAATTGACGGAAGGGCACCACCAGGAGTGGAACCTGCGGTTTAATTTGACTCAACACGGGAAAACTCACCAGGTCCAGACATAGGAAGGATTGACAGATTGATAGCTCTTTCTTGATTCTATGGGTGGTGGTGCATGGCCGTTCTTAGTTGGTGGAGTGATTTGTCTGGTTAATTCCGTTAACGAACGAGACCTTAACCTGCTAAATAGTGACACAGTTCTTATGAAATGTGGTTCACTTCTTAGAGGGACTATCGGATTGAAGCCGATGGAAGTTTGAGGCAATAACAGGTCTGTGATGCCCTTAGATGTTCTGGGCCACACGCGCGTTACACTGATGAAGCCAGCGAGTATATCGCCTACACCGGAAGGTGCGGGTAATCTTGTGAAACTTCATCGTGCTGGGGATAGACCATTGCAATTATTGGTCTTGAACGAGGAATTCCTAGTAAGCACGAGTCATCAACTCGTGCTGATTACGTCCCTGCCCTTTGTACACACCGCCCGTCGCTACTACCGATTGAATGGTTTAGTGAGATCTCGGGATTGGCGACGCCATGCCGCAAGGCGCGGCGCCGCCGAGAACTTGCTCAAACTTGATCATTTAGAGGAAGTAAAAGTCGTAACA

KT885937_Coeloplana_punctata_Red_Sea GCTTGTCTCAAAGATTAAGCCATGCATGTCTAAGTATAAACTTTTATACTGTGAAACTGCGAATGGCTCATTAAATCAGTTATCGTCTATTTGATTGTGCCCCTTACTACATGGATAACCGTAGTAATTCTAGAGCTAATACATGCGAAAAGTCCCGACTTACGGAAGGGATGTATTTATTAGATTAAAAACCAACGCGTTTCTCCAGAGACGCTCCAAGGTGATTCATAATAACTGTTCGAATCGCACGGCCTCCGCGCCAGCGATGTTTCATTCGAGTTTCTGCCCTATCAACTTTCGATGGTAAGGTATTGGCTTACCATGGTTACAACGGGTAACGGAGAATTAGGGTTCGATTCCGGAGAGGGAGCCCGAGAAACGGCTACCACATCCAAGGAAGGCAGCAGGCGCGCAAATTACCCAATCCCGACTCGGGGAGGTAGTGACAATAAATAACGTTGCAGGCGCCAACGGCTTCTGCAGTCGGAATGAGTACAATATAACACCCTTAACGAGGAACAATTGGAGGGCAAGTCTGGTGCCAGCAGCCGCGGTAATTCCAGCTCCAATAGCGTATATTAAAGTTGTTGCAGTTAAAAAGCTCGTAGTTGGACTTCGGAACTGGCCGATTGGTCCGCCCTC--CGGGTTGAGTACTGATCGGTCTGTTCTTCTTCGCGAAGACTGCGTGTGCCCTTAACTGGGTGTGCGTGGGATTCACGACGTTTACTTTGAAAAAATTAGAGTGTTCAAAGCAGGCAATCGCTTGAATATCTCAGCATGGAATAATAGAATAGGACTTTGGTCTTATTTTGTTGGTTTCCGAGACCGAAGTAATGATTAATAGGGACAGTTGGGGGCATTCGTATTTCATTGTCAGAGGTGAAATTCTTGGATTTATGAAAGACGAACTTCTGCGAAAGCATTTGCCAAGGATGTTTTCATTAATCAAGAACGAAAGTTGGAGGCTCGAAGACGATCAGATACCGTCCTAGTTCCAACCATAAACGATGCCGTCTGCGGATCGGCGGATGTTCACTTAAAGCACCGTCGGCACGCTATGAGAAATCAAAGACTTCGGGTTCCGGGGGGAGTATGTTCGCAAGAATGAAACTTAAAGGAATTGACGGAAGGGCACCACCAGGAGTGGAACCTGCGGTTTAATTTGACTCAACACGGGAAAACTCACCAGGTCCAGACATAGGAAGGATTGACAGATTGATAGCTCTTTCTTGATTCTATGGGTGGTGGTGCATGGCCGTTCTTAGTTGGTGGAGTGATTTGTCTGGTTAATTCCGTTAACGAACGAGACCTTAACCTGCTAAATAGTGACACAGTTCTTATGAAATGTGGTTCACTTCTTAGAGGGACTATCGGATTGAAGCCGATGGAAGTTTGAGGCAATAACAGGTCTGTGATGCCCTTAGATGTTCTGGGCCACACGCGCGTTACACTGATGAAGCCAGCGAGTATATCGCCTACACCGGAAGGTGCGGGTAATCTTGTGAAACTTCATCGTGCTGGGGATAGACCATTGCAATTATTGGTCTTGAACGAGGAATTCCTAGTAAGCACGAGTCATCAACTCGTGCTGATTACGTCCCTGCCCTTTGTACACACCGCCCGTCGCTACTACCGATTGAATGGTTTAGTGAGATCTCGGGATTGGCGACGCCATGCCGCAAGGCGCGGCGCCGCCGAGAACTTGCTCAAACTTGATCATTTAGAGGAAGTAAAAGTCGTAACA

HQ435810_Coeloplana_anthostella GCTTGTCTCAAAGATTAAGCCATGCATGTCTAAGTATAAACTTTTATACTGTGAAACTGCGAATGGCTCATTAAATCAGTTATCGTCTATTTGATTGTGCCCCTTACTACATGGATAACCGTAGTAATTCTAGAGCTAATACATGCGAAAAGTCCCGACTTACGGAAGGGATGTATTTATTAGATTAAAAACCAACGCGTTTCTCCAGAGACGCTCCAAGGTGATTCATAATAACTGTTCGAATCGCACGGCCTCCGCGCCAGCGATGTTTCATTCGAGTTTCTGCCCTATCAACTTTCGATGGTAAGGTATTGGCTTACCATGGTTACAACGGGTAACGGAGAATTAGGGTTCGATTCCGGAGAGGGAGCCCGAGAAACGGCTACCACATCCAAGGAAGGCAGCAGGCGCGCAAATTACCCAATCCCGACTCGGGGAGGTAGTGACAATAAATAACGTTGCAGGCGCCAACGGCTTCTGCAGTCGGAATGAGTACAATATAACACCCTTAACGAGGAACAATTGGAGGGCAAGTCTGGTGCCAGCAGCCGCGGTAATTCCAGCTCCAATAGCGTATATTAAAGTTGTTGCAGTTAAAAAGCTCGTAGTTGGACTTCGGAACTGGCCGATTGGTCCGCCCTC--CGGGTTGAGTACTGATCGGTCTGTTCTTCTTCGCGAAGACTGCGTGTGCCCTTAACTGGGTGTGCGTGGGATTCACGACGTTTACTTTGAAAAAATTAGAGTGTTCAAAGCAGGCAATCGCTTGAATATCTCAGCATGGAATAATAGAATAGGACTTTGGTCTTATTTTGTTGGTTTCCGAGACCGAAGTAATGATTAATAGGGACAGTTGGGGGCATTCGTATTTCATTGTCAGAGGTGAAATTCTTGGATTTATGAAAGACGAACTTCTGCGAAAGCATTTGCCAAGGATGTTTTCATTAATCAAGAACGAAAGTTGGAGGCTCGAAGACGATCAGATACCGTCCTAGTTCCAACCATAAACGATGCCGTCTGCGGATCGGCGGATGTTCACTTAAAGCACCGTCGGCACGCTATGAGAAATCAAAGACTTCGGGTTCCGGGGGGAGTATGTTCGCAAGAATGAAACTTAAAGGAATTGACGGAAGGGCACCACCAGGAGTGGAACCTGCGGTTTAATTTGACTCAACACGGGAAAACTCACCAGGTCCAGACATAGGAAGGATTGACAGATTGATAGCTCTTTCTTGATTCTATGGGTGGTGGTGCATGGCCGTTCTTAGTTGGTGGAGTGATTTGTCTGGTTAATTCCGTTAACGAACGAGACCTTAACCTGCTAAATAGTGACACAGTTCTTATGAAATGTGGTTCACTTCTTAGAGGGACTATCGGATTGAAGCCGATGGAAGTTTGAGGCAATAACAGGTCTGTGATGCCCTTAGATGTTCTGGGCCACACGCGCGTTACACTGATGAAGCCAGCGAGTATATCGCCTACACCGGAAGGTGCGGGTAATCTTGTGAAACTTCATCGTGCTGGGGATAGACCATTGCAATTATTGGTCTTGAACGAGGAATTCCTAGTAAGCACGAGTCATCAACTCGTGCTGATTACGTCCCTGCCCTTTGTACACACCGCCCGTCGCTACTACCGATTGAATGGTTTAGTGAGATCTCGGGATTGGCGACGCCATGCCGCAAGGCGCGGCGCCGCCGAGAACTTGCTCAAACTTGATCATTTAGAGGAAGTAAAAGTCGTAACA

AF358112_Coeloplana_agniae GCTTGTCTCAAAGATTAAGCCATGCATGTCTAAGTATAAACTTTTATACTGTGAAACTGCGAATGGCTCATTAAATCAGTTATCGTCTATTTGATTGTGCCCCTTACTACATGGATAACCGTAGTAATTCTAGAGCTAATACATGCGAAAAGTCCCGACTTACGGAAGGGATGTATTTATTAGATTAAAAACCAACGCGTTTCTCCAGAGACGCTCCAAGGTGATTCATAATAACTGTTCGAATCGCACGGCCTCCGCGCCAGCGATGTTTCATTCGAGTTTCTGCCCTATCAACTTTCGATGGTAAGGTATTGGCTTACCATGGTTACAACGGGTAACGGAGAATTAGGGTTCGATTCCGGAGAGGGAGCCCGAGAAACGGCTACCACATCCAAGGAAGGCAGCAGGCGCGCAAATTACCCAATCCCGACTCGGGGAGGTAGTGACAATAAATAACGTTGCAGGCGCCAACGGCTTCTGCAGTCGGAATGAGTACAATATAACACCCTTAACGAGGAACAATTGGAGGGCAAGTCTGGTGCCAGCAGCCGCGGTAATTCCAGCTCCAATAGCGTATATTAAAGTTGTTGCAGTTAAAAAGCTCGTAGTTGGACTTCGGAACTGGCCGATTGGTCCGCCCTC--CGGGTTGAGTACTGATCGGTCTGTTCTTCTTCGCGAAGACTGCGTGTGCCCTTAACTGGGTGTGCGTGGGATTCACGACGTTTACTTTGAAAAAATTAGAGTGTTCAAAGCAGGCAATCGCTTGAATATCTCAGCATGGAATAATAGAATAGGACTTTGGTCTTATTTTGTTGGTTTCCGAGACCGAAGTAATGATTAATAGGGACAGTTGGGGGCATTCGTATTTCATTGTCAGAGGTGAAATTCTTGGATTTATGAAAGACGAACTTCTGCGAAAGCATTTGCCAAGGATGTTTTCATTAATCAAGAACGAAAGTTGGAGGCTCGAAGACGATCAGATACCGTCCTAGTTCCAACCATAAACGATGCCGTCTGCGGATCGGCGGATGTTCACTTAAAGCACCGTCGGCACGCTATGAGAAATCAAAGACTTCGGGTTCCGGGGGGAGTATGTTCGCAAGAATGAAACTTAAAGGAATTGACGGAAGGGCACCACCAGGAGTGGAACCTGCGGTTTAATTTGACTCAACACGGGAAAACTCACCAGGTCCAGACATAGGAAGGATTGACAGATTGATAGCTCTTTCTTGATTCTATGGGTGGTGGTGCATGGCCGTTCTTAGTTGGTGGAGTGATTTGTCTGGTTAATTCCGTTAACGAACGAGACCTTAACCTGCTAAATAGTGACACAGTTCTTATGAAATGTGGTTCACTTCTTAGAGGGACTATCGGATTGAAGCCGATGGAAGTTTGAGGCAATAACAGGTCTGTGATGCCCTTAGATGTTCTGGGCCACACGCGCGTTACACTGATGAAGCCAGCGAGTATATCGCCTACACCGGAAGGTGCGGGTAATCTTGTGAAACTTCATCGTGCTGGGGATAGACCATTGCAATTATTGGTCTTGAACGAGGAATTCCTAGTAAGCACGAGTCATCAACTCGTGCTGATTACGTCCCTGCCCTTTGTACACACCGCCCGTCGCTACTACCGATTGAATGGTTTAGTGAGATCTCGGGATTGGCGACGCCATGCCGCAAGGCGCGGCGCCGCCGAGAACTTGCTCAAACTTGATCATTTAGAGGAAGTAAAAGTCGTAACA

KT885938_Coeloplana_lineolata_Red_Sea GCTTGTCTCAAAGATTAAGCCATGCATGTCTAAGTATAAACTTTTATACTGTGAAACTGCGAATGGCTCATTAAATCAGTTATCGTCTATTTGATTGTGCCCCTTACTACATGGATAACCGTAGTAATTCTAGAGCTAATACATGCGAAAAGTCCCGACTTACGGAAGGGATGTATTTATTAGATTAAAAACCAACGCGTTTCTCCAGAGACGCTCCAAGGTGATTCATAATAACTGTTCGAATCGCACGGCCTCCGCGCCAGCGATGTTTCATTCGAGTTTCTGCCCTATCAACTTTCGATGGTAAGGTATTGGCTTACCATGGTTACAACGGGTAACGGAGAATTAGGGTTCGATTCCGGAGAGGGAGCCCGAGAAACGGCTACCACATCCAAGGAAGGCAGCAGGCGCGCAAATTACCCAATCCCGACTCGGGGAGGTAGTGACAATAAATAACGTTGCAGGCGCCAACGGCTTCTGCAGTCGGAATGAGTACAATATAACACCCTTAACGAGGAACAATTGGAGGGCAAGTCTGGTGCCAGCAGCCGCGGTAATTCCAGCTCCAATAGCGTATATTAAAGTTGTTGCAGTTAAAAAGCTCGTAGTTGGACTTCGGAACTGGCCGATTGGTCCGCCCTC--CGGGTTGAGTACTGATCGGTCTGTTCTTCTTCGCGAAGACTGCGTGTGCCCTTAACTGGGTGTGCGTGGGATTCACGACGTTTACTTTGAAAAAATTAGAGTGTTCAAAGCAGGCAATCGCTTGAATATCTCAGCATGGAATAATAGAATAGGACTTTGGTCTTATTTTGTTGGTTTCCGAGACCGAAGTAATGATTAATAGGGACAGTTGGGGGCATTCGTATTTCATTGTCAGAGGTGAAATTCTTGGATTTATGAAAGACGAACTTCTGCGAAAGCATTTGCCAAGGATGTTTTCATTAATCAAGAACGAAAGTTGGAGGCTCGAAGACGATCAGATACCGTCCTAGTTCCAACCATAAACGATGCCGTCTGCGGATCGGCGGATGTTCACTTAAAGCACCGTCGGCACGCTATGAGAAATCAAAGACTTCGGGTTCCGGGGGGAGTATGTTCGCAAGAATGAAACTTAAAGGAATTGACGGAAGGGCACCACCAGGAGTGGAACCTGCGGTTTAATTTGACTCAACACGGGAAAACTCACCAGGTCCAGACATAGGAAGGATTGACAGATTGATAGCTCTTTCTTGATTCTATGGGTGGTGGTGCATGGCCGTTCTTAGTTGGTGGAGTGATTTGTCTGGTTAATTCCGTTAACGAACGAGACCTTAACCTGCTAAATAGTGACACAGTTCTTATGAAATGTGGTTCACTTCTTAGAGGGACTATCGGATTGAAGCCGATGGAAGTTTGAGGCAATAACAGGTCTGTGATGCCCTTAGATGTTCTGGGCCACACGCGCGTTACACTGATGAAGCCAGCGAGTATATCGCCTACACCGGAAGGTGCGGGTAATCTTGTGAAACTTCATCGTGCTGGGGATAGACCATTGCAATTATTGGTCTTGAACGAGGAATTCCTAGTAAGCACGAGTCATCAACTCGTGCTGATTACGTCCCTGCCCTTTGTACACACCGCCCGTCGCTACTACCGATTGAATGGTTTAGTGAGATCTCGGGATTGGCGACGCCATGCCGCAAGGCGCGGCGCCGCCGAGAACTTGCTCAAACTTGATCATTTAGAGGAAGTAAAAGTCGTAACA

KT885939_Coeloplana_yulianicorum_Red_Sea GCTTGTCTCAAAGATTAAGCCATGCATGTCTAAGTATAAACTTTTATACTGTGAAACTGCGAATGGCTCATTAAATCAGTTATCGTCTATTTGATTGTGCCCCTTACTACATGGATAACCGTAGTAATTCTAGAGCTAATACATGCGAAAAGTCCCGACTTACGGAAGGGATGTATTTATTAGATTAAAAACCAACGCGTTTCTCCAGAGACGCTCCAAGGTGATTCATAATAACTGTTCGAATCGCACGGCCTCCGCGCCAGCGATGTTTCATTCGAGTTTCTGCCCTATCAACTTTCGATGGTAAGGTATTGGCTTACCATGGTTACAACGGGTAACGGAGAATTAGGGTTCGATTCCGGAGAGGGAGCCCGAGAAACGGCTACCACATCCAAGGAAGGCAGCAGGCGCGCAAATTACCCAATCCCGACTCGGGGAGGTAGTGACAATAAATAACGTTGCAGGCGCCAACGGCTTCTGCAGTCGGAATGAGTACAATATAACACCCTTAACGAGGAACAATTGGAGGGCAAGTCTGGTGCCAGCAGCCGCGGTAATTCCAGCTCCAATAGCGTATATTAAAGTTGTTGCAGTTAAAAAGCTCGTAGTTGGACTTCGGAACTGGCCGATTGGTCCGCCCTC--CGGGTTGAGTACTGATCGGTCTGTTCTTCTTCGCGAAGACTGCGTGTGCCCTTAACTGGGTGTGCGTGGGATTCACGACGTTTACTTTGAAAAAATTAGAGTGTTCAAAGCAGGCAATCGCTTGAATATCTCAGCATGGAATAATAGAATAGGACTTTGGTCTTATTTTGTTGGTTTCCGAGACCGAAGTAATGATTAATAGGGACAGTTGGGGGCATTCGTATTTCATTGTCAGAGGTGAAATTCTTGGATTTATGAAAGACGAACTTCTGCGAAAGCATTTGCCAAGGATGTTTTCATTAATCAAGAACGAAAGTTGGAGGCTCGAAGACGATCAGATACCGTCCTAGTTCCAACCATAAACGATGCCGTCTGCGGATCGGCGGATGTTCACTTAAAGCACCGTCGGCACGCTATGAGAAATCAAAGACTTCGGGTTCCGGGGGGAGTATGTTCGCAAGAATGAAACTTAAAGGAATTGACGGAAGGGCACCACCAGGAGTGGAACCTGCGGTTTAATTTGACTCAACACGGGAAAACTCACCAGGTCCAGACATAGGAAGGATTGACAGATTGATAGCTCTTTCTTGATTCTATGGGTGGTGGTGCATGGCCGTTCTTAGTTGGTGGAGTGATTTGTCTGGTTAATTCCGTTAACGAACGAGACCTTAACCTGCTAAATAGTGACACAGTTCTTATGAAATGTGGTTCACTTCTTAGAGGGACTATCGGATTGAAGCCGATGGAAGTTTGAGGCAATAACAGGTCTGTGATGCCCTTAGATGTTCTGGGCCACACGCGCGTTACACTGATGAAGCCAGCGAGTATATCGCCTACACCGGAAGGTGCGGGTAATCTTGTGAAACTTCATCGTGCTGGGGATAGACCATTGCAATTATTGGTCTTGAACGAGGAATTCCTAGTAAGCACGAGTCATCAACTCGTGCTGATTACGTCCCTGCCCTTTGTACACACCGCCCGTCGCTACTACCGATTGAATGGTTTAGTGAGATCTCGGGATTGGCGACGCCATGCCGCAAGGCGCGGCGCCGCCGAGAACTTGCTCAAACTTGATCATTTAGAGGAAGTAAAAGTCGTAACA

KT885940_Coeloplana_fishelsoni_Red_Sea GCTTGTCTCAAAGATTAAGCCATGCATGTCTAAGTATAAACTTTTATACTGTGAAACTGCGAATGGCTCATTAAATCAGTTATCGTCTATTTGATTGTGCCCCTTACTACATGGATAACCGTAGTAATTCTAGAGCTAATACATGCGAAAAGTCCCGACTTACGGAAGGGATGTATTTATTAGATTAAAAACCAACGCGTTTCTCCAGAGACGCTCCAAGGTGATTCATAATAACTGTTCGAATCGCACGGCCTCCGCGCCAGCGATGTTTCATTCGAGTTTCTGCCCTATCAACTTTCGATGGTAAGGTATTGGCTTACCATGGTTACAACGGGTAACGGAGAATTAGGGTTCGATTCCGGAGAGGGAGCCCGAGAAACGGCTACCACATCCAAGGAAGGCAGCAGGCGCGCAAATTACCCAATCCCGACTCGGGGAGGTAGTGACAATAAATAACGTTGCAGGCGCCAACGGCTTCTGCAGTCGGAATGAGTACAATATAACACCCTTAACGAGGAACAATTGGAGGGCAAGTCTGGTGCCAGCAGCCGCGGTAATTCCAGCTCCAATAGCGTATATTAAAGTTGTTGCAGTTAAAAAGCTCGTAGTTGGACTTCGGAACTGGCCGATTGGTCCGCCCTC--CGGGTTGAGTACTGATCGGTCTGTTCTTCTTCGCGAAGACTGCGTGTGCCCTTAACTGGGTGTGCGTGGGATTCACGACGTTTACTTTGAAAAAATTAGAGTGTTCAAAGCAGGCAATCGCTTGAATATCTCAGCATGGAATAATAGAATAGGACTTTGGTCTTATTTTGTTGGTTTCCGAGACCGAAGTAATGATTAATAGGGACAGTTGGGGGCATTCGTATTTCATTGTCAGAGGTGAAATTCTTGGATTTATGAAAGACGAACTTCTGCGAAAGCATTTGCCAAGGATGTTTTCATTAATCAAGAACGAAAGTTGGAGGCTCGAAGACGATCAGATACCGTCCTAGTTCCAACCATAAACGATGCCGTCTGCGGATCGGCGGATGTTCACTTAAAGCACCGTCGGCACGCTATGAGAAATCAAAGACTTCGGGTTCCGGGGGGAGTATGTTCGCAAGAATGAAACTTAAAGGAATTGACGGAAGGGCACCACCAGGAGTGGAACCTGCGGTTTAATTTGACTCAACACGGGAAAACTCACCAGGTCCAGACATAGGAAGGATTGACAGATTGATAGCTCTTTCTTGATTCTATGGGTGGTGGTGCATGGCCGTTCTTAGTTGGTGGAGTGATTTGTCTGGTTAATTCCGTTAACGAACGAGACCTTAACCTGCTAAATAGTGACACAGTTCTTATGAAATGTGGTTCACTTCTTAGAGGGACTATCGGATTGAAGCCGATGGAAGTTTGAGGCAATAACAGGTCTGTGATGCCCTTAGATGTTCTGGGCCACACGCGCGTTACACTGATGAAGCCAGCGAGTATATCGCCTACACCGGAAGGTGCGGGTAATCTTGTGAAACTTCATCGTGCTGGGGATAGACCATTGCAATTATTGGTCTTGAACGAGGAATTCCTAGTAAGCACGAGTCATCAACTCGTGCTGATTACGTCCCTGCCCTTTGTACACACCGCCCGTCGCTACTACCGATTGAATGGTTTAGTGAGATCTCGGGATTGGCGACGCCATGCCGCAAGGCGCGGCGCCGCCGAGAACTTGCTCAAACTTGATCATTTAGAGGAAGTAAAAGTCGTAACA

KT885941_Coeloplana_fishelsoni_Red_Sea GCTTGTCTCAAAGATTAAGCCATGCATGTCTAAGTATAAACTTTTATACTGTGAAACTGCGAATGGCTCATTAAATCAGTTATCGTCTATTTGATTGTGCCCCTTACTACATGGATAACCGTAGTAATTCTAGAGCTAATACATGCGAAAAGTCCCGACTTACGGAAGGGATGTATTTATTAGATTAAAAACCAACGCGTTTCTCCAGAGACGCTCCAAGGTGATTCATAATAACTGTTCGAATCGCACGGCCTCCGCGCCAGCGATGTTTCATTCGAGTTTCTGCCCTATCAACTTTCGATGGTAAGGTATTGGCTTACCATGGTTACAACGGGTAACGGAGAATTAGGGTTCGATTCCGGAGAGGGAGCCCGAGAAACGGCTACCACATCCAAGGAAGGCAGCAGGCGCGCAAATTACCCAATCCCGACTCGGGGAGGTAGTGACAATAAATAACGTTGCAGGCGCCAACGGCTTCTGCAGTCGGAATGAGTACAATATAACACCCTTAACGAGGAACAATTGGAGGGCAAGTCTGGTGCCAGCAGCCGCGGTAATTCCAGCTCCAATAGCGTATATTAAAGTTGTTGCAGTTAAAAAGCTCGTAGTTGGACTTCGGAACTGGCCGATTGGTCCGCCCTC--CGGGTTGAGTACTGATCGGTCTGTTCTTCTTCGCGAAGACTGCGTGTGCCCTTAACTGGGTGTGCGTGGGATTCACGACGTTTACTTTGAAAAAATTAGAGTGTTCAAAGCAGGCAATCGCTTGAATATCTCAGCATGGAATAATAGAATAGGACTTTGGTCTTATTTTGTTGGTTTCCGAGACCGAAGTAATGATTAATAGGGACAGTTGGGGGCATTCGTATTTCATTGTCAGAGGTGAAATTCTTGGATTTATGAAAGACGAACTTCTGCGAAAGCATTTGCCAAGGATGTTTTCATTAATCAAGAACGAAAGTTGGAGGCTCGAAGACGATCAGATACCGTCCTAGTTCCAACCATAAACGATGCCGTCTGCGGATCGGCGGATGTTCACTTAAAGCACCGTCGGCACGCTATGAGAAATCAAAGACTTCGGGTTCCGGGGGGAGTATGTTCGCAAGAATGAAACTTAAAGGAATTGACGGAAGGGCACCACCAGGAGTGGAACCTGCGGTTTAATTTGACTCAACACGGGAAAACTCACCAGGTCCAGACATAGGAAGGATTGACAGATTGATAGCTCTTTCTTGATTCTATGGGTGGTGGTGCATGGCCGTTCTTAGTTGGTGGAGTGATTTGTCTGGTTAATTCCGTTAACGAACGAGACCTTAACCTGCTAAATAGTGACACAGTTCTTATGAAATGTGGTTCACTTCTTAGAGGGACTATCGGATTGAAGCCGATGGAAGTTTGAGGCAATAACAGGTCTGTGATGCCCTTAGATGTTCTGGGCCACACGCGCGTTACACTGATGAAGCCAGCGAGTATATCGCCTACACCGGAAGGTGCGGGTAATCTTGTGAAACTTCATCGTGCTGGGGATAGACCATTGCAATTATTGGTCTTGAACGAGGAATTCCTAGTAAGCACGAGTCATCAACTCGTGCTGATTACGTCCCTGCCCTTTGTACACACCGCCCGTCGCTACTACCGATTGAATGGTTTAGTGAGATCTCGGGATTGGCGACGCCATGCCGCAAGGCGCGGCGCCGCCGAGAACTTGCTCAAACTTGATCATTTAGAGGAAGTAAAAGTCGTAACA

KT885942_Coeloplana_bannwarthi_Red_Sea GCTTGTCTCAAAGATTAAGCCATGCATGTCTAAGTATAAACTTTTATACTGTGAAACTGCGAATGGCTCATTAAATCAGTTATCGTCTATTTGATTGTGCCCCTTACTACATGGATAACCGTAGTAATTCTAGAGCTAATACATGCGAAAAGTCCCGACTTACGGAAGGGATGTATTTATTAGATTAAAAACCAACGCGTTTCTCCAGAGACGCTCCAAGGTGATTCATAATAACTGTTCGAATCGCACGGCCTCCGCGCCAGCGATGTTTCATTCGAGTTTCTGCCCTATCAACTTTCGATGGTAAGGTATTGGCTTACCATGGTTACAACGGGTAACGGAGAATTAGGGTTCGATTCCGGAGAGGGAGCCCGAGAAACGGCTACCACATCCAAGGAAGGCAGCAGGCGCGCAAATTACCCAATCCCGACTCGGGGAGGTAGTGACAATAAATAACGTTGCAGGCGCCAACGGCTTCTGCAGTCGGAATGAGTACAATATAACACCCTTAACGAGGAACAATTGGAGGGCAAGTCTGGTGCCAGCAGCCGCGGTAATTCCAGCTCCAATAGCGTATATTAAAGTTGTTGCAGTTAAAAAGCTCGTAGTTGGACTTCGGAACTGGCCGATTGGTCCGCCCTC--CGGGTTGAGTACTGATCGGTCTGTTCTTCTTCGCGAAGACTGCGTGTGCCCTTAACTGGGTGTGCGTGGGATTCACGACGTTTACTTTGAAAAAATTAGAGTGTTCAAAGCAGGCAATCGCTTGAATATCTCAGCATGGAATAATAGAATAGGACTTTGGTCTTATTTTGTTGGTTTCCGAGACCGAAGTAATGATTAATAGGGACAGTTGGGGGCATTCGTATTTCATTGTCAGAGGTGAAATTCTTGGATTTATGAAAGACGAACTTCTGCGAAAGCATTTGCCAAGGATGTTTTCATTAATCAAGAACGAAAGTTGGAGGCTCGAAGACGATCAGATACCGTCCTAGTTCCAACCATAAACGATGCCGTCTGCGGATCGGCGGATGTTCACTTAAAGCACCGTCGGCACGCTATGAGAAATCAAAGACTTCGGGTTCCGGGGGGAGTATGTTCGCAAGAATGAAACTTAAAGGAATTGACGGAAGGGCACCACCAGGAGTGGAACCTGCGGTTTAATTTGACTCAACACGGGAAAACTCACCAGGTCCAGACATAGGAAGGATTGACAGATTGATAGCTCTTTCTTGATTCTATGGGTGGTGGTGCATGGCCGTTCTTAGTTGGTGGAGTGATTTGTCTGGTTAATTCCGTTAACGAACGAGACCTTAACCTGCTAAATAGTGACACAGTTCTTATGAAATGTGGTTCACTTCTTAGAGGGACTATCGGATTGAAGCCGATGGAAGTTTGAGGCAATAACAGGTCTGTGATGCCCTTAGATGTTCTGGGCCACACGCGCGTTACACTGATGAAGCCAGCGAGTATATCGCCTACACCGGAAGGTGCGGGTAATCTTGTGAAACTTCATCGTGCTGGGGATAGACCATTGCAATTATTGGTCTTGAACGAGGAATTCCTAGTAAGCACGAGTCATCAACTCGTGCTGATTACGTCCCTGCCCTTTGTACACACCGCCCGTCGCTACTACCGATTGAATGGTTTAGTGAGATCTCGGGATTGGCGACGCCATGCCGCAAGGCGCGGCGCCGCCGAGAACTTGCTCAAACTTGATCATTTAGAGGAAGTAAAAGTCGTAACA

KT885943_Coeloplana_loyai_Red_Sea GCTTGTCTCAAAGATTAAGCCATGCATGTCTAAGTATAAACTTTTATACTGTGAAACTGCGAATGGCTCATTAAATCAGTTATCGTCTATTTGATTGTGCCCCTTACTACATGGATAACCGTAGTAATTCTAGAGCTAATACATGCGAAAAGTCCCGACTTACGGAAGGGATGTATTTATTAGATTAAAAACCAACGCGTTTCTCCAGAGACGCTCCAAGGTGATTCATAATAACTGTTCGAATCGCACGGCCTCCGCGCCAGCGATGTTTCATTCGAGTTTCTGCCCTATCAACTTTCGATGGTAAGGTATTGGCTTACCATGGTTACAACGGGTAACGGAGAATTAGGGTTCGATTCCGGAGAGGGAGCCCGAGAAACGGCTACCACATCCAAGGAAGGCAGCAGGCGCGCAAATTACCCAATCCCGACTCGGGGAGGTAGTGACAATAAATAACGTTGCAGGCGCCAACGGCTTCTGCAGTCGGAATGAGTACAATATAACACCCTTAACGAGGAACAATTGGAGGGCAAGTCTGGTGCCAGCAGCCGCGGTAATTCCAGCTCCAATAGCGTATATTAAAGTTGTTGCAGTTAAAAAGCTCGTAGTTGGACTTCGGAACTGGCCGATTGGTCCGCCCTY--CGGGTTGAGTACTGATCGGTCTGTTCTTCTTCGCGAAGACTGCGTGTGCCCTTAACTGGGTGTGCGTGGGATTCACGACGTTTACTTTGAAAAAATTAGAGTGTTCAAAGCAGGCAATCGCTTGAATATCTCAGCATGGAATAATAGAATAGGACTTTGGTCTTATTTTGTTGGTTTCCGAGACCGAAGTAATGATTAATAGGGACAGTTGGGGGCATTCGTATTTCATTGTCAGAGGTGAAATTCTTGGATTTATGAAAGACGAACTTCTGCGAAAGCATTTGCCAAGGATGTTTTCATTAATCAAGAACGAAAGTTGGAGGCTCGAAGACGATCAGATACCGTCCTAGTTCCAACCATAAACGATGCCGTCTGCGGATCGGCGGATGTTCACTTAAAGCACCGTCGGCACGCTATGAGAAATCAAAGACTTCGGGTTCCGGGGGGAGTATGTTCGCAAGAATGAAACTTAAAGGAATTGACGGAAGGGCACCACCAGGAGTGGAACCTGCGGTTTAATTTGACTCAACACGGGAAAACTCACCAGGTCCAGACATAGGAAGGATTGACAGATTGATAGCTCTTTCTTGATTCTATGGGTGGTGGTGCATGGCCGTTCTTAGTTGGTGGAGTGATTTGTCTGGTTAATTCCGTTAACGAACGAGACCTTAACCTGCTAAATAGTGACACAGTTCTTATGAAATGTGGTTCACTTCTTAGAGGGACTATCGGATTGAAGCCGATGGAAGTTTGAGGCAATAACAGGTCTGTGATGCCCTTAGATGTTCTGGGCCACACGCGCGTTACACTGATGAAGCCAGCGAGTATATCGCCTACACCGGAAGGTGCGGGTAATCTTGTGAAACTTCATCGTGCTGGGGATAGACCATTGCAATTATTGGTCTTGAACGAGGAATTCCTAGTAAGCACGAGTCATCAACTCGTGCTGATTACGTCCCTGCCCTTTGTACACACCGCCCGTCGCTACTACCGATTGAATGGTTTAGTGAGATCTCGGGATTGGCGACGCCATGCCGCAAGGCGCGGCGCCGCCGAGAACTTGCTCAAACTTGATCATTTAGAGGAAGTAAAAGTCGTAACA

HQ435813_Coeloplana_bocki GCTTGTCTCAAAGATTAAGCCATGCATGTCTAAGTATAAACTTTTATACTGTGAAACTGCGAATGGCTCATTAAATCAGTTATCGTCTATTTGATTGTGCCCCTTACTACATGGATAACCGTAGTAATTCTAGAGCTAATACATGCGAAAAGTCCCGACTTACGGAAGGGATGTATTTATTAGATTAAAAACCAACGCGTTTCTCCAGAGACGCTCCAAGGTGATTCATAATAACTGTTCGAATCGCACGGCCTCCGCGCCAGCGATGTTTCATTCGAGTTTCTGCCCTATCAACTTTCGATGGTAAGGTATTGGCTTACCATGGTTACAACGGGTAACGGAGAATTAGGGTTCGATTCCGGAGAGGGAGCCCGAGAAACGGCTACCACATCCAAGGAAGGCAGCAGGCGCGCAAATTACCCAATCCCGACTCGGGGAGGTAGTGACAATAAATAACGTTGCAGGCGCCAACGGCTTCTGCAGTCGGAATGAGTACAATATAACACCCTTAACGAGGAACAATTGGAGGGCAAGTCTGGTGCCAGCAGCCGCGGTAATTCCAGCTCCAATAGCGTATATTAAAGTTGTTGCAGTTAAAAAGCTCGTAGTTGGACTTCGGAACCGGCCGATTGGTCCGCCCTT--CGGGTTGAGTACTGATCGGTCTGTTCTTCTTCGCGAAGACTGCGTGTGCCCTTAACTGGGTGTGCGTGGGATTCACGACGTTTACTTTGAAAAAATTAGAGTGTTCAAAGCAGGCAATCGCTTGAATATCTCAGCATGGAATAATAGAATAGGACTTTGGTCTTATTTTGTTGGTTTCCGAGACCGAAGTAATGATTAATAGGGACAGTTGGGGGCATTCGTATTTCATTGTCAGAGGTGAAATTCTTGGATTTATGAAAGACGAACTTCTGCGAAAGCATTTGCCAAGGATGTTTTCATTAATCAAGAACGAAAGTTGGAGGCTCGAAGACGATCAGATACCGTCCTAGTTCCAACCATAAACGATGCCGTCTGCGGATCGGCGGATGTTCACTTAAAGCACCGTCGGCACGCTATGAGAAATCAAAGACTTCGGGTTCCGGGGGGAGTATGTTCGCAAGAATGAAACTTAAAGGAATTGACGGAAGGGCACCACCAGGAGTGGAACCTGCGGTTTAATTTGACTCAACACGGGAAAACTCACCAGGTCCAGACATAGGAAGGATTGACAGATTGATAGCTCTTTCTTGATTCTATGGGTGGTGGTGCATGGCCGTTCTTAGTTGGTGGAGTGATTTGTCTGGTTAATTCCGTTAACGAACGAGACCTTAACCTGCTAAATAGTGACACAGTTCTTATGAAATGTGGTTCACTTCTTAGAGGGACTATCGGATTGAAGCCGATGGAAGTTTGAGGCAATAACAGGTCTGTGATGCCCTTAGATGTTCTGGGCCACACGCGCGTTACACTGATGAAGCCAGCGAGTATATCGCCTACACCGGAAGGTGCGGGTAATCTTGTGAAACTTCATCGTGCTGGGGATAGACCCTTGCAATTATGGGTCTTGAACGAGGAATTCCTAGTAAGCACGAGTCATCAACTCGTGCTGATTACGTCCCTGCCCTTTGTACACACCGCCCGTCGCTACTACCGATTGAATGGTTTAGTGAGATCTCGGGATTGGCGACGCCATGCCGCAAGGCGCGGCGCCGCCGAGAACTTGCTCAAACTTGATCATTTAGAGGAAGTAAAAGTCGTAACA

AF293683_Coeloplana_bannwarthii GCTTGTCTCAAAGATTAAGCCATGCATGTCTAAGTATAAACGTTTATACTGTGAAACTGCAAATGGCTCATTAAATCAGTTATCGTCTATTTGATTGTGCCCC-TACTACATGGATAACCGTAGTAATTCTAGAGCTAATACATGCGAAAAGTCCCGACTTACGGAAGGGATGTATTTATTAGATTAAAAACCAACGCGTTTCTCCAGAGACGCTCCAAGGTGATTCATAATAACTGTTCGAATCGCACGGCCTCCGCGCCAGCGATGTTTCATTCGAGTTTCTGCCCTATCAACTTTCGATGGTAAGGTATTGGCTTACCATGGTTACAACGGGTAACGGAGAATTAGGGTTCGATTCCGGAGAGGGAGCCCGAGAAACGGCTACCACATCCAAGGAAGGCAGCAGGCGCGCAAATTACCCAATCCCGACTCGGGGAGGTAGTGACAATAAATAACGTTGCAGGCGCCAACGGCTTCTGCAGTCGGAATGAGTACAATATAACACCCTTAACGAGGAACAATTGGAGGGCAAGTCTGGTGCCAGCAGCCGCGGTAATTCCAGCTCCAATAGCGTATATTAAAGTTGTTGCAGTTAAAAAGCTCGTAGTTGGACTTCGGAACTGGCCGATTGGTCCGCCCTCCGCGGGTTGAGTACTGATCGGTCTGTTCTTCTTCGCGAAGACTGCGTGTGCCCTTAACTGGGTGTGCGTGGGATTCACGACGTTTACTTTGAAAAAATTAGAGTGTTCAAAGCAGGCAATCGCTTGAATATCTCAGCATGGAATAATAGAATAGGACTTTGGTCTTATTTTGTTGGTTTCCGAGACCGAAGTAATGATTAATAGGGACAGTTGGGGGCATTCGTATTTCATTGTCAGAGGTGAAATTCTTGGATTTATGAAAGACGAACTTCTGCGAAAGCATTTGCCAAGGATGTTTTCATTAATCAAGAACGAAAGTTGGAGGCTCGAAGACGATCAGATACCGTCCTAGTTCCAACCATAAACGATGCCGTCTGCGGATCGGCGGATGTTCACTTAAAGCACCGTCGGCACGCTATGAGAAATCAAAGACTTCGGGTTCCGGGGGGAGTATGTTCGCAAGAATGAAACTTAAAGGAATTGACGGAAGGGCACCACCAGGAGTGGAA-CTGCGGTTTAATTTGACTCAACACGGGAAAACTCACCAGGTCCAGACATAGGAAGGATTGACAGATTGATAGCTCTTTCTTGATTCTATGGGTGGTGGTGCATGGCCGTTCTTAGTTGGTGGAGTGATTTGTCTGGTTAATTCCGTTAACGAACGAGACCTTAACCTGCTAAATAGTGACACAGTTCTTATGAAATGTGGTTCACTTCTTAGAGGGACTATCGGATTGAAGCCGATGGAAGTTTGAGGCAATAACAGGTCTGTGATGCCCTTAGATGTTCTGGGCCACACGCGCGTTACACTGATGAAGCCAGCGAGTATATCGCCTACACCGGAAGGTGCGGGTAATCTTGTGAAACTTCATCGTGCTGGGGATAGACCATTGCAATTATTGGTCTTGAACGAGGAATTCCTAGTAAGCACGAGTCATCAACTCGTGCTGATTACGTCCCTGCCCTTTGTACACACCGCCCGTCGCTACTACCGATTGAATGGTTTAGTGAGATCTCGGGATTGGCGACGCCATGCCGCAAGGCGCGGCGCCGCCGAGAACTTGCTCAAACTTGATCATTTAGAGGAAGTAAAAGTCGTAACA

AF293684_Vallicula_multiformis GCTTGTCTCAAAGATTAAGCCATGCATGTCTAAGTATAAGCTTTTATACTGTGAAACTGCGAATGGCTCATTAAATCAGTTATCGTCTATTTGATTGTGCCCC-TACTACATGGATAACCGTAGTAATTCTAGAGCTAATACATGCGAAAAGTCCCGACTCCTGGAAGGGATGTATTTATTAGATTAAAAACCAATGCGTTTCTTCAGAGACGCTCCAAGGTGATTCATAATAACTGTTCGAATCGCACGGCCTCCGCGCCAGCGATGTTTCATTCGAGTTTCTGCCCTATCAACTTTCGATGGTAAGGTATTGGCTTACCATGGTTACAACGGGTAACGGAGAATTAGGGTTCGATTCCGGAGAGGGAGCCCGAGAAACGGCTACCACATCCAAGGAAGGCAGCAGGCGCGCAAATTACCCAATCCCGACTCGGGGAGGTAGTGACAATAAATAACGTTGCAGGCGCCAACGGCTTCTGCAGTCGGAATGAGTACAATATAACACCCTTAACGAGGAACAATTGGAGGGCAAGTCTGGTGCCAGCAGCCGCGGTAATTCCAGCTCCAATAGCGTATATTAAAGTTGTTGCAGTTAAAAAGCTCGTAGTTGGACTTCGGAACTGGCCGATTGGTCCGCCCTT--CGGGTTGTGTACTGATCGGTTTGTTCTTCTTCGCGAAGACCGCGTGTGCCCTTAACTGGGTGGGCGTGGGATTCGCGACGTTTACTTTGAAAAAATTAGAGTGTTCAAAGCAGGCCTTCGCTTGAATATCTCAGCATGGAATAATAGAATAGGACTTTGGTCTTATTTTGTTGGTTTCCGAGACCGAAGTAATGATTAATAGGGACAGTTGGGGGCATTCGTATTTCATTGTCAGAGGTGAAATTCTTGGATTTATGAAAGACGAACTTCTGCGAAAGCATTTGCCAAGGATGTTTTCATTAATCAAGAACGAAAGTTGGAGGCTCGAAGACGATCAGATACCGTCCTAGTTCCAACCATAAACGATGCCGTCTGCGGATCGGCGGATGCTCATTTAAGGCACCGTCGGCACGCTATGAGAAATCAAAGACTTCGGGTTCCGGGGGGAGTATGTTCGCAAGAATGAAACTTAAAGGAATTGACGGAAGGGCACCACCAGGAGTGGAA-CTGCGGTTTAATTTGACTCAACACGGGAAAACTCACCAGGTCCAGACATAGGAAGGATTGACAGATTGATAGCTCTTTCTTGATTCTATGGGTGGTGGTGCATGGCCGTTCTTAGTTGGTGGAGTGATTTGTCTGGTTAATTCCGTTAACGAACGAGACCTTAACCTGCTAAATAGTGACACCGTTCTT-TGAACTGTGGTTCACTTCTTAGAGGGACTATCGGATTGAAGCCGATGGAAGTTTGAGGCAATAACAGGTCTGTGATGCCCTTAGATGTTCTGGGCCACACGCGCGTTACACTGATGAAGCCAGCGAGTATTTCGCCTTCACCGGAAGGTGCGGGTAATCTTGTGAAACTTTATCGTGCTGGGGATAGACCATTGCAATTATTGGTCTTGAACGAGGAATTCCTAGTAAGCACGAGTCATCAACTCGTGCTGATTACGTCCCTGCCCTTTGTACACACCGCCCGTCGCTACTACCGATTGAATGGTTTAGTGAGATCTCGGGATTGGCGACGCCATGTCTCACGACGCGGCGCCGCCGAAAACTTGCTCAAACTTGATCATTTAGAGGAAGTAAAAGTCGTAACA

KT885944_Vallicula_multiformis_Red_Sea GCTTGTCTCAAAGATTAAGCCATGCATGTCTAAGTATAAGCTTTTATACTGTGAAACTGCGAATGGCTCATTAAATCAGTTATCGTCTATTTGATTGTGCCCC-TACTACATGGATAACCGTAGTAATTCTAGAGCTAATACATGCGAAAAGTCCCGACTCCTGGAAGGGATGTATTTATTAGATTAAAAACCAATGCGTTTCTTCAGAGACGCTCCAAGGTGATTCATAATAACTGTTCGAATCGCACGGCCTCCGCGCCAGCGATGTTTCATTCGAGTTTCTGCCCTATCAACTTTCGATGGTAAGGTATTGGCTTACCATGGTTACAACGGGTAACGGAGAATTAGGGTTCGATTCCGGAGAGGGAGCCCGAGAAACGGCTACCACATCCAAGGAAGGCAGCAGGCGCGCAAATTACCCAATCCCGACTCGGGGAGGTAGTGACAATAAATAACGTTGCAGGCGCCAACGGCTTCTGCAGTCGGAATGAGTACAATATAACACCCTTAACGAGGAACAATTGGAGGGCAAGTCTGGTGCCAGCAGCCGCGGTAATTCCAGCTCCAATAGCGTATATTAAAGTTGTTGCAGTTAAAAAGCTCGTAGTTGGACTTCGGAACTGGCCGATTGGTCCGCCCTT--CGGGTTGTGTACTGATCGGTTTGTTCTTCTTCGCGAAGACCGCGTGTGCCCTTAACTGGGTGGGCGTGGGATTCGCGACGTTTACTTTGAAAAAATTAGAGTGTTCAAAGCAGGCCTTCGCTTGAATATCTCAGCATGGAATAATAGAATAGGACTTTGGTCTTATTTTGTTGGTTTCCGAGACCGAAGTAATGATTAATAGGGACAGTTGGGGGCATTCGTATTTCATTGTCAGAGGTGAAATTCTTGGATTTATGAAAGACGAACTTCTGCGAAAGCATTTGCCAAGGATGTTTTCATTAATCAAGAACGAAAGTTGGAGGCTCGAAGACGATCAGATACCGTCCTAGTTCCAACCATAAACGATGCCGTCTGCGGATCGGCGGATGCTCATTTAAGGCACCGTCGGCACGCTATGAGAAATCAAAGACTTCGGGTTCCGGGGGGAGTATGTTCGCAAGAATGAAACTTAAAGGAATTGACGGAAGGGCACCACCAGGAGTGGAACCTGCGGTTTAATTTGACTCAACACGGGAAAACTCACCAGGTCCAGACATAGGAAGGATTGACAGATTGATAGCTCTTTCTTGATTCTATGGGTGGTGGTGCATGGCCGTTCTTAGTTGGTGGAGTGATTTGTCTGGTTAATTCCGTTAACGAACGAGACCTTAACCTGCTAAATAGTGACACCGTTCTT-TGAACTGTGGTTCACTTCTTAGAGGGACTATCGGATTGAAGCCGATGGAAGTTTGAGGCAATAACAGGTCTGTGATGCCCTTAGATGTTCTGGGCCACACGCGCGTTACACTGATGAAGCCAGCGAGTATTTCGCCTTCACCGGAAGGTGCGGGTAATCTTGTGAAACTTTATCGTGCTGGGGATAGACCATTGCAATTATTGGTCTTGAACGAGGAATTCCTAGTAAGCACGAGTCATCAACTCGTGCTGATTACGTCCCTGCCCTTTGTACACACCGCCCGTCGCTACTACCGATTGAATGGTTTAGTGAGATCTCGGGATTGGCGACGCCATGTCTCACGACGCGGCGCCGCCGAAAACTTGCTCAAACTTGATCATTTAGAGGAAGTAAAAGTCGTAACA

;

end;

**28S nucleotide alignment. DNA sequence alignment, in Nexus format, used to reconstruct the phylogenetic tree presented in Figure 2**

#NEXUS

Begin data;

Dimensions ntax=14 nchar=721;

Format datatype=DNA gap=- missing=? matchchar=.;

Matrix

KT885945_Coeloplana_bannwarthi_Red_Sea AACTAACCAGGATTCCCCTAGTAATGGCGAACGAACAGGGAACAGCTCAAATTTTAAATCTCGGGCGCCTGCGTCCGCGAATTGTAGTTTCCAGAAACGTTATCCCCAGGAGGACCACCGGTCTAAGTTGCTTGGAACAGCATATCGGAGAGGGTGAGAATCCCGTTTTCGGCCGGAGGTTCCCTGGCACGAGGCGTTTTCCAAGAGTCGGGTTGTTTGGGATTGCAGCCCAAAAGGCGTGGTATACCCCACGTAAAGCTAAATATTGGCACGAGACCGATAGCGAACAAGTACCATGAGGGAAAGATGAAAAGCACTTTGAAAAGAGAGTTAAACAGCACGTGAAACCGTTAAAAGGGAAACGAATGGAGCCAGCAAGGCACCCAGTTTCATTCAGCCGGTGGCCTACCA-CCAAGGCATGGGCCGACCTT--CGGGCGGTGCTTGTTTTGCGATGATAGGTTGCCGTGTGCACTTATGCTGGGTGCGTGCCAACTTGGGCTGGTGACGGTCGACACGGTCGGAAGGGAAGGTAGCTTTCTCCG-GGGAGTGTTATAGCCCTCCGTCCCGGGCCGTCGCTGGCTGAGGGGTCGCGGTACGTGCTCTCTTGGGCTTGGGCCCTGTAGCAGGTCGACTGCGCCTTCCGTTGACTGCACGCAGTGAGCGGAGGGACGACGGTAGGCTAGCCGTGGGTGTCCCGTAAACGTACCTAGGAAGTTG

KT885946_Coeloplana_brown_dots_Red_Sea AACTAACCAGGATTCCCCTAGTAATGGCGAACGAACAGGGAACAGCTCAAATTTTAAATCTCGGGCGCCTGCGTCCGCGAATTGTAGTTTCCAGAAACGTTATCCCCAGGAGGACCACCGGTCTAAGTTGCTTGGAACAGCATATCGGAGAGGGTGAGAATCCCGTTTTCGGCCGGAGGTTCCCTGGCACGAGGCGTTTTCCAAGAGTCGGGTTGTTTGGGATTGCAGCCCAAAAGGCGTGGTATACCCCACGTAAAGCTAAATATTGGCACGAGACCGATAGCGAACAAGTACCATGAGGGAAAGATGAAAAGCACTTTGAAAAGAGAGTTAAACAGCACGTGAAACCGTTAAAAGGGAAACGAATGGAGCCAGCAAGGCACCCAGTTTCATTCAGCCGGTGGCCTACCA-CCAAGGCATGGGCCGACCTT--CGGGCGGTGCTTGTTTTGCGATGATAGGTTGCCGTGTGCACTTATGCTGGGTGCGTGCCAACTTGGGCTGGTGACGGTCGACACGGTCGGAAGGGAAGGTAGCTTTCTCCG-GGGAGTGTTATAGCCCTCCGTCCCGGGCCGTCGCTGGCTGAGGGGTCGCGGTACGTGCTCTCTTGGGCTTGGGCCCTGTAGCAGGTCGACTGCGCCTTCCGTTGACTGCACGCAGTGAGCGGAGGGACGACGGTAGGCTAGCCGTGGGTGTCCCGTAAACGTACCTAGGAAGTTG

KT885947_Coeloplana_huchonae_Red_Sea AACTAACCAGGATTCCCCTAGTAATGGCGAACGAACAGGGAACAGCTCAAATTTTAAATCTCGGGCGCCTGCGTCCGCGAATTGTAGTTTCCAGAAACGTTATCCCCAGGAGGACCACCGGTCTAAGTTGCTTGGAACAGCATATCGGAGAGGGTGAGAATCCCGTTTTCGGCCGGAGGTTCCCTGGCACGAGGCGTTTTCCAAGAGTCGGGTTGTTTGGGATTGCAGCCCAAAAGGCGTGGTATACCCCACGTAAAGCTAAATATTGGCACGAGACCGATAGCGAACAAGTACCATGAGGGAAAGATGAAAAGCACTTTGAAAAGAGAGTTAAACAGCACGTGAAACCGTTAAAAGGGAAACGAATGGAGCCAGCAAGGCACCCAGTTTCATTCAGCCGGTGGCCTACCA-CCAAGGCATGGGCCGACCTT--CGGGCGGTGCTTGTTTTGCGATGATAGGTTGCCGTGTGCACTTATGCTGGGTGCGTGCCAACTTGGGCTGGTGACGGTCGACACGGTCGGAAGGGAAGGTAGCTTTCTCCG-GGGAGTGTTATAGCCCTCCGTCCCGGGCCGTCGCTGGCTGAGGGGTCGCGGTACGTGCTCTCTTGGGCTTGGGCCCTGTAGCAGGTCGACTGCGCCTTCCGTTGACTGCACGCAGTGAGCGGAGGGACGACGGTAGGCTAGCCGTGGGTGTCCCGTAAACGTACCTAGGAAGTTG

KT885948_Coeloplana_punctata_Red_Sea AACTAACCAGGATTCCCCTAGTAATGGCGAACGAACAGGGAACAGCTCAAATTTTAAATCTCGGGCGCCTGCGTCCGCGAATTGTAGTTTCCAGAAACGTTATCCCCAGGAGGACCACCGGTCTAAGTTGCTTGGAACAGCATATCGGAGAGGGTGAGAATCCCGTTTTCGGCCGGAGGTTCCCTGGCACGAGGCGTTTTCCAAGAGTCGGGTTGTTTGGGATTGCAGCCCAAAAGGCGTGGTATACCCCACGTAAAGCTAAATATTGGCACGAGACCGATAGCGAACAAGTACCATGAGGGAAAGATGAAAAGCACTTTGAAAAGAGAGTTAAACAGCACGTGAAACCGTTAAAAGGGAAACGAATGGAGCCAGCAAGGCACCCAGTTTCATTCAGCCGGTGGCCTACCA-CCAAGGCATGGGCCGACCTT--CGGGCGGTGCTTGTTTTGCGATGATAGGTTGCCGTGTGCACTTATGCTGGGTGCGTGCCAACTTGGGCTGGTGACGGTCGACACGGTCGGAAGGGAAGGTAGCTTTCTCCG-GGGAGTGTTATAGCCCTCCGTCCCGGGCCGTCGCTGGCTGAGGGGTCGCGGTACGTGCTCTCTTGGGCTTGGGCCCTGTAGCAGGTCGACTGCGCCTTCCGTTGACTGCACGCAGTGAGCGGAGGGACGACGGTAGGCTAGCCGTGGGTGTCCCGTAAACGTACCTAGGAAGTTG

KT885949_Coeloplana_lineolata_Red_Sea AACTAACCAGGATTCCCCTAGTAATGGCGAACGAACAGGGAACAGCTCAAATTTTAAATCTCGGGCGCCTGCGTCCGCGAATTGTAGTTTCCAGAAACGTTATCCCCAGGAGGACCACCGGTCTAAGTTGCTTGGAACAGCATATCGGAGAGGGTGAGAATCCCGTTTTCGGCCGGAGGTTCCCTGGCACGAGGCGTTTTCCAAGAGTCGGGTTGTTTGGGATTGCAGCCCAAAAGGCGTGGTATACCCCACGTAAAGCTAAATATTGGCACGAGACCGATAGCGAACAAGTACCATGAGGGAAAGATGAAAAGCACTTTGAAAAGAGAGTTAAACAGCACGTGAAACCGTTAAAAGGGAAACGAATGGAGCCAGCAAGGCACCCAGTTTCATTCAGCCGGTGGCCTACCA-CCAAGGCATGGGCCGACCTT--CGGGCGGTGCTTGTTTTGCGATGATAGGTTGCCGTGTGCACTTATGCTGGGTGCGTGCCAACTTGGGCTGGTGACGGTCGACACGGTCGGAAGGGAAGGTAGCTTTCTCCG-GGGAGTGTTATAGCCCTCCGTCCCGGGCCGTCGCTGGCTGAGGGGTCGCGGTACGTGCTCTCTTGGGCTTGGGCCCTGTAGCAGGTCGACTGCGCCTTCCGTTGACTGCACGCAGTGAGCGGAGGGACGACGGTAGGCTAGCCGTGGGTGTCCCGTAAACGTACCTAGGAAGTTG

KT885950_Coeloplana_fishelsoni_Red_Sea AACTAACCAGGATTCCCCTAGTAATGGCGAACGAACAGGGAACAGCTCAAATTTTAAATCTCGGGCGCCTGCGTCCGCGAATTGTAGTTTCCAGAAACGTTATCCCCAGGAGGACCACCGGTCTAAGTTGCTTGGAACAGCATATCGGAGAGGGTGAGAATCCCGTTTTCGGCCGGAGGTTCCCTGGCACGAGGCGTTTTCCAAGAGTCGGGTTGTTTGGGATTGCAGCCCAAAAGGCGTGGTATACCCCACGTAAAGCTAAATATTGGCACGAGACCGATAGCGAACAAGTACCATGAGGGAAAGATGAAAAGCACTTTGAAAAGAGAGTTAAACAGCACGTGAAACCGTTAAAAGGGAAACGAATGGAGCCAGCAAGGCACCCAGTTTCATTCAGCCGGTGGCCTACCA-CCAAGGCATGGGCCGACCTT--CGGGCGGTGCTTGTTTTGCGATGATAGGTTGCCGTGTGCACTTATGCTGGGTGCGTGCCAACTTGGGCTGGTGACGGTCGACACGGTCGGAAGGGAAGGTAGCTTTCTCCG-GGGAGTGTTATAGYCCTCCGTCCCGGGCCGTCGCTGGCTGAGGGGTCGCGGTACGTGCTCTCTTGGGCTTGGGCCCTGTAGCAGGTCGACTGCGCCTTCCGTTGACTGCACGCAGTGAGCGGAGGGACGACGGTAGGCTAGCCGTGGGTGTCCCGTAAACGTACCTAGGAAGTTG

KT885951_Coeloplana_loyai_Red_Sea AACTAACCAGGATTCCCCTAGTAATGGCGAACGAACAGGGAACAGCTCAAATTTTAAATCTCGGGCGCCTGCGTCCGCGAATTGTAGTTTCCAGAAACGTTATCCCCAGGAGGACCACCGGTCTAAGTTGCTTGGAACAGCATATCGGAGAGGGTGAGAATCCCGTTTTCGGCCGGAGGTTCCCTGGCACGAGGCGTTTTCCAAGAGTCGGGTTGTTTGGGATTGCAGCCCAAAAGGCGTGGTATACCCCACGTAAAGCTAAATATTGGCACGAGACCGATAGCGAACAAGTACCATGAGGGAAAGATGAAAAGCACTTTGAAAAGAGAGTTAAACAGCACGTGAAACCGTTAAAAGGGAAACGAATGGAGCCAGCAAGGCACCCAGTTTCATTCAGCCGGTGGCCTACCA-CCGAGGCCTGGGCCGACCTT--CGGGCGGTGCGGGTTTCGCGATGATAGGTTGCCGTGTGCACTTATGCTGGGTGCGTGCCAACTTGGGCTGGTGACGGTCGACACGGTCGGAAGGGAAGGTAGCTTTCTCCG-GGGAGTGTTATAGCCCTCCGTCCCGGGCCGTCGCTGGCTGAGGGGTCGCGGTACGTGCTCTCTTGGGCTTGGGCCCTGTAGCAGGTCGACTGCGCCTTCCGTTGACTGCACGCAGTGAGCGGAGGGACGACGGTAGGCTAGCCGTGGGTGTCCCGTAAACGTACCTAGGAAGTTG

KT885952_Coeloplana_yulianicorum_Red_Sea AACTAACCAGGATTCCCCTAGTAATGGCGAACGAACAGGGAACAGCTCAAATTTTAAATCTCGGGCGCCTGCGTCCGCGAATTGTAGTTTCCAGAAACGTTATCCCCAGGAGGACCACCGGTCTAAGTTGCTTGGAACAGCATATCGGAGAGGGTGAGAATCCCGTTTTCGGCCGGAGGTTCCCTGGCACGAGGCGTTTTCCAAGAGTCGGGTTGTTTGGGATTGCAGCCCAAAAGGCGTGGTATACCCCACGTAAAGCTAAATATTGGCACGAGACCGATAGCGAACAAGTACCATGAGGGAAAGATGAAAAGCACTTTGAAAAGAGAGTTAAACAGCACGTGAAACCGTTAAAAGGGAAACGAATGGAGCCAGCAAGGCACCCAGTTTCATTCAGCCGGTGGCCTACCA-CCGAGGCCTGGGCCGACCTT--CGGGCGGTGCGGGTTTTGCGATGATAGGTTGCCGTGTGCACTTATGCTGGGTGCGTGCCAACTTGGGCTGGTGACGGTCGACACGGTCGGAAGGGAAGGTAGCTTTCTCCG-GGGAGTGTTATAGCCCTCCGTCCCGGGCCGTCGCTGGCTGAGGGGTCGCGGTACGTGCTCTCTTGGGCTTGGGCCCTGTAGCAGGTCGACTGCGCCTTCCGTTGACTGCACGCAGTGAGCGGAGGGACGACGGTAGGCTAGCCGTGGGTGTCCCGTAAACGTACCTAGGAAGTTG

KT885953_Coeloplana_astericola_Malaysia AACTAACCAGGATTCCCCTAGTAATGGCGAACGAACAGGGAACAGCTCAAATTTTAAATCTCGGGCGCCTGCGTCCGCGAATTGTAGTTTCCAGAAACGTTATCCCCAGGAGGACCACCGGTCTAAGTTGCTTGGAACAGCATATCGGAGAGGGTGAGAATCCCGTTTTCGGCCGGAGGTTCCCTGGCACGAGGCGTTTTCCAAGAGTCGGGTTGTTTGGGATTGCAGCCCAAAAGGCGTGGTATACCCCACGTAAAGCTAAATATTGGCACGAGACCGATAGCGAACAAGTACCATGAGGGAAAGATGAAAAGCACTTTGAAAAGAGAGTTAAACAGCACGTGAAACCGTTAAAAGGGAAACGAATGGAGCCAGCAAGGCACCCAGTTTCATTCAGCCGGTGGCCTACCA-CCGAGGCCTGGGCCGACCTT--CGGGCGGTGCGGGTTTCGCGATGATAGGTTGCCGTGTGCACTTATGCTGGGTGCGTGCCAACTTGGGCTGGTGACGGTCGACACGGTCGGAAGGGAAGGTAGCTTTCTCCG-GGGAGTGTTATAGCCCTCCGTCCCGGGCCGTCGCTGGCTGAGGTGTCGCGGTACGTGCTCTCTTGGGCTTGGGCCCTGTAGCAGGTCGACTGCGCCTTCCGTTGACTGCACGCAGTGAGCGGAGGGACGACGGTAGGCTAGCCGTGGGTGTCCCGTAAACGTACCTAGGAAGTTG

KT885954_Coeloplana_sp_2_Malaysia AACTAACCAGGATTCCCCTAGTAATGGCGAACGAACAGGGAACAGCTCAAATTTTAAATCTCGGGCGCCTGCGTCCGCGAATTGTAGTTTCCAGAAACGTTATCCCCAGGAGGACCACCGGTCTAAGTTGCTTGGAACAGCATATCGGAGAGGGTGAGAATCCCGTTTTCGGCCGGAGGTTCCCTGGCACGAGGCGTTTTCCAAGAGTCGGGTTGTTTGGGATTGCAGCCCAAAAGGCGTGGTATACCCCACGTAAAGCTAAATATTGGCACGAGACCGATAGCGAACAAGTACCATGAGGGAAAGATGAAAAGCACTTTGAAAAGAGAGTTAAACAGCACGTGAAACCGTTAAAAGGGAAACGAATGGAGCCAGCAAGGCACCCAGTTTCATTCAGCCGGTGGCCTACCA-CCGAGGCCTGGGCCGACCTT--CGGGCGGTGCGGGTTTCGCGATGATAGGTTGCCGTGTGCACTTATGCTGGGTGCGTGCCAACTTGGGCTGGTGACGGTCGACACGGTCGGAAGGGAAGGTAGCTTTCTCCG-GGGAGTGTTATAGCCCTCCGTCCCGGGCCGTCGCTGGCTGAGGTGTCGCGGTACGTGCTCTCTTGGGCTTGGGCCCTGTAGCAGGTCGACTGCGCCTTCCGTTGACTGCACGCAGTGAGCGGAGGGACGACGGTAGGCTAGCCGTGGGTGTCCCGTAAACGTACCTAGGAAGTTG

KT885955_Coeloplana_sp_2_Malaysia AACTAACCAGGATTCCCCTAGTAATGGCGAACGAACAGGGAACAGCTCAAATTTTAAATCTCGGGCGCCTGCGTCCGCGAATTGTAGTTTCCAGAAACGTTATCCCCAGGAGGACCACCGGTCTAAGTTGCTTGGAACAGCATATCGGAGAGGGTGAGAATCCCGTTTTCGGCCGGAGGTTCCCTGGCACGAGGCGTTTTCCAAGAGTCGGGTTGTTTGGGATTGCAGCCCAAAAGGCGTGGTATACCCCACGTAAAGCTAAATATTGGCACGAGACCGATAGCGAACAAGTACCATGAGGGAAAGATGAAAAGCACTTTGAAAAGAGAGTTAAACAGCACGTGAAACCGTTAAAAGGGAAACGAATGGAGCCAGCAAGGCACCCAGTTTCATTCAGCCGGTGGCCTACCA-CCGAGGCCTGGGCCGACCTT--CGGGCGGTGCGGGTTTCGCGATGATAGGTTGCCGTGTGCACTTATGCTGGGTGCGTGCCAACTTGGGCTGGTGACGGTCGACACGGTCGGAAGGGAAGGTAGCTTTCTCCG-GGGAGTGTTATAGCCCTCCGTCCCGGGCCGTCGCTGGCTGAGGTGTCGCGGTACGTGCTCTCTTGGGCTTGGGCCCTGTAGCAGGTCGACTGCGCCTTCCGTTGACTGCACGCAGTGAGCGGAGGGACGACGGTAGGCTAGCCGTGGGTGTCCCGTAAACGTACCTAGGAAGTTG

KT885956_Coeloplana_sp_3_Malaysia AACTAACCAGGATTCCCCTAGTAATGGCGAACGAACAGGGAACAGCTCAAATTTTAAATCTCGGGCGCCTGCGTCCGCGAATTGTAGTTTCCAGAAACGTTATCCCCAGGAGGACCACCGGTCTAAGTTGCTTGGAACAGCATATCGGAGAGGGTGAGAATCCCGTTTTCGGCCGGAGGTTCCCTGGCACGAGGCGTTTTCCAAGAGTCGGGTTGTTTGGGATTGCAGCCCAAAAGGCGTGGTATACCCCACGTAAAGCTAAATATTGGCACGAGACCGATAGCGAACAAGTACCATGAGGGAAAGATGAAAAGCACTTTGAAAAGAGAGTTAAACAGCACGTGAAACCGTTAAAAGGGAAACGAATGGAGCCAGCAAGGCACCCAGTTTCATTCAGCCGGTGGCCTACCA-CCGAGGCCYGGGCCGACCTT--CGGGCGGTGCGGGTTTCGCGATGATAGGTTGCCGTGTGCACTTATGCTGGGTGCGTGCCAACTTGGGCTGGTGACGGTCGACACGGTCGGAAGGGAAGGTAGCTTTCTCCG-GGGAGTGTTATAGCCCTCCGTCCCGGGCCGTCGCTGGCTGAGGGGTCGCGGTACGTGCTCTCTTGGGCTTGGGCCCTGTAGCAGGTCGACTGCGCCTTCCGTTGACTGCACGCAGTGAGCGGAGGGACGACGGTAGGCTAGCCGTGGGTGTCCCGTAAACGTACCTAGGAAGTTG

KT885957_Coeloplana_fishelsoni_Red_Sea AACTAACCAGGATTCCCCTAGTAATGGCGAACGAACAGGGAACAGCTCAAATTTTAAATCTCGGGCGCCTGCGTCCGCGAATTGTAGTTTCCAGAAACGTTATCCCCAGGAGGACCACCGGTCTAAGTTGCTTGGAACAGCATATCGGAGAGGGTGAGAATCCCGTTTTCGGCCGGAGGTTCCCTGGCACGAGGCGTTTTCCAAGAGTCGGGTTGTTTGGGATTGCAGCCCAAAAGGCGTGGTATACCCCACGTAAAGCTAAATATTGGCACGAGACCGATAGCGAACAAGTACCATGAGGGAAAGATGAAAAGCACTTTGAAAAGAGAGTTAAACAGCACGTGAAACCGTTAAAAGGGAAACGAATGGAGCCAGCAAGGCACCCAGTTTCATTCAGCCGGTGGCCTACCA-CCAAGGCATGGGCCGACCTT--CGGGCGGTGCTTGTTTTGCGATGATAGGTTGCCGTGTGCACTTATGCTGGGTGCGTGCCAACTTGGGCTGGTGACGGTCGACACGGTCGGAAGGGAAGGTAGCTTTCTCCG-GGGAGTGTTATAGCCCTCCGTCCCGGGCCGTCGCTGGCTGAGGGGTCGCGGTACGTGCTCTCTTGGGCTTGGGCCCTGTAGCAGGTCGACTGCGCCTTCCGTTGACTGCACGCAGTGAGCGGAGGGACGACGGTAGGCTAGCCGTGGGTGTCCCGTAAACGTACCTAGGAAGTTG

KT885958_Vallicula_multiformis_Red_Sea AACTAACCAGGATTCCCTTAGTAATGGCGAACGAACAGGGAAAAGCTCAAATTTTAAATCTCGGGCGCTTGCGTCCGCGAATTGTAGTTTCCAGAAACGTTATCCCCAGGATGGCCGCCGGTCTAAGTTGCTTGGAACAGCATATCGAAGAGGGTGAGAATCCCGTTTTCGGCCGGTGGCTCCCTGGCACGAGGCGTTTTCGAAGAGTCGGGTTGTTTGGGATTGCAGCCCAAAATGCGTGGTAAACCCCACGTAAAGCTAAATATTGGCACGAGACCGATAGCGAACAAGTACCATGAGGGAAAGATGAAAAGCACTTTGAAAAGAGAGTTAAACAGCACGTGAAACCGTTAAAAGGGAAACGAATGGAGCCAGCAAGGCATCCAGATTCATTCAGCCGGTGGCTGGCCAACTCGGGTTCGGGGTGACCTTTACGGGCGCTCCGGGTTCGGCGATGACCCGTTGCCGTGTGCACTTATTCTGGGTGTGAGCCAACTTGGGCTGGAGACGGTTGACACGGTTCGGTGGGAAGGTAGCTCCCTCCGTGGGAGTATTATAGCCCCCGATCCTGAATCGTCCTCGGCTGAGGAGTTGCGGTACGTGCTCTCTCGGGCTTGGGCCCTGTGGGCGGTCGGTCGCGCCTTCTGTTGACTGCACGCAGTGAGCGGAGGGACGACGGCAGGCTTCCCGTGGGTGTCCCGTAAACGTACCCAGGAAGTTG

;

end;

**ITS1 nucleotide alignment. DNA sequence alignment, in Nexus format, used to reconstruct the phylogenetic tree presented in Figure 3**

#NEXUS

Begin data;

Dimensions ntax=15 nchar=392;

Format datatype=DNA gap=- missing=? matchchar=.;

Matrix

KT8855959_Coeloplana_bannwarthi_Red_Sea AACCTGCGGAAGGATCATTAACGAATCCCAATCTATA-CTTGCCTTAGCGGTTGTAACTTGAAGCTGTCCTTCGGGGTCATGG-----------GAAGGGGGCAGATCGGGAGCTTTAAAACTC--------CCGAT--CGCTCTCCCCCATGTTAAATCGTCAGTGGGTAAACCG----------GCGTGCGTG------------------------------CACGCTGGGGCGGGCCCCGAATTAGTTTTTTCTTTCACCCACGCTCCTTTTCTAACTACCTAAAC-AAACGTTGCTAATCCTCTTGACATGTAT---TATAYACACGTCGAGGGAGTATGCCTTTAATAGAGAACAACTTTTAACGGTGGATCTCTTGGCTCGTGCA

KT8855960_Coeloplana_lineolata_Red_Sea AACCTGCGGAAGGATCATTAACGAATCCCAATCTATA-CTTGCCTTAGCGGTTGTAACTTGAAGCTGTCCTTCGGGGTCATGG-----------GAAGGGGGCAGATCGGGAGCTTTAAAACTC--------CCGAT--CGCTCTCCCCCATGTTAAATCGTCAGTGGGTAAACCG----------GCGTGCGTG------------------------------CACGCTGGGGCGGGCCCCGAATTAGTTTTTTCTTTCACCCACGCTCCTTTTCTAACTRCCTAAAC-AAACGTTGCTAATCCTCTTGACATGTAT---TATAYACACGTCGAGGGAGTATGCCTTTAATAGAGAACAACTTTTAACGGTGGATCTCTTGGCTCGTGCA

KT8855961_Coeloplana_fishelsoni_Red_Sea AACCTGCGGAAGGATCATTAACGAATCCCAATCTATA-CTTGCCTTAGCGGTTGTAACTTGAAGCTGTCCTTCGGGGTCATGG-----------GAAGGGGGCAGATCGGGAGCTTTAAAACTC--------CCGAT--CGCTCTCCCCCATGTTAAATCGTCAGTGGGTAAACCG----------GCGTGCGTG------------------------------CACGCTGGGGCGGGCCCCGAATTAGTTTTTTCTTTCACCCACGCTCCTTTTCTAACTACCTAAAC-AAACGTTGCTAATCCTCTTGACATGTAT---TATATACACGTCGAGGGAGTATGCCTTTAATAGAGAACAACTTTTAACGGTGGATCTCTTGGCTCGTGCA

AF293683_Coeloplana_bannwarthi AACCTGCGGAAGGATCATTAACGAATYCCAATCTATA-CTTGCCTTAGCGGTTGTAACTTGAAGCTGTCCTTCGGGGTCATGG-----------GAAGGGGGCAGATCGGGAGCTTTAAAACTC--------CCGAT--CGCTCTCCCCCATGTTAAATCGTCAGTGGGTAAACCG----------GTGTGCGTG------------------------------CACGCTGGGGCGGGCCCCGAATTAGTTTTTTCTTTCACCCACGCTCCTTTTCTAACTACCTAAAC-AAACGTTGCTAATCCTCTTGACGTGTAT---TATACACGTCGAGGGAGAGTATGCCTTTAATAGAGAACAACTTTTAACGGTGGATCTCTTGGCTCGTGCA

KT8855962_Coeloplana_bannwarthi_var_Red_Sea AACCTGCGGAAGGATCATTAACGAATCCCAATCTATA-CTTGCCTTAGCGGTTGTAACTTGAAGCTGTCCTTCGGGGTCATGG-----------GAAGGGGGCAGATCGGGAGCTTTAAAACTC--------CCGAT--CGCTCTCCCCCATGTTAAATCGTCAGTGGGTAAACCG----------GCGTGCGTG------------------------------CACGCTGGGGCGGGCCCCGAATTAGTTTTTTCTTTCACCCACGCTCCTTTTCTAACTACCTAAAC-AAACGTTGCTAATCCTCTTGACGTGTAT---TATACACGTCGAGGGGGAGTATGCCTTTAATAGAGAACAACTTTTAACGGTGGATCTCTTGGCTCGTGCA

KT8855963_Coeloplana_fishelsoni_var_Red_Sea AACCTGCGGAAGGATCATTAACGAATCCCAATCTATA-CTTGCCTTAGCGGTTGTAACTTGAAGCTGTCCTTCGGGGTCATGG-----------GAAGGGA--AGACCGGGA--CTTAAAACTC--------CTGTT----CTCTCTCTCATGTTAAATCGTCAGTGGGTAAACCG----------GCGTGCATG------------------------------CACGCTGGGGCGGGCCCCGAATTAGTTTTTTCTTTCACCCACGCTCCTTTTCTAACTACCTAAAC-AAACGTTGCTAATCCTCTTGATGTGTATACTTATACACGTCGAGGGAGAGTATGCCTTTAATAGAGAACAACTTTTAACGGTGGATCTCTTGGCTCGTGCA

KT8855964_Coeloplana_huchonae_Red_Sea AACCTGCGGAAGGATCATTAACGAATCCCAATCTATA-CTTGCCTTAGCGGTTGTAACTTGAAGCTGTCCTTCGGGGTCATGG-----------GAAGGGA--AGACCGGGA--CTTAAAACTC--------CTGTT----CTCTCTCTCATGTTAAATCGTCAGTGGGTAAACCG----------GCGTGCATG------------------------------CACGCTGGGGCGGGCCCCGAATTAGTTTTTTCTTTCACCCACGCTCCTTTTCTAACTACCTAAACTGAACGTTGCTAATCCTCTTGAKGTGTATACYTATACACGTCGAGGGAGAGTATGCCTTTAATAGAGAACAACTTTTAACGGTGGATATCTTGTGTCTTGCA

HQ435812_Coeloplana_anthostella --------------------ACGAATCCCAATCTATA-CTTGCCTTAGCGGTTGTAACTTGAAGCTGTCCTTCGGGGTCATGG-----------GAAGGGA--AGACCGGGA--CTTAAAACTC--------CTGTT----CTCTCTCTCATGTTAAATCGTCAGTGGGTAAACCG----------GCGTGCATG------------------------------CACGCTGGGGCGGGCCCCGAATTAGTTTTTTCTTTCACCCACGCTCCTTTTCTAACTACCTAAACTAAACGTTGCTAATCCTCTTGATGTGTATACTTATACACGTCGAGGGAGAGTATGCCTTTAATAGAGAA---------------------------------

KT8855965_Coeloplana_yulianicorum_Red_Sea AACCTGCGGAAGGATCATTAACGAATCCCAATCTATA-CTTGCCTTAGCGGTTGTAACTTGAAGCTGTCCTTCGGGGTCATGG-----------GAAGGGAAGAAGACCGGG-CCTTAAAACTC--------CCGTTCTCTCTCTCTCCCATGTTAAATCGTCAGTGGGTAAACCG----------GTGTGCATG------------------------------CACGCTGGGGCGGGCCCCGAATTAGTTTTTTCTTTCACCCAC-----TTTTCTAACTACCTAAAC-AAACGTTGCTAATCCTCTTGACGTGTATGTAATACAACACGTCGAGGGAGTATGCCTTTAATAGAGAACAACTTTTAACGGTGGATCTCTTGGCTCGTGCA

KT8855966_Coeloplana_punctata_Red_Sea AACCTGCGGAAGGATCATTAACGAATCCCAATCTATA-CTTGCCTTAGCGGTTGTAACTTGAAGCTGTCCTTCGGGGTCATGG-----------GAAGACCGAGG----------------------------CATTGTCTCGCTCTCCCATGTTAAATCGTCAGTGGGTAAACCG----------GCGTGCGTGTGTGTGCAAGCACACACACACACACACACACACGCTGGGACGGGCCCCGAATTAGTTTTTTTTTTCACCCAC----TTTTTCTAATTACCTAAAC-AAACRTTGCTAATCCTCTTGACAT------------ACATGTCGAGGGAGTATGCCTTTAATAGAGAACAACTTTTAACGGTGGATCTCTTGGCTCGTGCA

KT8855967_Coeloplana_astericola_Malaysia AACCTGCGGAAGGATCATTAACGAATCCCAATCTATA-CTTGCCTTAGCGGTTGTAACTTGAAGCTGTCCTTCGGGGTCATGG-----------GAAGACCGAGG----------------------------CATTGTCTCGCTCTCCCATGTTAAATCGTCAGTGGGTAAACCG----------GCGTGCGTGTGTGTGCAAG------------CACCCGCACACGCTGGGACGGGCCCCGAATTAGTTTTTTTTT-CACCCAC----TTTTTCTAACTACCTAAAC-AAACGTTGCTAATCCTCTTGACAT------------ACATGTCGAGGGAGTATGCCTTTAATAGAGAACAACTTTTAACGGTGGATCTCTTGGCTCGTGCA

KT8855968_Coeloplana_sp_3_Malaysia AACCTGCGGAAGGATCATTAACGAATCCCAATCTATA-CTTGCCTTAGCGGTTGTAACTTGAAGCTGTCCTTCGGGGTCATGG-----------GAAGACCGAGA----------------------------CATTGTCTCGCTCTCCCATGTTAAATCGTCAGTGGGTAAACCG----------GCGTGCGTGTGTG--CAAG------------CACACACACACGCTGGGACGGGCCCCGAATTAGTTTTTTTTTTCACCCAC------TTTCTAACTACCTAAAC-AAACGTTGCTAATCCTCTTGACGT--------------ATGTCGAGGGAGTATGCCTTTAATAGAGAACAACTTTTAACGGTGGATCTCTTGGCTCGTGCA

HQ435814_Coeloplana_bocki --------------------ACGAATCCCAATCTATA-CTTGCCTTTGCGGTTGTAACTTGAAGCTGTCCTTCGGGGTCATGG-----------GGGGGGGG----CCGGGTGAGTTGAGCCTCGCGCTCAACCCTCTCCCCCTCCCTCCATGTTAAATCGTCAGTGGGTAAACCG----------GCGTGCA--------------------------------AACGCTGGGGCGGGCCCCGAATTAGTTTTTTCTTTCACCCACGCCTATTTTCTAACTGCCTAAA--AAACGTTGCCGATCCCCCTTGACTATAT--------ACACGTCGAGGGAGTATGCCTTTAATAGAGAA---------------------------------

AF293684_Vallicula_multiformis AACCTGCGGAAGGATCATTAACGAATCCAAAATCTTATCCTGCCTTTGCGGTTA-AACCATACGCTGTTTTTCGGGGTCGTTGCTCTCGCTCTCGAGAGAGGGGGAGTTGCA--------------------------------------GCGTTAAATTGCCAGTGGGTAACTGGATCATGGTGTGTGTGTGTGCGTAT-------TATGCGCGCGCCCCCCCACGGTCCGGGGTAAGCCCCGAATTCGTTTTTTTACCCAC-------GATTTTTAAATTGCCTTAAA-AAACA-TGTTAATAATCCCTACACACACACGTACGTGTGCGATGTGGGAGTATGCC--TAATAGAGAACAACTTTTAACGGTGGATCTCTTGGCTCGTGCA

KT8855969_Vallicula_multiformis_Red_Sea AACCTGCGGAAGGATCATTAACGAATCCAAAATCTTATCCTGCCTTTGCGGTTA-AACCATACGCTGTTTTTCGGGGTCGTTGCTCTCGCTCTCGAGAGAGGGGGAGTTGCA--------------------------------------GCGTTAAATTGCCAGTGGGTAACTGGATCATG----GTGTGTGTGCGTAT-------TATGCGCG----CCCCCACGGTCCGGGGCAAGCCCCGAATTCGTTTTTTTACCCAC-------GATTTTTAAATTGCCTTAAA-AAACA-TGTTAATAATCCCTACACACAC----RYACGTGTGATGTGGGAGTATGCC--TAATAGAGAACAACTTTTAACGGTGGATCTCTTGGCTCGTGCA

;

end;

**COI nucleotide alignment. DNA sequence alignment, in Nexus format, used to reconstruct the phylogenetic tree presented in Figure 4**

#NEXUS

Begin data;

Dimensions ntax=60 nchar=657;

Format datatype=DNA gap=- missing=? matchchar=.;

Matrix

KT885970_Coeloplana_brown_dots_Red_Sea TCTTTATATTTTTGATTTTCTATTTTTGTAGCCTTTATTGCTTTTAGTTATTCTTTTATTATTAGGTTATCTTTAATGTGGCCTTATTCTTTTTTAGTCGATGGAAACATTTACAACAGTTTTGTTTCTCTTCATGCCATTTTTATGATTTTTTTTTTTGTAATGCCTTTTTCCATTGGAGGTTTAGGTAATTGACTTATCCCTCTTTATATTGGAGCAGTAGATATGGCTCTTCCTAGAGTTAATAATTTATCCTTTTGGTTATTATTTGCAGCTTTTATCTTAGGTTTTTTTTCTTCAGTCTTTGCTATGGGTATTCATGCTGGTTGAACTATTTATCCTCCTTTATCCTCTTATATTGGCAGTCCCAACATTTCAACTGATTTTATAATTTTTTCTCTCCATTGTGCAGGTGCTAGTTCTATTTTAGCTTCTATTAACTTTTTTATTACTGTTTTTTTCTTAACTTCTGATGAAGAAGTTTTAAATTTTTTAAAATATCCACTTTTTATTATTGGTCAACTTGTAGTGGCTATTCTACTAATTTTGACTCTTCCTGTTTTGGCCGCAGCTATTACTATGCTCTTATTTGATAGAAATTTCAATTCTTGTTTTTTTTCTAATTGAGATGGTGGTGATGTTGTTTTATTTCAACAT

KT885971_Coeloplana_brown_dots_Red_Sea TCTTTATATTTTTGATTTTCTATTTTTGTAGCCTTTATTGCTTTTAGTTATTCTTTTATTATTAGGTTATCTTTAATGTGGCCTTATTCTTTTTTAGTCGATGGAAACATTTACAACAGTTTTGTTTCTCTTCATGCCATTTTTATGATTTTTTTTTTTGTAATGCCTTTTTCCATTGGAGGTTTAGGTAATTGACTTATCCCTCTTTATATTGGAGCAGTAGATATGGCTCTTCCTAGAGTTAATAATTTATCCTTTTGGTTATTATTTGCAGCTTTTATCTTAGGTTTTTTTTCTTCAGTCTTTGCTATGGGTATTCATGCTGGTTGAACTATTTATCCTCCTTTATCCTCTTATATTGGCAGTCCCAACATTTCAACTGATTTTATAATTTTTTCTCTCCATTGTGCAGGTGCTAGTTCTATTTTAGCTTCTATTAACTTTTTTATTACTGTTTTTTTCTTAACTTCTGATGAAGAAGTTTTAAATTTTTTAAAATATCCACTTTTTATTATTGGTCAACTTGTAGTGGCTATTCTACTAATTTTGACTCTTCCTGTTTTGGCCGCAGCTATTACTATGCTCTTATTTGATAGAAATTTCAATTCTTGTTTTTTTTCTAATTGAGATGGTGGTGATGTTGTTTTATTTCAACAT

KT885972_Coeloplana_brown_dots_Red_Sea TCTTTATATTTTTGATTTTCTATTTTTGTAGCCTTTATTGCTTTTAGTTATTCTTTTATTATTAGGTTATCTTTAATGTGGCCTTATTCTTTTTTAGTCGATGGAAACATTTACAACAGTTTTGTTTCTCTTCATGCCATTTTTATGATTTTTTTTTTTGTAATGCCTTTTTCCATTGGAGGTTTAGGTAATTGACTTATCCCTCTTTATATTGGAGCAGTAGATATGGCTCTTCCTAGAGTTAATAATTTATCCTTTTGGTTATTATTTGCAGCTTTTATCTTAGGTTTTTTTTCTTCAGTCTTTGCTATGGGTATTCATGCTGGTTGAACTATTTATCCTCCTTTATCCTCTTATATTGGCAGTCCCAACATTTCAACTGATTTTATAATTTTTTCTCTCCATTGTGCAGGTGCTAGTTCTATTTTAGCTTCTATTAACTTTTTTATTACTGTTTTTTTCTTAACTTCTGATGAAGAAGTTTTAAATTTTTTAAAATATCCACTTTTTATTATTGGTCAACTTGTAGTGGCTATTCTACTAATTTTGACTCTTCCTGTTTTGGCCGCAGCTATTACTATGCTCTTATTTGATAGAAATTTCAATTCTTGTTTTTTTTCTAATTGAGATGGTGGTGATGTTGTTTTATTTCAACAT

KT885973_Coeloplana_brown_dots_Red_Sea TCTTTATATTTTTGATTTTCTATTTTTGTAGCCTTTATTGCTTTTAGTTATTCTTTTATTATTAGGTTATCTTTAATGTGGCCTTATTCTTTTTTAGTCGATGGAAACATTTACAACAGTTTTGTTTCTCTTCATGCCATTTTTATGATTTTTTTTTTTGTAATGCCTTTTTCCATTGGAGGTTTAGGTAATTGACTTATCCCTCTTTATATTGGAGCAGTAGATATGGCTCTTCCTAGAGTTAATAATTTATCCTTTTGGTTATTATTTGCAGCTTTTATCTTAGGTTTTTTTTCTTCAGTCTTTGCTATGGGTATTCATGCTGGTTGAACTATTTATCCTCCTTTATCCTCTTATATTGGCAGTCCCAACATTTCAACTGATTTTATAATTTTTTCTCTCCATTGTGCAGGTGCTAGTTCTATTTTAGCTTCTATTAACTTTTTTATTACTGTTTTTTTCTTAACTTCTGATGAAGAAGTTTTAAATTTTTTAAAATATCCACTTTTTATTATTGGTCAACTTGTAGTGGCTATTCTACTAATTTTGACTCTTCCTGTTTTGGCTGCAGCTATTACTATGCTCTTATTTGATAGAAATTTCAATTCTTGTTTTTTTTCTAATTGAGATGGTGGTGATGTTGTTTTATTTCAACAT

KT885974_Coeloplana_fishelsoni_Red_Sea TCTTTATATTTTTGATTTTCTATTTTTGTAGCCTTTATTGCTTTTAGTTATTCTTTTATTATTAGGTTATCTTTAATGTGGCCTTATTCTTTTTTAGTCGATGGAAACATTTACAACAGTTTTGTTTCTCTTCATGCCATTTTTATGATTTTTTTTTTTGTGATGCCTTTTTCCATTGGAGGTTTAGGTAATTGACTTATTCCTCTTTATATTGGAGCAGTAGATATGGCTCTCCCTAGAGTTAATAATTTATCCTTTTGGTTATTATTTGCAGCTTTTATCTTAGGTTTTTTTTCTTCAGTCTTTGCTATGGGGATTCACGCTGGTTGAACTATTTACCCTCCACTATCTTCTTATATTGGTAGTCCTAATATTTCAACTGATTTTATAATTTTTTCTCTCCATTGTGCAGGTGCTAGTTCTATTTTAGCTTCTATTAACTTCTTCATTACTGTTTTTTTTTTAACTTCTGACGAAGAAGTTTTAAATTTTTTGAAGTATCCACTTTTTATTATTGGTCAACTTGTAGTAGCTATTCTACTAATTTTAACTCTTCCTGTCTTGGCCGCAGCTATTACTATGCTTTTATTTGATAGAAATTTTAATTCTTGTTTTTTTTCTAATTGAGATGGTGGTGATGTTGTTTTATTTCAACAT

KT885975_Coeloplana_fishelsoni_Red_Sea TCTTTATATTTTTGATTTTCTATTTTTGTAGCCTTTATTGCTTTTAGTTATTCTTTTATTATTAGGTTATCTTTAATGTGGCCTTATTCTTTTTTAGTCGATGGAAACATTTACAACAGTTTTGTTTCTCTTCATGCCATTTTTATGATTTTTTTTTTTGTGATGCCTTTTTCCATTGGAGGTTTAGGCAATTGACTTATTCCTCTTTATATTGGAGCAGTAGATATGGCTCTCCCTAGAGTTAATAATTTATCCTTTTGGTTATTATTTGCAGCTTTTATTTTAGGTTTTTTTTCTTCAGTCTTTGCTATGGGGATTCACGCTGGTTGAACTATTTACCCTCCACTATCTTCTTATATTGGTAGTCCTAATATTTCAACTGATTTTATAATTTTTTCTCTCCATTGTGCAGGTGCTAGTTCTATTTTAGCTTCTATTAACTTCTTCATTACTGTTTTTTTCTTAACTTCTGACGAAGAAGTTTTAAATTTTTTGAAGTATCCACTTTTTATTATTGGTCAACTTGTAGTAGCTATTCTACTAATTTTAACTCTTCCTGTCTTGGCCGCAGCTATTACTATGCTTTTATTTGATAGAAATTTTAATTCTTGTTTTTTTTCTAATTGAGATGGTGGTGATGTTGTTTTATTTCAACAT

KT885976_Coeloplana_fishelsoni_var_Red_Sea TCTTTATATTTTTGATTTTCTATTTTTGTGGCTTTTATTGCTTTTAGTTATTCTTTTATTATAAGGTTATCTCTAATGTGACCTTATTCTTTTTTAGTTGATGGTAACATTTACAATAGTTTTGTTTCTCTTCATGCTATTTTTATGATTTTTTTCTTTGTTATGCCTTTTTCTATTGGAGGTTTAGGTAATTGACTCATTCCTCTTTATATTGGAGCTGTAGACATGGCTCTTCCCAGAGTTAACAATTTATCTTTTTGGTTATTATTTGCAGCTTTTATTTTAGGTTTTTTTTCTTCAGTCTTTGCTATGGGTATACATGCTGGTTGAACTATTTATCCCCCTCTATCCTCTTATATAGGTAGTCCTAACATCTCAACTGATTTTATAATTTTTTCTCTTCATTGTGCAGGTGCTAGCTCTATTTTAGCTTCTATTAATTTTTTTATTACTGTTTTTTTTTTAACTTCTGATGAAGAAGTTTTAAATTTCTTAAAGTATCCACTTTTTATTATTGGTCAACTTGTAGTGGCTATTTTATTAATTTTAACCCTTCCTGTCTTGGCCGCAGCTATTACTATGTTGTTATTTGATAGAAACTTTAATTCTTGTTTTTTTTCTAATTGAGACGGAGGTGATGTTGTTTTATTCCAACAT

KT885977_Coeloplana_fishelsoni_Red_Sea TCTTTATATTTTTGATTTTCTATTTTTGTAGCCTTTATTGCTTTTAGTTATTCTTTTATTATTAGGTTATCTTTAATGTGGCCTTATTCTTTTTTAGTCGATGGAAACATTTACAACAGTTTTGTTTCTCTTCATGCCATTTTTATGATTTTTTTTTTTGTGATGCCTTTTTCCATTGGAGGTTTAGGCAATTGACTTATTCCTCTTTATATTGGAGCAGTAGATATGGCTCTCCCTAGAGTTAATAATTTATCCTTTTGGTTATTATTTGCAGCTTTTATCTTAGGTTTTTTTTCTTCAGTCTTTGCTATGGGGATTCACGCTGGTTGAACTATTTACCCTCCACTATCTTCTTATATTGGTAGTCCTAATATTTCAACTGATTTTATAATTTTTTCTCTCCATTGTGCAGGTGCTAGTTCTATTTTAGCTTCTATTAACTTCTTCATTACTGTTTTTTTCTTAACTTCTGACGAAGAAGTTTTAAATTTTTTGAAGTATCCACTTTTTATTATTGGTCAACTTGTAGTAGCTATTCTACTAATTTTAACTCTTCCTGTCTTGGCCGCAGCTATTACTATGCTTTTATTTGATAGAAATTTTAATTCTTGTTTTTTTTCTAATTGAGATGGTGGTGATGTTGTTTTATTTCAACAT

KT885978_Coeloplana_fishelsoni_Red_Sea TCTTTATATTTTTGATTTTCTATTTTTGTAGCCTTTATTGCTTTTAGTTATTCTTTTATTATTAGGTTATCTTTAATGTGGCCTTATTCTTTTTTAGTCGATGGAAACATTTACAACAGTTTTGTTTCTCTTCATGCCATTTTTATGATTTTTTTTTTTGTGATGCCTTTTTCCATTGGAGGTTTAGGCAATTGACTTATTCCTCTTTATATTGGAGCAGTAGATATGGCTCTCCCTAGAGTTAATAATTTATCCTTTTGGTTATTATTTGCAGCTTTTATCTTAGGTTTTTTTTCTTCAGTCTTTGCTATGGGGATTCACGCTGGTTGAACTATTTACCCTCCACTATCTTCTTATATTGGTAGTCCTAATATTTCAACTGATTTTATAATTTTTTCTCTCCATTGTGCAGGTGCTAGTTCTATTTTAGCTTCTATTAACTTCTTCATTACTGTTTTTTTCTTAACTTCTGACGAAGAAGTTTTAAATTTTTTGAAGTATCCACTTTTTATTATTGGTCAACTTGTAGTAGCTATTCTACTAATTTTAACTCTTCCTGTCTTGGCCGCAGCTATTACTATGCTTTTATTTGATAGAAATTTTAATTCTTGTTTTTTTTCTAATTGAGATGGTGGTGATGTTGTTTTATTTCAACAT

KT885979_Coeloplana_fishelsoni_Red_Sea TCTTTATATTTTTGATTTTCTATTTTTGTAGCCTTTATTGCTTTTAGTTATTCTTTTATTATTAGGTTATCTTTAATGTGGCCTTATTCTTTTTTAGTCGATGGAAACATTTACAACAGTTTTGTTTCTCTTCATGCCATTTTTATGATTTTTTTTTTTGTGATGCCTTTTTCCATTGGAGGTTTAGGCAATTGACTTATTCCTCTTTATATTGGAGCAGTAGATATGGCTCTCCCTAGAGTTAATAATTTATCCTTTTGGTTATTATTTGCAGCTTTTATCTTAGGTTTTTTTTCTTCAGTCTTTGCTATGGGGATTCACGCTGGTTGAACTATTTACCCTCCACTATCTTCTTATATTGGTAGTCCTAATATTTCAACTGATTTTATAATTTTTTCTCTCCATTGTGCAGGTGCTAGTTCTATTTTAGCTTCTATTAACTTCTTCATTACTGTTTTTTTCTTAACTTCTGACGAAGAAGTTTTAAATTTTTTGAAGTATCCACTTTTTATTATTGGTCAACTTGTAGTAGCTATTCTACTAATTTTAACTCTTCCTGTCTTGGCCGCAGCTATTACTATGCTTTTATTTGATAGAAATTTTAATTCTTGTTTTTTTTCTAATTGAGATGGTGGTGATGTTGTTTTATTTCAACAT

KT885980_Coeloplana_lineolata_Red_Sea TCTTTATATTTTTGATTTTCTATTTTTGTAGCCTTTATTGCTTTTAGTTATTCTTTTATTATTAGGTTATCTTTAATGTGGCCTTATTCTTTTTTAGTCGATGGAAACATTTACAACAGTTTTGTTTCTCTTCATGCCATTTTTATGATTTTTTTTTTTGTAATGCCTTTTTCCATTGGAGGTTTAGGTAATTGACTTATCCCTCTTTATATTGGAGCAGTAGATATGGCTCTTCCTAGAGTTAATAATTTATCCTTTTGGTTATTATTTGCAGCTTTTATCTTAGGTTTTTTTTCTTCAGTCTTTGCTATGGGTATTCATGCTGGTTGAACTATTTATCCTCCTTTATCCTCTTATATTGGCAGTCCCAACATTTCAACTGATTTTATAATTTTTTCTCTCCATTGTGCAGGTGCTAGTTCTATTTTAGCTTCTATTAACTTTTTTATTACTGTTTTTTTCTTAACTTCTGATGAAGAAGTTTTAAATTTTTTAAAATATCCACTTTTTATTATTGGTCAACTTGTAGTGGCTATTCTACTAATTTTGACTCTTCCTGTTTTGGCCGCAGCTATTACTATGCTCTTATTTGATAGAAATTTCAATTCTTGTTTTTTTTCTAATTGAGATGGTGGTGATGTTGTTTTATTTCAACAT

KT885981_Coeloplana_lineolata_Red_Sea TCTTTATATTTTTGATTTTCTATTTTTGTAGCCTTTATTGCTTTTAGTTATTCTTTTATTATTAGGTTATCTTTAATGTGGCCTTATTCTTTTTTAGTCGATGGAAACATTTACAACAGTTTTGTTTCTCTTCATGCCATTTTTATGATTTTTTTTTTTGTAATGCCTTTTTCCATTGGAGGTTTAGGTAATTGACTTATCCCTCTTTATATTGGAGCAGTAGATATGGCTCTTCCTAGAGTTAATAATTTATCCTTTTGGTTATTATTTGCAGCTTTTATCTTAGGTTTTTTTTCTTCAGTCTTTGCTATGGGTATTCATGCTGGTTGAACTATTTATCCTCCTTTATCCTCTTATATTGGCAGTCCCAACATTTCAACTGATTTTATAATTTTTTCTCTCCATTGTGCAGGTGCTAGTTCTATTTTAGCTTCTATTAACTTTTTTATTACTGTTTTTTTCTTAACTTCTGATGAAGAAGTTTTAAATTTTTTAAAATATCCACTTTTTATTATTGGTCAACTTGTAGTGGCTATTCTACTAATTTTGACTCTTCCTGTTTTGGCCGCAGCTATTACTATGCTCTTATTTGATAGAAATTTCAATTCTTGTTTTTTTTCTAATTGAGATGGTGGTGATGTTGTTTTATTTCAACAT

KT885982_Coeloplana_linoelata_Red_Sea TCTTTATATTTTTGATTTTCTATTTTTGTAGCCTTTATTGCTTTTAGTTATTCTTTTATTATTAGGTTATCTTTAATGTGGCCTTATTCTTTTTTAGTCGATGGAAACATTTACAACAGTTTTGTTTCTCTTCATGCCATTTTTATGATTTTTTTTTTTGTAATGCCTTTTTCCATTGGAGGTTTAGGTAATTGACTTATCCCTCTTTATATTGGAGCAGTAGATATGGCTCTTCCTAGAGTTAATAATTTATCCTTTTGGTTATTATTTGCAGCTTTTATCTTAGGTTTTTTTTCTTCAGTCTTTGCTATGGGTATTCATGCTGGTTGAACTATTTATCCTCCTTTATCCTCTTATATTGGCAGTCCCAACATTTCAACTGATTTTATAATTTTTTCTCTCCATTGTGCAGGTGCTAGTTCTATTTTAGCTTCTATTAACTTTTTTATTACTGTTTTTTTCTTAACTTCTGATGAAGAAGTTTTAAATTTTTTAAAATATCCACTTTTTATTATTGGTCAACTTGTAGTGGCTATTCTACTAATTTTGACTCTTCCTGTTTTGGCCGCAGCTATTACTATGCTCTTATTTGATAGAAATTTCAATTCTTGTTTTTTTTCTAATTGAGATGGTGGTGATGTTGTTTTATTTCAACAT

KT885983_Coeloplana_punctata_Red_Sea TCTTTATATTTTTGATTTTCTATTTTTGTAGCCTTTATTGCTTTTAGTTATTCTTTTATTATTAGGTTATCTTTAATGTGGCCTTATTCTTTTTTAGTCGATGGAAACATTTACAACAGTTTTGTTTCTCTTCATGCCATTTTTATGATTTTTTTTTTTGTAATGCCTTTTTCCATTGGAGGTTTAGGTAATTGACTTATCCCTCTTTATATTGGAGCAGTAGATATGGCTCTTCCTAGAGTTAATAATTTATCCTTTTGGTTATTATTTGCAGCTTTTATCTTAGGTTTTTTTTCTTCAGTCTTTGCTATGGGTATTCATGCTGGTTGAACTATTTATCCTCCTTTATCCTCTTATATTGGCAGTCCCAACATTTCAACTGATTTTATAATTTTTTCTCTCCATTGTGCAGGTGCTAGTTCTATTTTAGCTTCTATTAACTTTTTTATTACTGTTTTTTTCTTAACTTCTGATGAAGAAGTTTTAAATTTTTTAAAATATCCACTTTTTATTATTGGTCAACTTGTAGTGGCTATTCTACTAATTTTGACTCTTCCTGTTTTGGCTGCAGCTATTACTATGCTCTTATTTGATAGAAATTTCAATTCTTGTTTTTTTTCTAATTGAGATGGTGGTGATGTTGTTTTATTTCAACAT

KT885984_Coeloplana_punctata_Red_Sea TCTTTATATTTTTGATTTTCTATTTTTGTAGCCTTTATTGCTTTTAGTTATTCTTTTATTATTAGGTTATCTTTAATGTGGCCTTATTCTTTTTTAGTCGATGGAAACATTTACAACAGTTTTGTTTCTCTTCATGCCATTTTTATGATTTTTTTTTTTGTAATGCCTTTTTCCATTGGAGGTTTAGGTAATTGACTTATCCCTCTTTATATTGGAGCAGTAGATATGGCTCTTCCTAGAGTTAATAATTTATCCTTTTGGTTATTATTTGCAGCTTTTATCTTAGGTTTTTTTTCTTCAGTCTTTGCTATGGGTATTCATGCTGGTTGAACTATTTATCCTCCTTTATCCTCTTATATTGGCAGTCCCAACATTTCAACTGATTTTATAATTTTTTCTCTCCATTGTGCAGGTGCTAGTTCTATTTTAGCTTCTATTAACTTTTTTATTACTGTTTTTTTCTTAACTTCTGATGAAGAAGTTTTAAATTTTTTAAAATATCCACTTTTTATTATTGGTCAACTTGTAGTGGCTATTCTACTAATTTTGACTCTTCCTGTTTTGGCTGCAGCTATTACTATGCTCTTATTTGATAGAAATTTCAATTCTTGTTTTTTTTCTAATTGAGATGGTGGTGATGTTGTTTTATTTCAACAT

KT885985_Coeloplana_punctata_Red_Sea TCTTTATATTTTTGATTTTCTATTTTTGTAGCCTTTATTGCTTTTAGTTATTCTTTTATTATTAGGTTATCTTTAATGTGGCCTTATTCTTTTTTAGTCGATGGAAACATTTACAACAGTTTTGTTTCTCTTCATGCCATTTTTATGATTTTTTTTTTTGTAATGCCTTTTTCCATTGGAGGTTTAGGTAATTGACTTATCCCTCTTTATATTGGAGCAGTAGATATGGCTCTTCCTAGAGTTAATAATTTATCCTTTTGGTTATTATTTGCAGCTTTTATCTTAGGTTTTTTTTCTTCAGTCTTTGCTATGGGTATTCATGCTGGTTGAACTATTTATCCTCCTTTATCCTCTTATATTGGCAGTCCCAACATTTCAACTGATTTTATAATTTTTTCTCTCCATTGTGCAGGTGCTAGTTCTATTTTAGCTTCTATTAACTTTTTTATTACTGTTTTTTTCTTAACTTCTGATGAAGAAGTTTTAAATTTTTTAAAATATCCACTTTTTATTATTGGTCAACTTGTAGTGGCTATTCTACTAATTTTGACTCTTCCTGTTTTGGCTGCAGCTATTACTATGCTCTTATTTGATAGAAATTTCAATTCTTGTTTTTTTTCTAATTGAGATGGTGGTGATGTTGTTTTATTTCAACAT

KT885986_Coeloplana_yulianicorum_Red_Sea TCTTTATATTTTTGATTTTCTATTTTTGTAGCTTTCATTGCTTTTAGCTATTCTTTTATTATAAGATTATCCTTAATGTGACCCTACTCATTCTTAGTGGATGGAAACATCTACAATAGCTTTGTTTCTTTACATGCTATTTTTATGATTTTTTTTTTTGTTATGCCTTTCTCAATAGGGGGTCTAGGTAATTGACTTATTCCCCTTTACATTGGAGCTGTAGATATGGCTCTTCCTAGAGTTAACAATTTATCTTTTTGGTTATTGTTTGCAGCTTTTATATTGGGTTTCTTTTCTTCTGTGTTTGCCATGGGTATTCATGCAGGTTGAACCATCTATCCTCCTTTGTCTTCCTACATTGGAAGTCCTAACATTTCTACAGATTTTATAATTTTTTCACTTCACTGTGCAGGTGCTAGTTCAATTTTAGCTTCTATAAATTTTTTTATTACTGTTTTTTTTTTAACTTCTGATGAAGAAGTCCTAAATTTTTTAAAATATCCTCTTTTTATCATTGGTCAATTAGTGGTGGCCATTCTTTTAATCTTAACTCTTCCAGTACTGGCTGCAGCTATTACTATGTTATTATTTGACAGAAATTTTAATTCTTGTTTTTTTTCTAATTGAGATGGGGGAGATGTTGTTCTTTTTCAACAT

KT885987_Coeloplana_yulianicorum_Red_Sea TCTTTATATTTTTGATTTTCTATTTTTGTAGCTTTCATTGCTTTTAGCTATTCTTTTATTATAAGATTATCCTTAATGTGACCCTACTCATTCTTAGTGGATGGAAACATCTACAATAGCTTTGTTTCTTTACATGCTATTTTTATGATTTTTTTTTTTGTTATGCCTTTCTCAATAGGGGGTCTAGGTAATTGACTTATTCCCCTTTACATTGGAGCTGTAGATATGGCTCTTCCTAGAGTTAACAATTTATCTTTTTGGTTATTGTTTGCAGCTTTTATATTGGGTTTCTTTTCTTCTGTGTTTGCCATGGGTATTCATGCAGGTTGAACCATCTATCCTCCTTTGTCTTCCTACATTGGAAGTCCTAACATTTCTACAGATTTTATAATTTTTTCACTTCACTGTGCAGGTGCTAGTTCAATTTTAGCTTCTATAAATTTTTTTATTACTGTTTTTTTTTTAACTTCTGATGAAGAAGTCCTAAATTTTTTAAAATATCCTCTTTTTATCATTGGTCAATTAGTGGTGGCCATTCTTTTAATCTTAACTCTTCCAGTACTGGCTGCAGCTATTACTATGTTATTATTTGACAGAAATTTTAATTCTTGTTTTTTTTCTAATTGAGATGGGGGAGATGTTGTTCTTTTTCAACAT

KT885988_Coeloplana_yulianicorum_Red_Sea TCTTTATATTTTTGATTTTCTATTTTTGTAGCTTTCATTGCTTTTAGCTATTCTTTTATTATAAGATTATCCTTAATGTGACCCTACTCATTCTTAGTGGATGGAAACATCTACAATAGCTTTGTTTCTTTACATGCTATTTTTATGATTTTTTTTTTTGTTATGCCTTTCTCAATAGGGGGTCTAGGTAATTGACTTATTCCCCTTTACATTGGAGCTGTAGATATGGCTCTTCCTAGAGTTAACAATTTATCTTTTTGGTTATTGTTTGCAGCTTTTATATTGGGTTTCTTTTCTTCTGTGTTTGCCATGGGTATTCATGCAGGTTGAACCATCTATCCTCCTTTGTCTTCCTACATTGGAAGTCCTAACATTTCTACAGATTTTATAATTTTTTCACTTCACTGTGCAGGTGCTAGTTCAATTTTAGCTTCTATAAATTTTTTTATTACTGTTTTTTTTTTAACTTCTGATGAAGAAGTCCTAAATTTTTTAAAATATCCTCTTTTTATCATTGGTCAATTAGTGGTGGCCATTCTTTTAATCTTAACTCTTCCAGTACTGGCTGCAGCTATTACTATGTTATTATTTGACAGAAATTTTAATTCTTGTTTTTTTTCTAATTGAGATGGGGGAGATGTTGTTCTTTTTCAACAT

HQ435811_1_Coeloplana_sp_SHL_2011 TCTTTATATTTTTGATTTTCTATTTTTGTGGCTTTTATTGCTTTTAGTTATTCTTTTATTATAAGGTTGTCTTTAATGTGACCTTATTCCTTCTTAGTTGATGGTAACATTTATAACAGTTTTGTTTCTCTTCATGCTATTTTCATGATTTTTTTTTTTGTTATGCCTTTTTCTATAGGAGGTTTAGGTAATTGACTTATTCCTCTCTATATTGGAGCTGTAGACATGGCTCTTCCCAGAGTTAACAATTTATCTTTTTGGTTACTATTTGCAGCTTTTATTTTAGGTTTTTTTTCTTCAGTCTTTGCCATGGGTATTCACGCTGGTTGAACAATTTATCCTCCTTTATCCTCTTATATAGGTAGTCCTAACATTTCAACTGATTTTATAATTTTTTCTCTTCATTGTGCAGGTGCTAGCTCTATTTTAGCTTCTATTAATTTTTTTATTACTGTTTTTTTTTTAACTTCTGATGAAGAAGTTTTAAATTTTCTAAAGTATCCACTTTTTATTATTGGTCAACTTGTAGTGGCTATTTTATTAATTTTAACCCTTCCTGTCTTGGCTGCAGCTATTACTATGCTTTTATTTGATAGAAATTTTAATTCTTGTTTTTTTTCTAATTGAGACGGGGGTGATGTTGTTTTGTTCCAACAT

KT885989_Coeloplana__astericola_Malaysia TCTTTATATTTTTGATTTTCTATATTTGTAGCTTTTATTGCTTTTAGTTATTCTTTTATAATCAGGTTATCTTTAATGTGACCTTATTCTTTCTTGATTGACGGTAATATTTATAATAGTTTTGTGTCTCTTCATGCAATTTTTATGATCTTTTTTTTTGTTATGCCATTTTCCATTGGAGGTTTAGGAAACTGACTTATTCCTTTGTATATAGGTGCTGTTGATATGGCTTTGCCTAGAGTAAATAATTTGTCTTTTTGATTATTATTTGCAGCTTTTATTTTAGGTTTTTTTTCTTCTGTTTTTGCTATGGGTATTCATGCAGGGTGAACAATTTACCCCCCTTTGTCTTCTTATATTGGAAGTCCTAATATTTCTACTGATTTTATAATTTTTTCTTTGCATTGTGCAGGTGCTAGTTCTATTTTAGCTTCTATAAATTTTTTTATTACTGTTTTCTTTTTAACTTCTGATGAAGAGGTTTTAAATTTTTTGAAGTATCCTCTTTTTATTATAGGTCAATTAGTTGTAGCTATTTTACTAATATTAACTTTGCCAGTTCTAGCTGCTGCTATTACTATGTTGTTGTTCGATAGAAATTTTAATTCTTGTTTTTTCTCTAACTGAGATGGAGGTGATGTTGTTTTATTTCAGCAC

KT885990_Coeloplana_astericola_Malaysia TCTTTATATTTTTGATTTTCTATATTTGTAGCTTTTATTGCTTTTAGTTATTCTTTTATAATCAGGTTATCTTTAATGTGACCTTATTCTTTCTTGATTGACGGTAATATTTATAATAGTTTTGTGTCTCTTCATGCAATTTTTATGATCTTTTTTTTTGTTATGCCATTTTCCATTGGAGGTTTAGGAAACTGACTTATTCCTTTGTATATAGGTGCTGTTGATATGGCTTTGCCTAGAGTAAATAATTTGTCTTTTTGATTATTATTTGCAGCTTTTATTTTAGGTTTTTTTTCTTCTGTTTTTGCTATGGGTATTCATGCAGGGTGAACAATTTACCCCCCTTTGTCTTCTTATATTGGAAGTCCTAATATTTCTACTGATTTTATAATTTTTTCTTTGCATTGTGCAGGTGCTAGTTCTATTTTAGCTTCTATAAATTTTTTTATTACTGTTTTCTTTTTAACTTCTGATGAAGAGGTTTTAAATTTTTTGAAGTATCCTCTTTTTATTATAGGTCAATTAGTTGTAGCTATTTTACTAATATTAACTTTGCCAGTTCTAGCTGCTGCTATTACTATGTTGTTGTTCGATAGAAATTTTAATTCTTGTTTTTTCTCTAACTGAGATGGAGGTGATGTTGTTTTATTTCAGCAC

KT885991_Coeloplana_astericola_Malaysia TCTTTATATTTTTGATTTTCTATATTTGTAGCTTTTATTGCTTTTAGTTATTCTTTTATAATCAGGTTATCTTTAATGTGACCTTATTCTTTCTTGATTGACGGTAATATTTATAATAGTTTTGTGTCTCTTCATGCAATTTTTATGATCTTTTTTTTTGTTATGCCATTTTCCATTGGAGGTTTAGGAAACTGACTTATTCCTTTGTATATAGGTGCTGTTGATATGGCTTTGCCTAGAGTAAATAATTTGTCTTTTTGATTATTATTTGCAGCTTTTATTTTAGGTTTTTTTTCTTCTGTTTTTGCTATGGGTATTCATGCAGGGTGAACAATTTACCCCCCTTTGTCTTCTTATATTGGAAGTCCTAATATTTCTACTGATTTTATAATTTTTTCTTTGCATTGTGCAGGTGCTAGTTCTATTTTAGCTTCTATAAATTTTTTTATTACTGTTTTCTTTTTAACTTCTGATGAAGAGGTTTTAAATTTTTTGAAGTATCCTCTTTTTATTATAGGTCAATTAGTTGTAGCTATTTTACTAATATTAACTTTGCCAGTTCTAGCTGCTGCTATTACTATGTTGTTGTTCGATAGAAATTTTAATTCTTGTTTTTTCTCTAACTGAGATGGAGGTGATGTTGTTTTATTTCAGCAC

KT885992_Coeloplana_astericola_Malaysia TCTTTATATTTTTGATTTTCTATATTTGTAGCTTTTATTGCTTTTAGTTATTCTTTTATAATCAGGTTATCTTTAATGTGACCTTATTCTTTCTTGATTGACGGTAATATTTATAATAGTTTTGTGTCTCTTCATGCAATTTTTATGATCTTTTTTTTTGTTATGCCATTTTCCATTGGAGGTTTAGGAAACTGACTTATTCCTTTGTATATAGGTGCTGTTGATATGGCTTTGCCTAGAGTAAATAATTTGTCTTTTTGATTATTATTTGCAGCTTTTATTTTAGGTTTTTTTTCTTCTGTTTTTGCTATGGGTATTCATGCAGGGTGAACAATTTACCCCCCTTTGTCTTCTTATATTGGAAGTCCTAATATTTCTACTGATTTTATAATTTTTTCTTTGCATTGTGCAGGTGCTAGTTCTATTTTAGCTTCTATAAATTTTTTTATTACTGTTTTCTTTTTAACTTCTGATGAAGAGGTTTTAAATTTTTTGAAGTATCCTCTTTTTATTATAGGTCAATTAGTTGTAGCTATTTTACTAATATTAACTTTGCCAGTTCTAGCTGCTGCTATTACTATGTTGTTGTTCGATAGAAATTTTAATTCTTGTTTTTTCTCTAACTGAGATGGAGGTGATGTTGTTTTATTTCAGCAC

KT885993_Coeloplana_astericola_Malaysia TCTTTATATTTTTGATTTTCTATATTTGTAGCTTTTATTGCTTTTAGTTATTCTTTTATAATCAGGTTATCTTTAATGTGACCTTATTCTTTCTTGATTGACGGTAATATTTATAATAGTTTTGTGTCTCTTCATGCAATTTTTATGATCTTTTTTTTTGTTATGCCATTTTCCATTGGAGGTTTAGGAAACTGACTTATTCCTTTGTATATAGGTGCTGTTGATATGGCTTTGCCTAGAGTAAATAATTTGTCTTTTTGATTATTATTTGCAGCTTTTATTTTAGGTTTTTTTTCTTCTGTTTTTGCTATGGGTATTCATGCAGGGTGAACAATTTACCCCCCTTTGTCTTCTTATATTGGAAGTCCTAATATTTCTACTGATTTTATAATTTTTTCTTTGCATTGTGCAGGTGCTAGTTCTATTTTAGCTTCTATAAATTTTTTTATTACTGTTTTCTTTTTAACTTCTGATGAAGAGGTTTTAAATTTTTTGAAGTATCCTCTTTTTATTATAGGTCAATTAGTTGTAGCTATTTTACTAATATTAACTTTGCCAGTTCTAGCTGCTGCTATTACTATGTTGTTGTTCGATAGAAATTTTAATTCTTGTTTTTTCTCTAACTGAGATGGAGGTGATGTTGTTTTATTTCAGCAC

KT885994_Coeloplana_green_dots_Red_Sea TCTTTATATTTTTGATTTTCTATATTTGTAGCTTTTATTGCTTTTAGTTATTCTTTTATAATTAGGTTATCTTTGATGTGGCCTTATTCTTTCTTAATTGATGGTAATATTTATAATAGTTTTGTTTCTCTTCATGCAATTTTTATGATTTTTTTTTTCGTTATGCCATTTTCCATTGGAGGTTTAGGAAATTGACTTATTCCTCTATATATAGGTGCAGTTGATATGGCTTTGCCTAGAGTTAATAATTTGTCTTTTTGATTATTATTTGCAGCCTTTATTTTAGGTTTTTTTTCTTCTGTTTTTGCTATGGGTATTCATGCAGGGTGAACAATTTATCCTCCCCTATCTTCTTATATTGGAAGTCCCAACATTTCTACCGATTTTATAATTTTTTCTTTACATTGTGCAGGTGCTAGTTCTATTTTAGCTTCTATAAATTTTTTTATTACTGTCTTTTTTTTAACTTCTGATGAAGAAGTTTTAAATTTTTTAAAGTATCCTCTTTTTATCATAGGTCAATTAGTTGTAGCTATTTTATTAATTTTAACTTTGCCAGTTTTAGCCGCCGCTATTACTATGTTGTTGTTTGATAGAAATTTTAATTCTTGTTTTTTTTCCAATTGAGATGGGGGTGATGTTGTTTTGTTTCAACAT

KT885995_Coeloplana_sp_3_Malaysia TCTTTATATTTTTGATTTTCTATTTTTGTGGCTTTTATTGCTTTTAGTTATTCTTTTATAATTAGGTTATCTTTAATGTGACCTTATTCTTTTTTAATTGATGGTAATATTTACAATAGTTTTGTTTCACTTCATGCAATTTTTATGATTTTTTTTTTTGTAATGCCTTTTTCTATTGGAGGTTTAGGTAATTGACTAATCCCTTTATATATTGGGGCTGTTGATATGGCTCTACCTAGAGTTAATAATTTATCTTTTTGATTATTATTCGCAGCTTTTATTTTGGGTTTTTTTTCTTCTGTTTTCGCTATGGGTATTCATGCAGGTTGAACAATTTATCCACCTCTATCTTCTTACATTGGAAGTCCTAACATTTCCACAGATTTTATAATTTTTTCTTTACATTGTGCAGGTGCTAGTTCTATTTTAGCTTCTATAAATTTTTTTATTACTGTTTTTTTTTTAACTTCTGATGAAGAAGTTTTAAATTTTTTAAAATATCCTCTTTTTATTATAGGTCAACTAGTTGTTGCTATTTTATTAATTTTAACTTTACCAGTTTTGGCTGCTGCTATAACTATGTTATTGTTTGATAGAAATTTTAATTCTTGTTTTTTTTCCAATTGAGATGGGGGTGATGTTGTTTTATTNCAACAT

KT885996_Coeloplana_sp_2_Malaysia TCTTTATATTTTTGATTTTCTATATTTGTAGCTTTTATTGCCTTTAGTTATTCTTTTATAATAAGGTTATCTTTAATGTGACCTTATTCTTTTTTAATTGATGGTAATATTTATAATAGTTTTGTCTCTCTTCATGCAATTTTTATGATTTTTTTTTTTGTTATGCCATTTTCCATTGGAGGTTTAGGAAACTGACTTATTCCTTTATATATAGGTGCTGTCGATATGGCTTTGCCTAGAGTTAATAATTTATCTTTTTGATTATTGTTCGCAGCTTTTATTTTAGGTTTTTTTTCTTCTGTTTTTGCTATGGGTATTCACGCAGGGTGAACAATCTATCCTCCCCTATCTTCTTATATTGGAAGTCCTAACATTTCTACTGATTTTATAATTTTTTCTTTACATTGTGCAGGTGCTAGTTCTATTTTAGCTTCTATAAATTTTTTTATTACTGTTTTTTTTTTAACTTCTGATGAAGAAGTTTTAAATTTTTTGAAGTATCCTCTTTTTATAATAGGTCAATTAGTTGTGGCTATTTTATTAATATTAACTTTGCCAGTTTTAGCTGCTGCTATTACTATGTTGTTGTTTGATAGAAATTTTAATTCTTGTTTTTTTTCTAATTGAGATGGAGGAGATGTTGTTTTATTTCAGCAT

KT885997_Coeloplana_sp_2_Malaysia TCTTTATATTTTTGATTTTCTATATTTGTAGCTTTTATTGCCTTTAGTTATTCTTTTATAATAAGGTTATCTTTAATGTGACCTTATTCTTTTTTAATTGATGGTAATATTTATAATAGTTTTGTCTCTCTTCATGCAATTTTTATGATTTTTTTTTTTGTTATGCCATTTTCCATTGGAGGTTTAGGAAACTGACTTATTCCTTTATATATAGGTGCTGTCGATATGGCTTTGCCTAGAGTTAATAATTTATCTTTTTGATTATTGTTCGCAGCTTTTATTTTAGGTTTTTTTTCTTCTGTTTTTGCTATGGGTATTCACGCAGGGTGAACAATCTATCCTCCCCTATCTTCTTATATTGGAAGTCCTAACATTTCTACTGATTTTATAATTTTTTCTTTACATTGTGCAGGTGCTAGTTCTATTTTAGCTTCTATAAATTTTTTTATTACTGTTTTTTTTTTAACTTCTGATGAAGAAGTTTTAAATTTTTTGAAGTATCCTCTTTTTATAATAGGTCAATTAGTTGTGGCTATTTTATTAATATTAACTTTGCCAGTTTTAGCTGCTGCTATTACTATGTTGTTGTTTGATAGAAATTTTAATTCTTGTTTTTTTTCTAATTGAGATGGAGGAGATGTTGTTTTATTTCAGCAT

KT885998_Coeloplana_sp_3_Malaysia TCTTTATATTTTTGATTTTCTATATTTGTAGCTTTTATTGCCTTTAGTTATTCTTTTATAATAAGGTTATCTTTAATGTGACCTTATTCTTTTTTAATTGATGGTAATATTTATAATAGTTTTGTCTCTCTTCATGCAATTTTTATGATTTTTTTTTTTGTTATGCCATTTTCCATTGGAGGTTTAGGAAACTGACTTATTCCTTTATATATAGGTGCTGTCGATATGGCTTTGCCTAGAGTTAATAATTTATCTTTTTGATTATTGTTCGCAGCTTTTATTTTAGGTTTTTTTTCTTCTGTTTTTGCTATGGGTATTCACGCAGGGTGAACAATCTATCCTCCCCTATCTTCTTATATTGGAAGTCCTAACATTTCTACTGATTTTATAATTTTTTCTTTACATTGTGCAGGTGCTAGTTCTATTTTAGCTTCTATAAATTTTTTTATTACTGTTTTTTTTTTAACTTCTGATGAAGAAGTTTTAAATTTTTTGAAGTATCCTCTTTTTATAATAGGTCAATTAGTTGTGGCTATTTTATTAATATTAACTTTGCCAGTTTTAGCTGCTGCTATTACTATGTTGTTGTTTGATAGAAATTTTAATTCTTGTTTTTTTTCTAATTGAGATGGAGGAGATGTTGTTTTATTTCAGCAT

KT885999_Coeloplana_sp_2_Malaysia TCTTTATATTTTTGATTTTCTATATTTGTAGCTTTTATTGCCTTTAGTTATTCTTTTATAATAAGGTTATCTTTAATGTGACCTTATTCTTTTTTAATTGATGGTAATATTTATAATAGTTTTGTCTCTCTTCATGCAATTTTTATGATTTTTTTTTTTGTTATGCCATTTTCCATTGGAGGTTTAGGAAACTGACTTATTCCTTTATATATAGGTGCTGTCGATATGGCTTTGCCTAGAGTTAATAATTTATCTTTTTGATTATTGTTCGCAGCTTTTATTTTAGGTTTTTTTTCTTCTGTTTTTGCTATGGGTATTCACGCAGGGTGAACAATCTATCCTCCCCTATCTTCTTATATTGGAAGTCCTAACATTTCTACTGATTTTATAATTTTTTCTTTACATTGTGCAGGTGCTAGTTCTATTTTAGCTTCTATAAATTTTTTTATTACTGTTTTTTTTTTAACTTCTGATGAAGAAGTTTTAAATTTTTTGAAGTATCCTCTTTTTATAATAGGTCAATTAGTTGTGGCTATTTTATTAATATTAACTTTGCCAGTTTTAGCTGCTGCTATTACTATGTTGTTGTTTGATAGAAATTTTAATTCTTGTTTTTTTTCTAATTGAGATGGAGGAGATGTTGTTTTATTTCAGCAT

KT886000_Coeloplana_lineolata_Red_Sea TCTTTATATTTTTGATTTTCTATTTTTGTAGCCTTTATTGCTTTTAGTTATTCTTTTATTATTAGGTTATCTTTAATGTGGCCTTATTCTTTTTTAGTCGATGGAAACATTTACAACAGTTTTGTTTCTCTTCATGCCATTTTTATGATTTTTTTTTTTGTAATGCCTTTTTCCATTGGAGGTTTAGGTAATTGACTTATCCCTCTTTATATTGGAGCAGTAGATATGGCTCTTCCTAGAGTTAATAATTTATCCTTTTGGTTATTATTTGCAGCTTTTATCTTAGGTTTTTTTTCTTCAGTCTTTGCTATGGGTATTCATGCTGGTTGAACTATTTATCCTCCTTTATCCTCTTATATTGGCAGTCCCAACATTTCAACTGATTTTATAATTTTTTCTCTCCATTGTGCAGGTGCTAGTTCTATTTTAGCTTCTATTAACTTTTTTATTACTGTTTTTTTCTTAACTTCTGATGAAGAAGTTTTAAATTTTTTAAAATATCCACTTTTTATTATTGGTCAACTTGTAGTGGCTATTCTACTAATTTTGACTCTTCCTGTTTTGGCCGCAGCTATTACTATGCTCTTATTTGATAGAAATTTCAATTCTTGTTTTTTTTCTAATTGAGATGGTGGTGATGTTGTTTTATTTCAACAT

KT886001_Coeloplana_punctata_Red_Sea TCTTTATATTTTTGATTTTCTATTTTTGTAGCCTTTATTGCTTTTAGTTATTCTTTTATTATTAGGTTATCTTTAATGTGGCCTTATTCTTTTTTAGTCGATGGAAACATTTACAACAGTTTTGTTTCTCTTCATGCCATTTTTATGATTTTTTTTTTTGTAATGCCTTTTTCCATTGGAGGTTTAGGTAATTGACTTATCCCTCTTTATATTGGAGCAGTAGATATGGCTCTTCCTAGAGTTAATAATTTATCCTTTTGGTTATTATTTGCAGCTTTTATCTTAGGTTTTTTTTCTTCAGTCTTTGCTATGGGTATTCATGCTGGTTGAACTATTTATCCTCCTTTATCCTCTTATATTGGCAGTCCCAACATTTCAACTGATTTTATAATTTTTTCTCTCCATTGTGCAGGTGCTAGTTCTATTTTAGCTTCTATTAACTTTTTTATTACTGTTTTTTTCTTAACTTCTGATGAAGAAGTTTTAAATTTTTTAAAATATCCACTTTTTATTATTGGTCAACTTGTAGTGGCTATTCTACTAATTTTGACTCTTCCTGTTTTGGCTGCAGCTATTACTATGCTCTTATTTGATAGAAATTTCAATTCTTGTTTTTTTTCTAATTGAGATGGTGGTGATGTTGTTTTATTTCAACAT

KT886002_Coeloplana_punctata_Red_Sea TCTTTATATTTTTGATTTTCTATTTTTGTAGCCTTTATTGCTTTTAGTTATTCTTTTATTATTAGGTTATCTTTAATGTGGCCTTATTCTTTTTTAGTCGATGGAAACATTTACAACAGTTTTGTTTCTCTTCATGCCATTTTTATGATTTTTTTTTTTGTAATGCCTTTTTCCATTGGAGGTTTAGGTAATTGACTTATCCCTCTTTATATTGGAGCAGTAGATATGGCTCTTCCTAGAGTTAATAATTTATCCTTTTGGTTATTATTTGCAGCTTTTATCTTAGGTTTTTTTTCTTCAGTCTTTGCTATGGGTATTCATGCTGGTTGAACTATTTATCCTCCTTTATCCTCTTATATTGGCAGTCCCAACATTTCAACTGATTTTATAATTTTTTCTCTCCATTGTGCAGGTGCTAGTTCTATTTTAGCTTCTATTAACTTTTTTATTACTGTTTTTTTCTTAACTTCTGATGAAGAAGTTTTAAATTTTTTAAAATATCCACTTTTTATTATTGGTCAACTTGTAGTGGCTATTCTACTAATTTTGACTCTTCCTGTTTTGGCTGCAGCTATTACTATGCTCTTATTTGATAGAAATTTCAATTCTTGTTTTTTTTCTAATTGAGATGGTGGTGATGTTGTTTTATTTCAACAT

KT886003_Coeloplana_loyai_Red_Sea TCTTTATATTTTTGATTTTCTATTTTTGTGGCTTTTATTGCTTTTAGTTATTCTTTTATAATTAGGTTATCTTTAATGTGGCCTTATTCTTTTTTAATTGATGGTAACATTTATAATAGTTTTGTTTCTCTTCATGCAATTTTTATGATTTTCTTTTTTGTAATGCCTTTTTCTATTGGAGGTTTAGGTAATTGACTAATCCCTTTATATATTGGGGCTGTTGATATGGCTTTGCCTAGAGTTAATAATTTATCTTTTTGATTATTATTCGCAGCTTTTATTCTGGGTTTTTTTTCTTCTGTTTTTGCTATGGGTATTCATACAGGTTGAACAATTTATCCACCTTTATCTTCTTACATTGGAAGTCCTAACATTTCTACAGATTTTATAATTTTTTCTTTACATTGTGCAGGTGCTAGTTCTATTTTAGCTTCTATAAATTTTTTTATTACTGTTTTTTTTTTAACTTCTGATGAAGAAGTTTTAAATTTTTTAAAGTATCCTCTTTTTATTATAGGTCAACTAGTTGTTGCTATTTTATTAATTTTAACTTTACCAGTTTTAGCTGCTGCTATAACTATGTTATTATTTGATAGAAATTTTAATTCTTGTTTTTTTTCCAATTGAGATGGGGGTGATGTTGTTTTATTTCAACAT

KT886004_Coeloplana_loyai_Red_Sea TCTTTATATTTTTGATTTTCTATTTTTGTGGCTTTTATTGCTTTTAGTTATTCTTTTATAATTAGGTTATCTTTAATGTGGCCTTATTCTTTTTTAATTGATGGTAACATTTATAATAGTTTTGTTTCTCTTCATGCAATTTTTATGATTTTCTTTTTTGTAATGCCTTTTTCTATTGGAGGTTTAGGTAATTGACTAATCCCTTTATATATTGGGGCTGTTGATATGGCTTTGCCTAGAGTTAATAATTTATCTTTTTGATTATTATTCGCAGCTTTTATTCTGGGTTTTTTTTCTTCTGTTTTTGCTATGGGTATTCATACAGGTTGAACAATTTATCCACCTTTATCTTCTTACATTGGAAGTCCTAACATTTCTACAGATTTTATAATTTTTTCTTTACATTGTGCAGGTGCTAGTTCTATTTTAGCTTCTATAAATTTTTTTATTACTGTTTTTTTTTTAACTTCTGATGAAGAAGTTTTAAATTTTTTAAAGTATCCTCTTTTTATTATAGGTCAACTAGTTGTTGCTATTTTATTAATTTTAACTTTACCAGTTTTAGCTGCTGCTATAACTATGTTATTATTTGATAGAAATTTTAATTCTTGTTTTTTTTCCAATTGAGATGGGGGTGATGTTGTTTTATTTCAACAT

KT886005_Coeloplana_loyai_Red_Sea TCTTTATATTTTTGATTTTCTATTTTTGTGGCTTTTATTGCTTTTAGTTATTCTTTTATAATTAGGTTATCTTTAATGTGGCCTTATTCTTTTTTAATTGATGGTAACATTTATAATAGTTTTGTTTCTCTTCATGCAATTTTTATGATTTTCTTTTTTGTAATGCCTTTTTCTATTGGAGGTTTAGGTAATTGACTAATCCCTTTATATATTGGGGCTGTTGATATGGCTTTGCCTAGAGTTAATAATTTATCTTTTTGATTATTATTCGCAGCTTTTATTCTGGGTTTTTTTTCTTCTGTTTTTGCTATGGGTATTCATACAGGTTGAACAATTTATCCACCTTTATCTTCTTACATTGGAAGTCCTAACATTTCTACAGATTTTATAATTTTTTCTTTACATTGTGCAGGTGCTAGTTCTATTTTAGCTTCTATAAATTTTTTTATTACTGTTTTTTTTTTAACTTCTGATGAAGAAGTTTTAAATTTTTTAAAGTATCCTCTTTTTATTATAGGTCAACTAGTTGTTGCTATTTTATTAATTTTAACTTTACCAGTTTTAGCTGCTGCTATAACTATGTTATTATTTGATAGAAATTTTAATTCTTGTTTTTTTTCCAATTGAGATGGGGGTGATGTTGTTTTATTTCAACAT

KT886006_Coeloplana_loyai_Red_Sea TCTTTATATTTTTGATTTTCTATTTTTGTGGCTTTTATTGCTTTTAGTTATTCTTTTATAATTAGGTTATCTTTAATGTGGCCTTATTCTTTTTTAATTGATGGTAACATTTATAATAGTTTTGTTTCTCTTCATGCAATTTTTATGATTTTCTTTTTTGTAATGCCTTTTTCTATTGGAGGTTTAGGTAATTGACTAATCCCTTTATATATTGGGGCTGTTGATATGGCTTTGCCTAGAGTTAATAATTTATCTTTTTGATTATTATTCGCAGCTTTTATTCTGGGTTTTTTTTCTTCTGTTTTTGCTATGGGTATTCATACAGGTTGAACAATTTATCCACCTTTATCTTCTTACATTGGAAGTCCTAACATTTCTACAGATTTTATAATTTTTTCTTTACATTGTGCAGGTGCTAGTTCTATTTTAGCTTCTATAAATTTTTTTATTACTGTTTTTTTTTTAACTTCTGATGAAGAAGTTTTAAATTTTTTAAAGTATCCTCTTTTTATTATAGGTCAACTAGTTGTTGCTATTTTATTAATTTTAACTTTACCAGTTTTAGCTGCTGCTATAACTATGTTATTATTTGATAGAAATTTTAATTCTTGTTTTTTTTCCAATTGAGATGGGGGTGATGTTGTTTTATTTCAACAT

KT886007_Coeloplana_bannwarthi_Red_Sea TCTTTATATTTTTGATTTTCTATTTTTGTAGCCTTTATTGCTTTTAGTTATTCTTTTATTATTAGGTTATCCTTAATGTGGCCTTATTCTTTTTTAGTCGATGGAAACATTTACAACAGTTTTGTTTCTCTTCATGCCATTTTTATGATTTTTTTTTTTGTAATGCCTTTTTCCATTGGAGGTTTAGGTAATTGACTCATTCCTCTTTATATTGGAGCAGTAGATATGGCTCTTCCAAGAGTTAATAATTTATCTTTTTGGTTATTATTTGCAGCTTTTATCTTGGGTTTTTTTTCTTCAGTTTTTGCTATGGGTGTTCACGCTGGTTGAACTATTTACCCTCCTCTATCTTCTTATATTGGCAGTCCTAACATTTCAACTGATTTTATAATTTTTTCTCTCCATTGTGCAGGTGCTAGTTCTATTTTAGCTTCTATTAATTTTTTTATTACTGTTTTTTTTTTAACTTCTGATGAAGAAGTTTTAAATTTTTTAAAGTATCCACTTTTTATTATTGGTCAACTTGTAGTGGCTATTTTACTAATTTTAACTCTTCCTGTCTTAGCCGCAGCTATTACTATGCTCTTATTTGATAGAAATTTTAATTCTTGCTTTTTTTCTAATTGAGATGGCGGTGATGTTGTTTTATTTCAACAT

KT886008_Coeloplana_bannwarthi_Red_Sea TCTTTATATTTTTGATTTTCTATTTTTGTAGCCTTTATTGCTTTTAGTTATTCTTTTATTATTAGGTTATCCTTAATGTGGCCTTATTCTTTTTTAGTCGATGGAAACATTTACAACAGTTTTGTTTCTCTTCATGCCATTTTTATGATTTTTTTTTTTGTAATGCCTTTTTCCATTGGAGGTTTAGGTAATTGACTCATTCCTCTTTATATTGGAGCAGTAGATATGGCTCTTCCAAGAGTTAATAATTTATCTTTTTGGTTATTATTTGCAGCTTTTATCTTGGGTTTTTTTTCTTCAGTTTTTGCTATGGGTGTTCACGCTGGTTGAACTATTTACCCTCCTCTATCTTCTTATATTGGCAGTCCTAACATTTCAACTGATTTTATAATTTTTTCTCTCCATTGTGCAGGTGCTAGTTCTATTTTAGCTTCTATTAATTTTTTTATTACTGTTTTTTTTTTAACTTCTGATGAAGAAGTTTTAAATTTTTTAAAGTATCCACTTTTTATTATTGGTCAACTTGTAGTGGCTATTTTACTAATTTTAACTCTTCCTGTCTTAGCCGCAGCTATTACTATGCTCTTATTTGATAGAAATTTTAATTCTTGCTTTTTTTCTAATTGAGATGGCGGTGATGTTGTTTTATTTCAACAT

KT886009_Coeloplana_bannwarthi_Red_Sea TCTTTATATTTTTGATTTTCTATTTTTGTAGCCTTTATTGCTTTTAGTTATTCTTTTATTATTAGGTTATCCTTAATGTGGCCTTATTCTTTTTTAGTCGATGGAAACATTTACAACAGTTTTGTTTCTCTTCATGCCATTTTTATGATTTTTTTTTTTGTAATGCCTTTTTCCATTGGAGGTTTAGGTAATTGACTCATTCCTCTTTATATTGGAGCAGTAGATATGGCTCTTCCAAGAGTTAATAATTTATCTTTTTGGTTATTATTTGCAGCTTTTATCTTGGGTTTTTTTTCTTCAGTTTTTGCTATGGGTGTTCACGCTGGTTGAACTATTTACCCTCCTCTATCTTCTTATATTGGCAGTCCTAACATTTCAACTGATTTTATAATTTTTTCTCTCCATTGTGCAGGTGCTAGTTCTATTTTAGCTTCTATTAATTTTTTTATTACTGTTTTTTTTTTAACTTCTGATGAAGAAGTTTTAAATTTTTTAAAGTATCCACTTTTTATTATTGGTCAACTTGTAGTGGCTATTTTACTAATTTTAACTCTTCCTGTCTTAGCCGCAGCTATTACTATGCTCTTATTTGATAGAAATTTTAATTCTTGCTTTTTTTCTAATTGAGATGGCGGTGATGTTGTTTTATTTCAACAT

KT886010_Coeloplana_bannwarthi_Red_Sea TCTTTATATTTTTGATTTTCTATTTTTGTAGCCTTTATTGCTTTTAGTTATTCTTTTATTATTAGGTTATCCTTAATGTGGCCTTATTCTTTTTTAGTCGATGGAAACATTTACAACAGTTTTGTTTCTCTTCATGCCATTTTTATGATTTTTTTTTTTGTAATGCCTTTTTCCATTGGAGGTTTAGGTAATTGACTCATTCCTCTTTATATTGGAGCAGTAGATATGGCTCTTCCAAGAGTTAATAATTTATCTTTTTGGTTATTATTTGCAGCTTTTATCTTGGGTTTTTTTTCTTCAGTTTTTGCTATGGGTGTTCACGCTGGTTGAACTATTTACCCTCCTCTATCTTCTTATATTGGCAGTCCTAACATTTCAACTGATTTTATAATTTTTTCTCTCCATTGTGCAGGTGCTAGTTCTATTTTAGCTTCTATTAATTTTTTTATTACTGTTTTTTTTTTAACTTCTGATGAAGAAGTTTTAAATTTTTTAAAGTATCCACTTTTTATTATTGGTCAACTTGTAGTGGCTATTTTACTAATTTTAACTCTTCCTGTCTTAGCCGCAGCTATTACTATGCTCTTATTTGATAGAAATTTTAATTCTTGCTTTTTTTCTAATTGAGATGGCGGTGATGTTGTTTTATTTCAACAT

KT886011_Coeloplana_bannwarthi_Red_Sea TCTTTATATTTTTGATTTTCTATTTTTGTAGCCTTTATTGCTTTTAGCTATTCTTTTATTATTAGGTTATCCTTAATGTGGCCTTATTCTTTTTTAGTCGATGGAAACATTTACAACAGTTTTGTTTCTCTTCATGCCATTTTTATGATTTTTTTTTTTGTAATGCCTTTTTCCATTGGAGGTTTAGGTAATTGACTCATTCCTCTTTATATTGGAGCAGTAGATATGGCTCTTCCAAGAGTTAATAATTTATCTTTTTGGTTATTATTTGCAGCTTTTATCTTGGGTTTTTTTTCTTCAGTTTTTGCTATGGGTGTTCACGCTGGTTGAACTATTTACCCTCCTCTATCTTCTTATATTGGCAGTCCTAACATTTCAACTGATTTTATAATTTTTTCTCTCCATTGTGCAGGTGCTAGTTCTATTTTAGCTTCTATTAATTTTTTTATTACTGTTTTTTTTTTAACTTCTGATGAAGAAGTTTTAAATTTTTTAAAGTATCCACTTTTTATTATTGGTCAACTTGTAGTGGCTATTTTACTAATTTTAACTCTTCCTGTCTTAGCCGCAGCTATTACTATGCTCTTATTTGATAGAAATTTTAATTCTTGCTTTTTTTCTAATTGAGATGGCGGTGATGTTGTTTTATTTCAACAT

KT886012_Coeloplana_bannwarthi_Red_Sea TCTTTATATTTTTGATTTTCTATTTTTGTAGCCTTTATTGCTTTTAGTTATTCTTTTATTATTAGGTTATCCTTAATGTGGCCTTATTCTTTTTTAGTCGATGGAAACATTTACAACAGTTTTGTTTCTCTTCATGCCATTTTTATGATTTTTTTTTTTGTAATGCCTTTTTCCATTGGAGGTTTAGGTAATTGACTCATTCCTCTTTATATTGGAGCAGTAGATATGGCTCTTCCAAGAGTTAATAATTTATCTTTTTGGTTATTATTTGCAGCTTTTATCTTGGGTTTTTTTTCTTCAGTTTTTGCTATGGGTGTTCACGCTGGTTGAACTATTTACCCTCCTCTATCTTCTTATATTGGCAGTCCTAACATTTCAACTGATTTTATAATTTTTTCTCTCCATTGTGCAGGTGCTAGTTCTATTTTAGCTTCTATTAATTTTTTTATTACTGTTTTTTTTTTAACTTCTGATGAAGAAGTTTTAAATTTTTTAAAGTATCCACTTTTTATTATTGGTCAACTTGTAGTGGCTATTTTACTAATTTTAACTCTTCCTGTCTTAGCCGCAGCTATTACTATGCTCTTATTTGATAGAAATTTTAATTCTTGCTTTTTTTCTAATTGAGATGGCGGTGATGTTGTTTTATTTCAACAT

KT886013_Coeloplana_huchonae_Red_Sea TCTTTATATTTTTGATTTTCTATTTTTGTGGCTTTTATTGCTTTTAGTTATTCTTTTATTATAAGGTTGTCTTTAATGTGACCTTATTCTTTCTTAGTTGATGGTAACATTTATAATAGTTTTGTTTCTCTTCATGCTATTTTTATGATTTTTTTCTTTGTTATGCCTTTTTCTATTGGAGGTTTAGGTAACTGACTTATTCCTCTCTATATTGGAGCTGTAGACATGGCTCTTCCCAGAATTAATAATTTATCTTTTTGGTTATTATTTGCAGCTTTTATTTTAGGTTTTTTTTCTTCAGTCTTTGCTATGGGTATTCACGCTGGTTGAACTATTTATCCTCCTCTATCCTCTTATATAGGTAGTCCTAACATTTCAACTGATTTTATAATTTTTTCTCTTCATTGTGCAGGTGCTAGTTCTATTTTAGCTTCTATTAATTTTTTTATTACTGTTTTTTTTTTAACTTCTGATGAAGAAGTTTTAAATTTTTTAAAGTATCCACTTTTTATTATTGGTCAACTTGTAGTGGCTATTTTATTAATTTTAACACTTCCTGTATTAGCTGCAGCTATCACTATGTTATTATTTGATAGAAATTTTAATTCTTGTTTTTTTTCTAATTGAGATGGAGGTGATGTTGTTTTGTTTCAACAT

KT886014_Coeloplana_huchonae_Red_Sea TCTTTATATTTTTGATTTTCTATTTTTGTGGCTTTTATTGCTTTTAGTTATTCTTTTATTATAAGGTTGTCTTTAATGTGACCTTATTCTTTCTTAGTTGATGGTAACATTTATAATAGTTTTGTTTCTCTTCATGCTATTTTTATGATTTTTTTCTTTGTTATGCCTTTTTCTATTGGAGGTTTAGGTAACTGACTTATTCCTCTCTATATTGGAGCTGTAGACATGGCTCTTCCCAGAATTAATAATTTATCTTTTTGGTTATTATTTGCAGCTTTTATTTTAGGTTTTTTTTCTTCAGTCTTTGCTATGGGTATTCACGCTGGTTGAACTATTTATCCTCCTCTATCCTCTTATATAGGTAGTCCTAACATTTCAACTGATTTTATAATTTTTTCTCTTCATTGTGCAGGTGCTAGTTCTATTTTAGCTTCTATTAATTTTTTTATTACTGTTTTTTTTTTAACTTCTGATGAAGAAGTTTTAAATTTTTTAAAGTATCCACTTTTTATTATTGGTCAACTTGTAGTGGCTATTTTATTAATTTTAACACTTCCTGTATTAGCTGCAGCTATCACTATGTTATTATTTGATAGAAATTTTAATTCTTGTTTTTTTTCTAATTGAGATGGAGGTGATGTTGTTTTGTTTCAACAT

KT886015_Coeloplana_huchonae_Red_Sea TCTTTATATTTTTGATTTTCTATTTTTGTGGCTTTTATTGCTTTTAGTTATTCTTTTATTATAAGGTTGTCTTTAATGTGACCTTATTCTTTCTTAGTTGATGGTAACATTTATAATAGTTTTGTTTCTCTTCATGCTATTTTTATGATTTTTTTCTTTGTTATGCCTTTTTCTATTGGAGGTTTAGGTAACTGACTTATTCCTCTCTATATTGGAGCTGTAGACATGGCTCTTCCCAGAATTAATAATTTATCTTTTTGGTTATTATTTGCAGCTTTTATTTTAGGTTTTTTTTCTTCAGTCTTTGCTATGGGTATTCACGCTGGTTGAACTATTTATCCTCCTCTATCCTCTTATATAGGTAGTCCTAACATTTCAACTGATTTTATAATTTTTTCTCTTCATTGTGCAGGTGCTAGTTCTATTTTAGCTTCTATTAATTTTTTTATTACTGTTTTTTTTTTAACTTCTGATGAAGAAGTTTTAAATTTTTTAAAGTATCCACTTTTTATTATTGGTCAACTTGTAGTGGCTATTTTATTAATTTTAACACTTCCTGTATTAGCTGCAGCTATCACTATGTTATTATTTGATAGAAATTTTAATTCTTGTTTTTTTTCTAATTGAGATGGAGGTGATGTTGTTTTGTTTCAACAT

KT886016_Coeloplana_huchonae_Red_Sea TCTTTATATTTTTGATTTTCTATTTTTGTGGCTTTTATTGCTTTTAGTTATTCTTTTATTATAAGGTTGTCTTTAATGTGACCTTATTCTTTCTTAGTTGATGGTAACATTTATAATAGTTTTGTTTCTCTTCATGCTATTTTTATGATTTTTTTCTTTGTTATGCCTTTTTCTATTGGAGGTTTAGGTAACTGACTTATTCCTCTCTATATTGGAGCTGTAGACATGGCTCTTCCCAGAATTAATAATTTATCTTTTTGGTTATTATTTGCAGCTTTTATTTTAGGTTTTTTTTCTTCAGTCTTTGCTATGGGTATTCACGCTGGTTGAACTATTTATCCTCCTCTATCCTCTTATATAGGTAGTCCTAACATTTCAACTGATTTTATAATTTTTTCTCTTCATTGTGCAGGTGCTAGTTCTATTTTAGCTTCTATTAATTTTTTTATTACTGTTTTTTTTTTAACTTCTGATGAAGAAGTTTTAAATTTTTTAAAGTATCCACTTTTTATTATTGGTCAACTTGTAGTGGCTATTTTATTAATTTTAACACTTCCTGTATTAGCTGCAGCTATCACTATGTTATTATTTGATAGAAATTTTAATTCTTGTTTTTTTTCTAATTGAGATGGAGGTGATGTTGTTTTGTTTCAACAT

KT886017_Coeloplana_huchonae_Red_Sea TCTTTATATTTTTGATTTTCTATTTTTGTGGCTTTTATTGCTTTTAGTTATTCTTTTATTATAAGGTTGTCTTTAATGTGACCTTATTCTTTCTTAGTTGATGGTAACATTTATAATAGTTTTGTTTCTCTTCATGCTATTTTTATGATTTTTTTCTTTGTTATGCCTTTTTCTATTGGAGGTTTAGGTAACTGACTTATTCCTCTCTATATTGGAGCTGTAGACATGGCTCTTCCCAGAATTAATAATTTATCTTTTTGGTTATTATTTGCAGCTTTTATTTTAGGTTTTTTTTCTTCAGTCTTTGCTATGGGTATTCACGCTGGTTGAACTATTTATCCTCCTCTATCCTCTTATATAGGTAGTCCTAACATTTCAACTGATTTTATAATTTTTTCTCTTCATTGTGCAGGTGCTAGTTCTATTTTAGCTTCTATTAATTTTTTTATTACTGTTTTTTTTTTAACTTCTGATGAAGAAGTTTTAAATTTTTTAAAGTATCCACTTTTTATTATTGGTCAACTTGTAGTGGCTATTTTATTAATTTTAACACTTCCTGTATTAGCTGCAGCTATCACTATGTTATTATTTGATAGAAATTTTAACTCTTGTTTTTTTTCTAATTGAGATGGAGGTGATGTTGTTTTGTTTCAACAT

KT886018_Coeloplana_bannwarthi_var_Red_Sea TCTTTATATTTTTGATTTTCTATTTTTGTAGCATTTATTGCTTTTAGTTATTCTTTTATTATTAGGTTATCTTTAATGTGGCCTTATTCTTTTTTAGTTGATGGAAACATTTACAACAGTTTTGTTTCTCTTCATGCCATTTTTATGATTTTTTTTTTTGTAATGCCTTTTTCTATTGGAGGTTTAGGTAATTGACTTATTCCTCTTTATATTGGAGCAGTAGATATGGCTCTTCCTAGAGTTAATAATTTATCCTTTTGGTTATTATTTGCAGCTTTTGTATTAGGTTTTTTTTCTTCAGTTTTTGCTATGGGTATTCACGCTGGTTGAACTATTTACCCTCCTTTATCTTCCTATATTGGTAGTCCTAACATTTCAACTGATTTTGTAATTTTTTCTCTCCATTGTGCAGGTGCTAGTTCTATTTTAGCTTCTATTAATTTTTTTATTACTGTTTTTTTTTTAACTTCTGATGAAGAAGTTTTAAATTTTTTAAAGTATCCACTTTTTATTATTGGTCAACTTGTAGTGTCTATTTTATTAATTTTAACTCTTCCTGTCTTGGCCGCAGCTATTACTATGCTCTTATTTGATAGAAATTTTAATTCTTGTTTTTTTTCTAATTGAGATGGTGGTGATGTTGTTTTATTTCAACAT

KT886019_Coeloplana_yulianicorum_Red_Sea TCTTTATATTTTTGATTTTCTATTTTTGTAGCTTTCATTGCTTTTAGCTATTCTTTTATTATAAGATTATCCTTAATGTGACCCTACTCATTCTTAGTGGATGGAAACATCTACAATAGCTTTGTTTCTTTACATGCTATTTTTATGATTTTTTTTTTTGTTATGCCTTTCTCAATAGGGGGTCTAGGTAATTGACTTATTCCCCTTTACATTGGAGCTGTAGATATGGCTCTTCCTAGAGTTAACAATTTATCTTTTTGGTTATTGTTTGCAGCTTTTATATTGGGTTTCTTTTCTTCTGTGTTTGCCATGGGTATTCATGCAGGTTGAACCATCTATCCTCCTTTGTCTTCCTACATTGGAAGTCCTAACATTTCTACAGATTTTGTAATTTTTTCACTTCACTGTGCAGGTGCTAGTTCAATTTTAGCTTCTATAAATTTTTTTATTACTGTTTTTTTTTTAACTTCTGATGAAGAAGTCCTAAATTTTTTAAAATATCCTCTTTTTATCATTGGTCAATTAGTGGTGGCCATTCTTTTAATCTTAACTCTTCCAGTACTGGCTGCAGCTATTACTATGTTATTATTTGACAGAAATTTTAATTCTTGTTTTTTTTCTAATTGAGATGGGGGAGATGTTGTTCTTTTTCAACAT

KT886020_Coeloplana_yulianicorum_Red_Sea TCTTTATATTTTTGATTTTCTATTTTTGTAGCTTTCATTGCTTTTAGCTATTCTTTTATTATAAGATTATCCTTAATGTGACCCTACTCATTCTTAGTGGATGGAAACATCTACAATAGCTTTGTTTCTTTACATGCTATTTTTATGATTTTTTTTTTTGTTATGCCTTTCTCAATAGGGGGTCTAGGTAATTGACTTATTCCCCTTTACATTGGAGCTGTAGATATGGCTCTTCCTAGAGTTAACAATTTATCTTTTTGGTTATTGTTTGCAGCTTTTATATTGGGTTTCTTTTCTTCTGTGTTTGCCATGGGTATTCATGCAGGTTGAACCATCTATCCTCCTTTGTCTTCCTACATTGGAAGTCCTAACATTTCTACAGATTTTGTAATTTTTTCACTTCACTGTGCAGGTGCTAGTTCAATTTTAGCTTCTATAAATTTTTTTATTACTGTTTTTTTTTTAACTTCTGATGAAGAAGTCCTAAATTTTTTAAAATACCCTCTTTTTATCATTGGTCAATTAGTGGTGGCCATTCTTTTAATCTTAACTCTTCCAGTACTGGCTGCAGCTATTACTATGTTGTTATTTGACAGAAATTTTAATTCTTGTTTTTTTTCTAATTGAGATGGGGGAGATGTTGTTCTTTTTCAACAT

HQ435815_Coeloplana_bocki TCTTTATATTTTTGATTTTCTATTTTTGTTGCTTTTATTGCTTTTAGTTATTCTTTTATTATTAGATTATCTTTAATGTGGCCTTATTCTTTTTTAGTAGATGGTAATATATATAATAGTTTTGTTTCTTTGCATGCTATTTTTATGATTTTTTTTTTTGTTATGCCATTTTCTATTGGAGGTTTAGGTAATTGATTAATACCCTTATATATAGGGGCTGTAGACATGGCCCTACCTAGGGTGAATAATTTATCTTTTTGGTTGTTATTTGCAGCTTTTATTTTGGGTTTTTTTTCTTCTGTTTTTGCCATGGGTATACACGCTGGTTGAACGATTTACCCACCCTTATCTTCCTATATTGGAAGTCCTAATATTTCTACAGATTTTATAATTTTTTCTCTCCATTGTGCAGGTGCTAGTTCTATTTTAGCTTCTATAAATTTTTTTATTACTGTTTTTTTTTTAACTTCAGATGAAGAAGTTTTAAATTTTTTAAAATATCCTTTATTTATTATTGGTCAGTTAGTTGTTGCTATTTTATTAATATTGACATTGCCTGTTTTGGCTGCAGCTATTACAATGTTATTATTTGATAGGAATTTTAATTCTTGTTTTTTTTCTAACTGAGATGGGGGGGACGCTGTTTTGTTTCAACAT

KT886021_Coeloplana_loyai_Red_Sea TCTTTATATTTTTGATTTTCTATTTTTGTGGCTTTTGTTGCTTTTAGTTATTCTTTTATAATTAGGTTATCTTTAATGTGACCTTATTCTTTTTTAATTGATGGTAACATTTATAATAGTTTTGTTTCTCTTCATGCAATTTTTATGATTTTTTTTTTCGTAATGCCTTTTTCTATTGGAGGTTTAGGTAATTGACTAATCCCTTTATATATTGGGGCTGTTGATATGGCTTTGCCTAGAGTTAATAATTTATCTTTTTGATTATTATTCGTAGCTTTTATTCTGGGTTTTTTTTCTTCTGTTTTTGCTATGGGTATTCATGCAGGTTGAACAATTTATCCACCTTTATCTTCTTACATTGGAAGTCCTAACATTTCTACAGATTTTATAATTTTTTCTTTACATTGTGCAGGTGCTAGTTCTATTTTAGCTTCTATAAATTTTTTTATTACTGTTTTTTTTTTAACTTCTGATGAAGAAGTTTTAAATTTTTTAAAGTATCCTCTTTTTATTATAGGTCAACTAGTTGTTGCTATTTTATTAATTTTAACTTTACCAGTTTTAGCTGCTGCTATAACTATGTTATTATTTGATAGAAATTTTAATTCTTGTTTTTTTTCCAATTGAGATGGGGGTGATGTTGTTTTATTTCAACAT

KT886022_Vallicula_multiformis_Red_Sea TCTTTATATTTTTTTTTTGCTATTTTTATGGCTTTTGTAGCTTTTGGTTATTCTGCTTCTATTAGATATTCCTTATTATGACCTGTAGCTTTTATAGATTTTGCTAATATTTATAATAATGCTGTTACTTTACATGCTATTTTTATGATTTTTTTTTTTGTTATGCCATTTAGTATTGGTGGTTTAGGTAATTGATTAATTCCTTTATTTTTAGGGGTTATAGATATGAGTTTGCCTAGAATTAATAATTTATCTTTTTGATTATTATTTTTTTCTTTTATGATATCTTTGTTTCATTTATTTACTTATGATGGTGTTTTCACTGGTTGAACAATTTATCCTCCTTTGTCTGGTCCTGAAGGTTCAATCACTACTGCTGTTGATTATATAATTTTTTCTTTACATTTAGCTGGTGCTAGTAGTATTTTAGCTTCAATAAATTTTTTTATAACTATTTTTTTTATGTTTTTAAATAATTCAGTTTTCAATTTTTTTAAAGTTCCTTTATTTATAATTGGTCAATTAGTTGTTGCTTTTCTTTTAGTTTTATCTTTGCCTGTTTTAGCTGCTGCTATAACTATGCTTTTATTTGATAGAAATTTTAATACTTGTTTTTTTTCTAATTATTGAGGTGGTGATGCTGTTTTATTTCAACAT

KT886023_Vallicula_multiformis_Red_Sea TCTTTATATTTTTTTTTTGCTATTTTTATGGCTTTTGTAGCTTTTGGTTATTCTGCTTCTATTAGATATTCCTTATTATGACCTGTAGCTTTTATAGATTTTGCTAATATTTATAATAATGCTGTTACTTTACATGCTATTTTTATGATTTTTTTTTTTGTTATGCCATTTAGTATTGGTGGTCTAGGTAATTGATTAATTCCTTTATTTTTAGGGGTTATAGATATGAGTTTGCCTAGAATTAATAATTTATCTTTTTGATTATTATTTTTTTCTTTTATGATATCTTTGTTTCATTTATTTACTTATGATGGTGTTTTCACTGGTTGAACAATTTATCCTCCTCTGTCTGGTCCTGAAGGTTCAATTACTACTGCTGTTGATTATATAATTTTTTCTTTACATTTAGCTGGTGCTAGTAGTATTTTAGCTTCAATAAATTTTTTTATAACTATTTTTTTTATGTTTTTAAATAATTCAGTTTTCAATTTTTTTAAAGTTCCTTTATTTATAATTGGTCAATTAGTTGTTGCTTTTCTTTTAGTTTTATCTTTGCCTGTTTTAGCTGCTGCTATAACTATGCTTTTATTTGATAGAAATTTTAATACTTGTTTTTTTTCTAATTATTGAGGTGGTGATGCTGTTTTATTTCAACAT

KT886024_Vallicula_multiformis_Red_Sea TCTTTATATTTTTTTTTTGCTATTTTTATGGCTTTTGTAGCTTTTGGTTATTCTGCTTCTATTAGATATTCCTTATTATGACCTGTAGCTTTTATAGATTTTGCTAATATTTATAATAATGCTGTTACTTTACATGCTATTTTTATGATTTTTTTTTTTGTTATGCCATTTAGTATTGGTGGTCTAGGTAATTGATTAATTCCTTTATTTTTAGGGGTTATAGATATGAGTTTGCCTAGAATTAATAATTTATCTTTTTGATTATTATTTTTTTCTTTTATGATATCTTTGTTTCATTTATTTACTTATGATGGTGTTTTCACTGGTTGAACAATTTATCCTCCTCTGTCTGGTCCTGAAGGTTCAATTACTACTGCTGTTGATTATATAATTTTTTCTTTACATTTAGCTGGTGCTAGTAGTATTTTAGCTTCAATAAATTTTTTTATAACTATTTTTTTTATGTTTTTAAATAATTCAGTTTTCAATTTTTTTAAAGTTCCTTTATTTATAATTGGTCAATTAGTTGTTGCTTTTCTTTTAGTTTTATCTTTGCCTGTTTTAGCTGCTGCTATAACTATGCTTTTATTTGATAGAAATTTTAATACTTGTTTTTTTTCTAATTATTGAGGTGGTGATGCTGTTTTATTTCAACAT

KT886025_Vallicula_multiformis_Red_Sea TCTTTATATTTTTTTTTTGCTATTTTTATGGCTTTTGTAGCTTTTGGTTATTCTGCTTCTATTAGATATTCCTTATTATGACCTGTAGCTTTTATAGATTTTGCTAATATTTATAATAATGCTGTTACTTTACATGCTATTTTTATGATTTTTTTTTTTGTTATGCCATTTAGTATTGGTGGTCTAGGTAATTGATTAATTCCTTTATTTTTAGGGGTTATAGATATGAGTTTGCCTAGAATTAATAATTTATCTTTTTGATTATTATTTTTTTCTTTTATGATATCTTTGTTTCATTTATTTACTTATGATGGTGTTTTCACTGGTTGAACAATTTATCCTCCTCTGTCTGGTCCTGAAGGTTCAATTACTACTGCTGTTGATTATATAATTTTTTCTTTACATTTAGCTGGTGCTAGTAGTATTTTAGCTTCAATAAATTTTTTTATAACTATTTTTTTTATGTTTTTAAATAATTCAGTTTTCAATTTTTTTAAAGTTCCTTTATTTATAATTGGTCAATTAGTTGTTGCTTTTCTTTTAGTTTTATCTTTGCCTGTTTTAGCTGCTGCTATAACTATGCTTTTATTTGATAGAAATTTTAATACTTGTTTTTTTTCTAATTATTGAGGTGGTGATGCTGTTTTATTTCAACAT

KT886026_Vallicula_multiformis_Red_Sea TCTTTATATTTTTTTTTTGCTATTTTTATGGCTTTTGTAGCTTTTGGTTATTCTGCTTCTATCAGATATTCCTTATTATGACCTGTAGCTTTCATAGATTTTGCTAATATTTATAATAATGCTGTTACTTTGCATGCTATTTTTATGATTTTTTTTTTTGTTATGCCATTTAGTATTGGTGGTCTAGGTAATTGATTAATTCCTTTATTTTTAGGGGTTATAGATATGAGTTTGCCTAGAATTAATAATTTATCTTTTTGATTATTATTTTTTTCTTTTATGATATCTTTGTTTCATTTATTTACTTATGATGGTGTTTTCACTGGTTGAACAATTTATCCCCCTCTGTCTGGTCCTGAAGGTTCAATCACTACTGCTGTTGATTATATAATTTTTTCTTTACATTTAGCTGGTGCTAGTAGTATTTTAGCTTCAATAAATTTTTTTATAACTATTTTTTTTATGTTTTTAAATAATTCAGTTTTTAATTTTTTTAAAGTTCCTTTATTTATAATTGGTCAATTAGTTGTTGCTTTTCTTTTAGTTTTATCTTTGCCTGTTTTAGCTGCTGCTATAACTATGCTTTTATTTGATAGAAATTTTAATACTTGTTTTTTTTCTAATTATTGAGGTGGTGATGCTGTTTTATTTCAACAT

KT886027_Vallicula_multiformis_Red_Sea TCTTTATATTTTTTTTTTGCTATTTTTATGGCTTTTGTAGCTTTTGGTTATTCTGCTTCTATCAGATATTCCTTATTATGACCTGTAGCTTTCATAGATTTTGCTAATATTTATAATAATGCTGTTACTTTGCATGCTATTTTTATGATTTTTTTTTTTGTTATGCCATTTAGTATTGGTGGTCTAGGTAATTGATTAATTCCTTTATTTTTAGGGGTTATAGATATGAGTTTGCCTAGAATTAATAATTTATCTTTTTGATTATTATTTTTTTCTTTTATGATATCTTTGTTTCATTTATTTACTTATGATGGTGTTTTCACTGGTTGAACAATTTATCCCCCTCTGTCTGGTCCTGAAGGTTCAATCACTACTGCTGTTGATTATATAATTTTTTCTTTACATTTAGCTGGTGCTAGTAGTATTTTAGCTTCAATAAATTTTTTTATAACTATTTTTTTTATGTTTTTAAATAATTCAGTTTTTAATTTTTTTAAAGTTCCTTTATTTATAATTGGTCAATTAGTTGTTGCTTTTCTTTTAGTTTTATCTTTGCCTGTTTTAGCTGCTGCTATAACTATGCTTTTATTTGATAGAAATTTTAATACTTGTTTTTTTTCTAATTATTGAGGTGGTGATGCTGTTTTATTTCAACAT

;

end;
